# Ligand substituents modulate excited-state lifetime and energy-transfer reactivity in Cu(i) photosensitizers supported by salicylaldimine and isocyanide ligands

**DOI:** 10.1039/d5sc07286g

**Published:** 2025-10-27

**Authors:** Soumi Chakraborty, Kianna Agyekum, Dooyoung Kim, Thomas S. Teets

**Affiliations:** a Department of Chemistry, University of Houston Houston Texas 77204 USA tteets@uh.edu

## Abstract

The design of earth-abundant molecular photosensitizers with desirable photophysical properties and good excited-state reactivity is critical for sustainable photochemical applications. Herein, we report a new family of three-coordinate heteroleptic Cu(i) complexes supported by monoanionic salicylaldimine (N^O) chelating ligands and aryl isocyanides. By systematically tuning the steric bulk on each ligand, we establish clear structure–property relationships that govern the excited-state lifetimes and photocatalytic performance metrics of these complexes. Increasing steric congestion on the salicylaldimine ligand, which contributes to the HOMO, results in faster nonradiative decay and shortens excited-state lifetimes. In contrast, introducing steric bulk on the isocyanide ligand, where the LUMO is primarily localized, suppresses nonradiative decay, most likely by inhibiting excited-state geometric relaxation, thereby extending the lifetime up to 375 ns. These photophysical trends correlate directly with performance in triplet–triplet energy transfer (TTET) photocatalysis, where longer-lived complexes enable faster *E*/*Z* isomerization of *trans*-stilbene. This work demonstrates that remote steric modulation of ligand frameworks offers a simple yet powerful strategy for tuning the excited-state dynamics and catalytic properties of this new class of Cu(i) photosensitizers.

## Abstract 2

Remote steric tuning of Cu(i) complexes controls excited-state dynamics and enhances photocatalytic triplet energy transfer efficiency.

## Graphical abstract

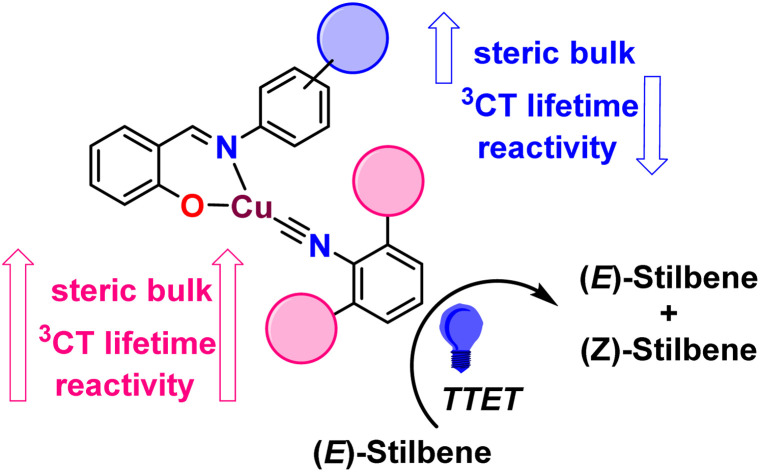

## Introduction

Molecular photosensitizers are central to a broad spectrum of technologies, such as optical devices, photodynamic therapy, materials science and synthetic chemistry.^1–6^ Many of the foundational developments in molecular photosensitizers centered on precious metals such as ruthenium, iridium, and platinum, platforms which continue to proliferate in fundamental research and applications to this day. However, due to the high cost, limited availability, and environmental concerns associated with heavy transition metals, there is a pressing need to develop cost-effective molecular photosensitizers based on more abundant and sustainable alternatives. In this context, first-row transition metals—often referred to as base metals—have garnered significant attention over the past few decades for their potential to replace noble metals in a variety of applications, including solar energy conversion, catalysis, and photomedicine.^7–14^

Many photosensitizers based on first-row transition metals are limited by short excited-state lifetimes due to thermally accessible metal-centered (MC) excited states derived from partially filled 3d orbitals, which facilitate nonradiative deactivation and quench luminescence.^10^ To avoid this limitation altogether, metals with a d^10^ electronic configuration, such as copper(i) and zinc(ii), can be used.^10,15–19^ Among these, copper(i) complexes have garnered significant attention during recent decades due to their favorable photophysical properties and accessible redox chemistry.^10^ They have been widely used in dye-sensitized solar cells (DSSCs), light-emitting devices, and photocatalysis.^10,17,20–28^ The versatility and tunability of copper(i) coordination environments make them promising candidates for next-generation photosensitizers in sustainable energy and optoelectronic applications.^10^

Despite their d^10^ configuration and lack of MC states, it is nonetheless a challenge to obtain long charge-transfer (CT) excited-state lifetimes in Cu(i) photosensitizers, owing to pseudo Jahn–Teller distortions that lead to excited-state energy loss and fast nonradiative decay. A widely adopted strategy to extend the excited-state lifetimes of Cu(i) complexes, most prominently developed on four-coordinate bis-chelate complexes, involves sterically encumbered ligands which prevent the excited-state structural distortions.^10,20,29^ A recent advance in copper(i) photosensitizer design, driven in large part by our own group's efforts, involves heteroleptic architectures that pair an electron-rich anionic ligand with a neutral π-acceptor ligand, involving a variety of coordination numbers and structure types.^10,30–37^ With two different ligands that can be independently modified, an important line of inquiry that emerged in the early work on heteroleptic photosensitizers is determining which ligand's steric profile is more important in dictating the observed excited-state decay dynamics, and whether steric modifications to both ligands can synergistically combine to lengthen the lifetime. With four-coordinate copper(i) β-diketiminate 1,10-phenanthroline complexes, we examined steric effects *via* alkyl substitution, and found that modification of both ligands contributed to substantial increases in the lifetime of the ^3^CT state, albeit with a ceiling of ∼2 ns and a larger impact of the 1,10-phenanthroline steric profile.^36^ More recently, in three-coordinate copper(i) β-diketiminate isocyanide complexes, we showed that steric augmentation of the isocyanide ligand, keeping the β-diketiminate constant, was effective at eliciting a *ca.* 30-fold increase in ^3^CT lifetime, reaching 276 ns in one example.^37^ Thus, it seems that increased ligand steric bulk, demonstrated as a robust strategy to improve ^3^CT lifetimes in some of the earliest known examples of copper(i) charge-transfer chromophores, remains a viable approach in some of the most recently discovered classes of complexes and warrants continued investigation.

With this motivation, herein we report a new series of three-coordinate heteroleptic Cu(i) complexes (Cu1–Cu7) featuring bidentate monoanionic salicylaldimine (N^O-chelate) ligands in combination with aryl isocyanides. A key distinction of these complexes, compared to our other recent work, is that the monoanionic L^X chelating ligand is electronically and sterically asymmetric, leaving it an open question whether this class of compounds would give analogous charge-transfer transitions, and whether ligand steric modifications would be as impactful in imparting long excited-state lifetimes. Comprehensive photophysical studies reveal that increasing the steric bulk on the salicylaldimine ligand, where the HOMO is primarily localized, leads to a marked decrease in excited-state lifetimes (Cu1–Cu4). Conversely, increasing steric bulk on the aryl isocyanide ligand, where the LUMO is primarily localized, results in a notable increase in excited-state lifetimes (Cu5–Cu7), reaching up to 375 ns. Beyond demonstrating an increase in excited-state lifetime, this study assesses the photocatalytic performance of these Cu(i) complexes in the *E*/*Z* isomerization of *trans*-stilbene *via* triplet–triplet energy transfer (TTET). A clear correlation is observed between photocatalytic rate and excited-state lifetime: complexes with extended lifetimes displayed significantly accelerated reaction rates. Thus, the present study introduces a new class of copper(i) heteroleptic photosensitizers and puts forward a broad conceptual advance, showing that systematic variation of the steric environment in salicylaldimine–isocyanide Cu(i) complexes simultaneously governs excited-state lifetime, excited-state energy, and photocatalytic reactivity. These findings highlight the critical role of ligand design in tuning the excited-state properties of this new class of copper-based photosensitizers.

## Results and discussion

### Synthesis

**Scheme 1:**
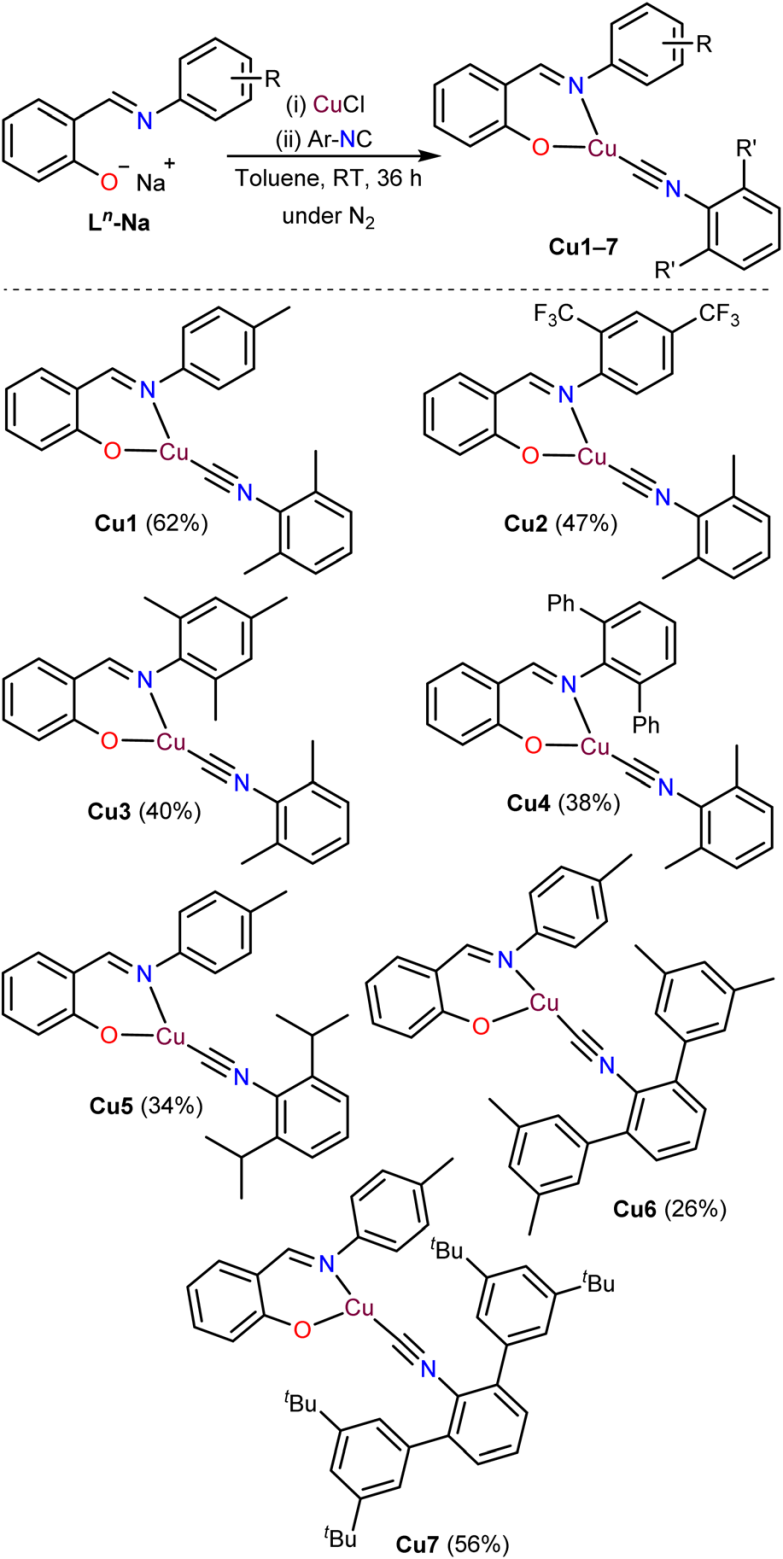
Synthesis of Cu(i) complexes using salicylaldimine chelating ligands and aryl isocyanides.

The synthesis of the three-coordinate Cu(i) complexes Cu1–Cu7, featuring structurally diverse salicylaldimine and aryl isocyanide (Ar-NC) ligands, is outlined in Scheme 1. Detailed synthetic procedures are provided in the SI. Both the salicylaldimine proligands (L*^n^*H) and the aryl isocyanides bearing various substituents were prepared according to reported literature procedures (see SI). In an N_2_-filled glovebox, the neutral L*^n^*H proligands were deprotonated using NaHMDS (1 : 1 molar ratio) in diethyl ether to yield the corresponding sodium salicylaldiminate salts (L*^n^*-Na). These salts were treated with CuCl (1 : 1 ratio) in toluene and stirred for 3 hours, after which the respective aryl isocyanide (0.7 equiv.) was added. A sub-stoichiometric amount of the aryl isocyanide ligand was intentionally employed to prevent the formation of four-coordinate Cu(i) complexes bearing two isocyanide ligands, a common side product observed both in the present study and in previously reported heteroleptic Cu(i) systems.^37^ The Cu(i) complexes were obtained in moderate isolated yields (26–62%) after purification by recrystallization. All synthesized complexes were characterized by ^1^H and ^13^C{^1^H} NMR spectroscopy, and additionally ^19^F NMR spectroscopy for Cu2. The corresponding spectra (Fig. S30–S44) confirm the structural integrity of the compounds.

### Structural characterization

**Fig. 1:**
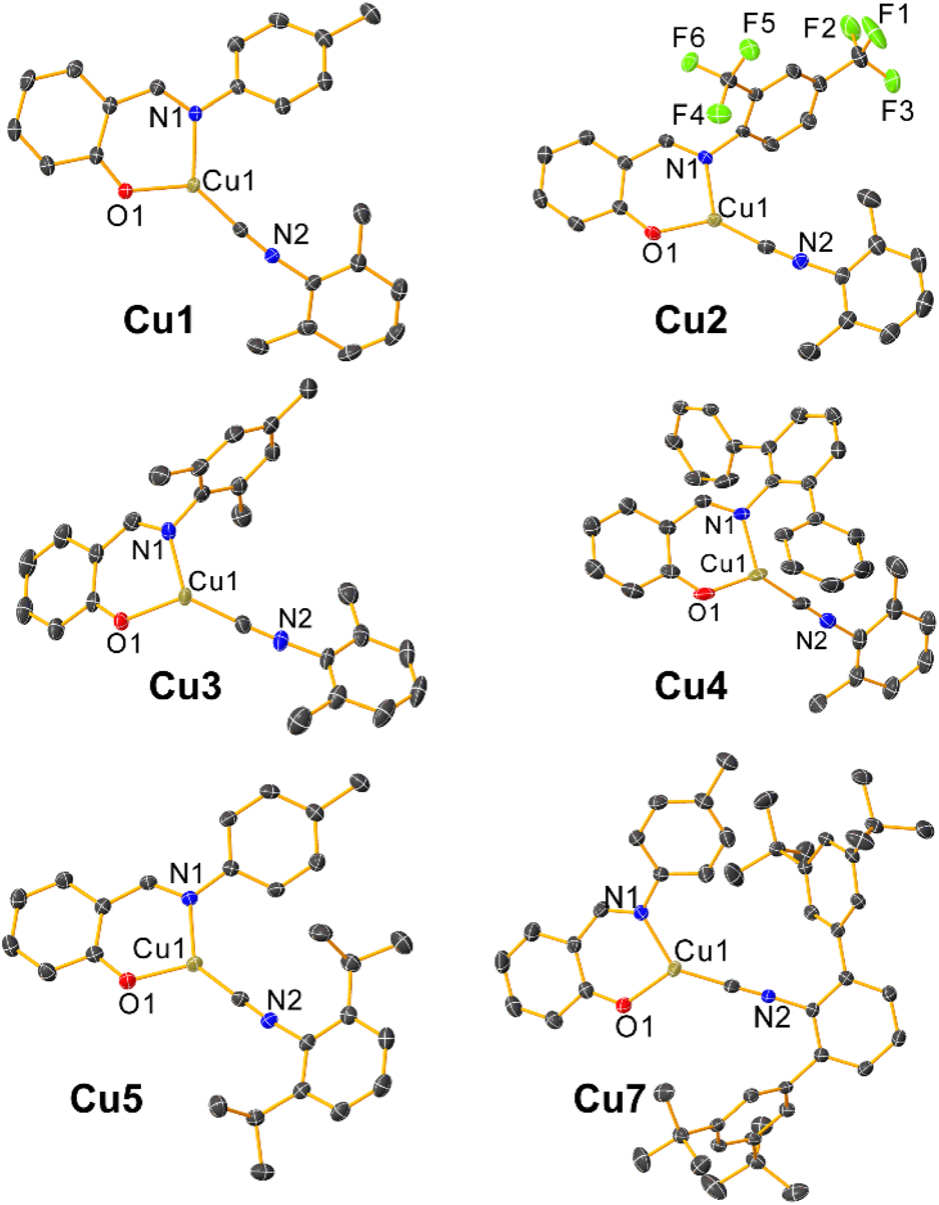
Molecular structures of Cu1–Cu5 and Cu7 determined by single crystal X-ray diffraction. Thermal ellipsoids are shown at the 50% probability level. H-atoms are removed for visual clarity.

Complexes Cu1–Cu5 and Cu7 were further characterized by single-crystal X-ray diffraction, and their molecular structures are depicted in Fig. 1. Crystallographic refinement details are summarized in Tables S1–S3, while selected bond lengths and angles involving the Cu center are compiled in Table S4. Despite multiple attempts, single crystals suitable for X-ray analysis could not be obtained for Cu6. In all cases except Cu3, the complexes adopt a planar, distorted Y-shaped geometry around the copper center, with the sum of the bond angles approximating 360° (Table S4). In two cases, significant intermolecular interactions support dimeric structures in the solid state. In Cu2, the two crystallographically independent molecules each dimerize with symmetry-generated equivalents, *via* cuprophilic interactions with internuclear distances of *ca.* 3.0 Å (Fig. S1). Cu3 is the only complex where the solid-state coordination geometry deviates from planarity, approaching distorted tetrahedral on account of a dimeric assembly stabilized by weak intermolecular Cu⋯O interactions [Cu1–O1: 2.015(2) Å; intermolecular Cu1⋯O1: 2.242(2) Å]. This dimeric assembly, shown in Fig. S2, involves a Cu_2_O_2_ diamond core and results in Cu1–O1 [2.015(2) Å] and Cu1–N1 [2.013(2) Å] bond distances that are slightly longer than those observed in rest of the complexes (Table S4). The solution characterization of Cu3, which includes its NMR spectra and other photophysical data described below, is not at odds with the rest of the complexes and suggests that the dimeric architecture is likely confined to the solid state only.

### DFT calculations

To evaluate the frontier molecular orbitals of Cu1–Cu7, density functional theory (DFT) was carried out at the B3LYP level with LANL2DZ (Cu) and 6-31 + g(d) [C, H, N, O, F] basis sets. Optimized geometries are displayed in Fig. S45, and for Cu1–Cu5 and Cu7, which were characterized crystallographically, their DFT-optimized structures are overlaid with the crystal structures in Fig. S46. In general, there is good agreement between the crystal structures and the DFT-optimized structures, in terms of bond metrics and the relative conformation of the two ligands. In Cu3, the crystal structure is distorted from planarity by virtue of the solid-state dimerization (see above), but the optimized structure of the monomer does exhibit a planar geometry. In most cases, the conformation of the aryl isocyanide relative to the Cu-salicylaldimine core is similar in the crystal structures and optimized structures, with only a slight twist of the isocyanide aryl ring relative to the chelate ring. The exception is Cu7, where the crystal structure has a nearly coplanar arrangement of the Cu-salicylaldimine chelate ring and the central aryl ring, but in the DFT-optimized structure there is a significant twist and almost orthogonal arrangement of the two rings.

**Fig. 2:**
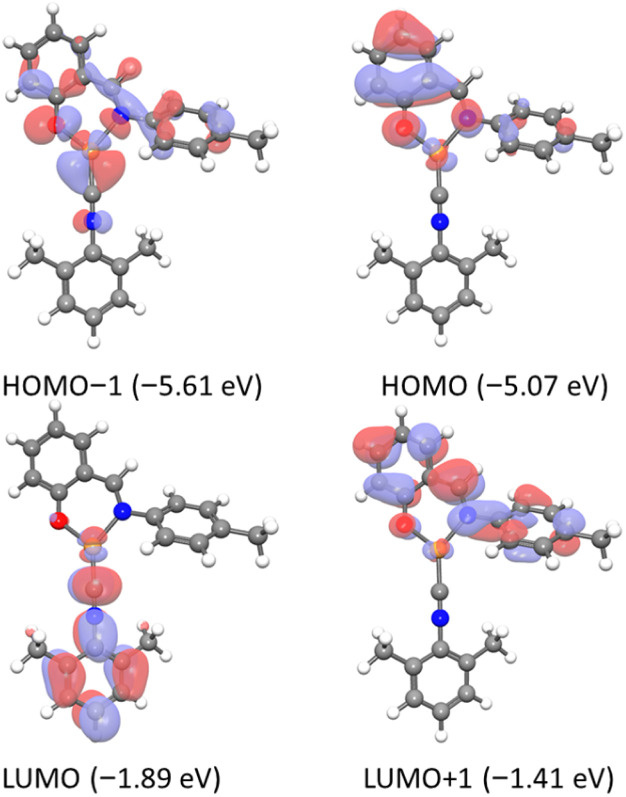
Frontier molecular orbitals of Cu1, computed *via* DFT, using B3LYP/LANL2DZ/6-31 + g(d) level of theory (isovalue 0.08).

All complexes display comparable electronic distributions in their frontier orbitals, consistent with their structural similarities. Fig. 2 displays the frontier orbitals (HOMO−1 to LUMO+1) of Cu1, with selected orbitals for all complexes in Fig. S47, S49–S53, and S55. For Cu1, both the HOMO and HOMO−1 are primarily localized on the salicylaldimine ligand, with only minor contributions from the Cu center (4.1% for HOMO−1 and 3.3% for HOMO). This localization is likely due to the electron-rich nature of the monoanionic N^O chelating ligand. In contrast, the LUMO is largely situated on the aryl isocyanide ligand, with minimal copper character (1.4%), while the LUMO+1 is mainly localized on the salicylaldimine ligand, showing only 0.03% Cu contribution. Similar frontier orbital electron density was computed across the entire series. This electronic structure, in which the HOMO is localized on the anionic chelating L^X ligand the LUMO is on the isocyanide, is broadly similar to previously reported [Cu(NacNac)(CN-Ar)] complexes (NacNac = substituted β-diketiminate), although in the NacNac analogues both the LUMO and LUMO+1 are localized on the isocyanide ligand.^37^ The calculated HOMO–LUMO energy gaps span from 3.17 to 3.50 eV. Most frontier orbital energies are similar, although in Cu2 (*N*-aryl = 2,4-bis(trifluoromethyl)phenyl) and Cu3 (*N*-aryl = mesityl) we observe a significant stabilization of the HOMO that leads to these two complexes having the largest computed HOMO–LUMO gaps.

### Photophysical properties

**Fig. 3:**
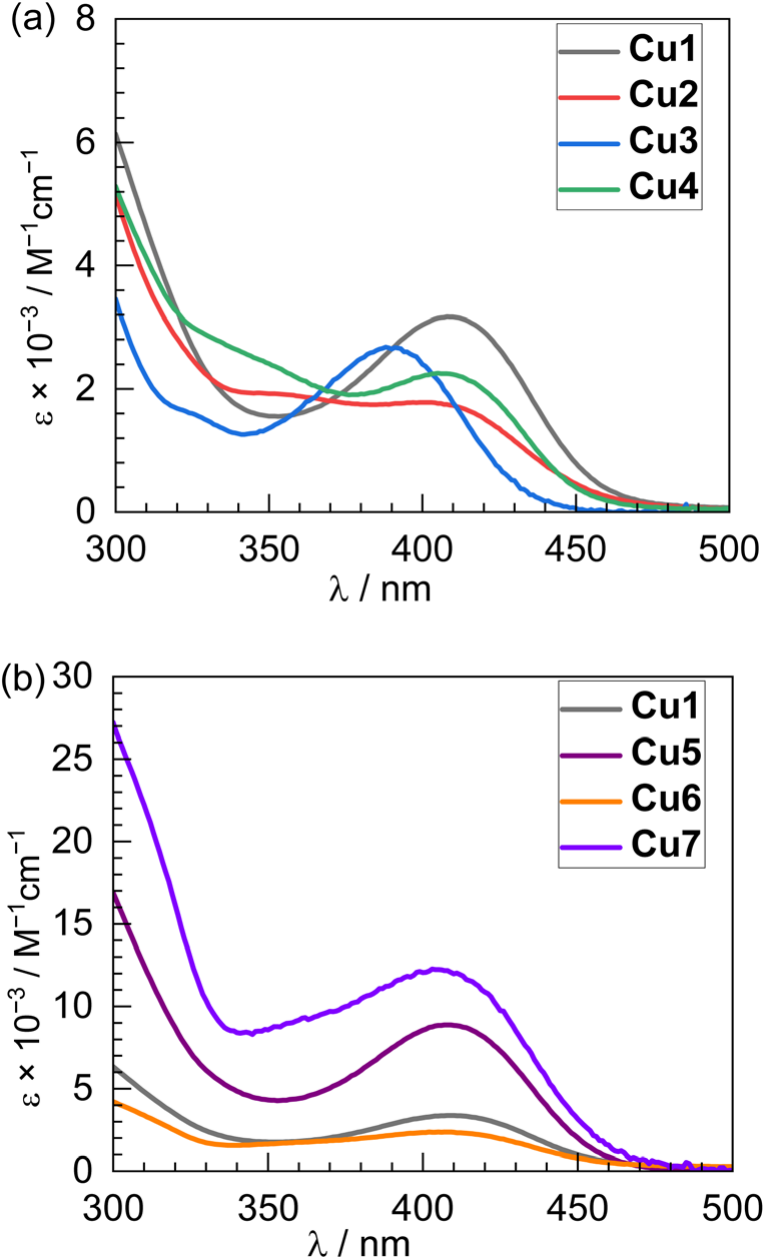
Overlaid UV-vis absorption spectra of (a) Cu1–Cu4; and (b) Cu1 and Cu5–Cu7, recorded in toluene at room temperature.

**Fig. 4:**
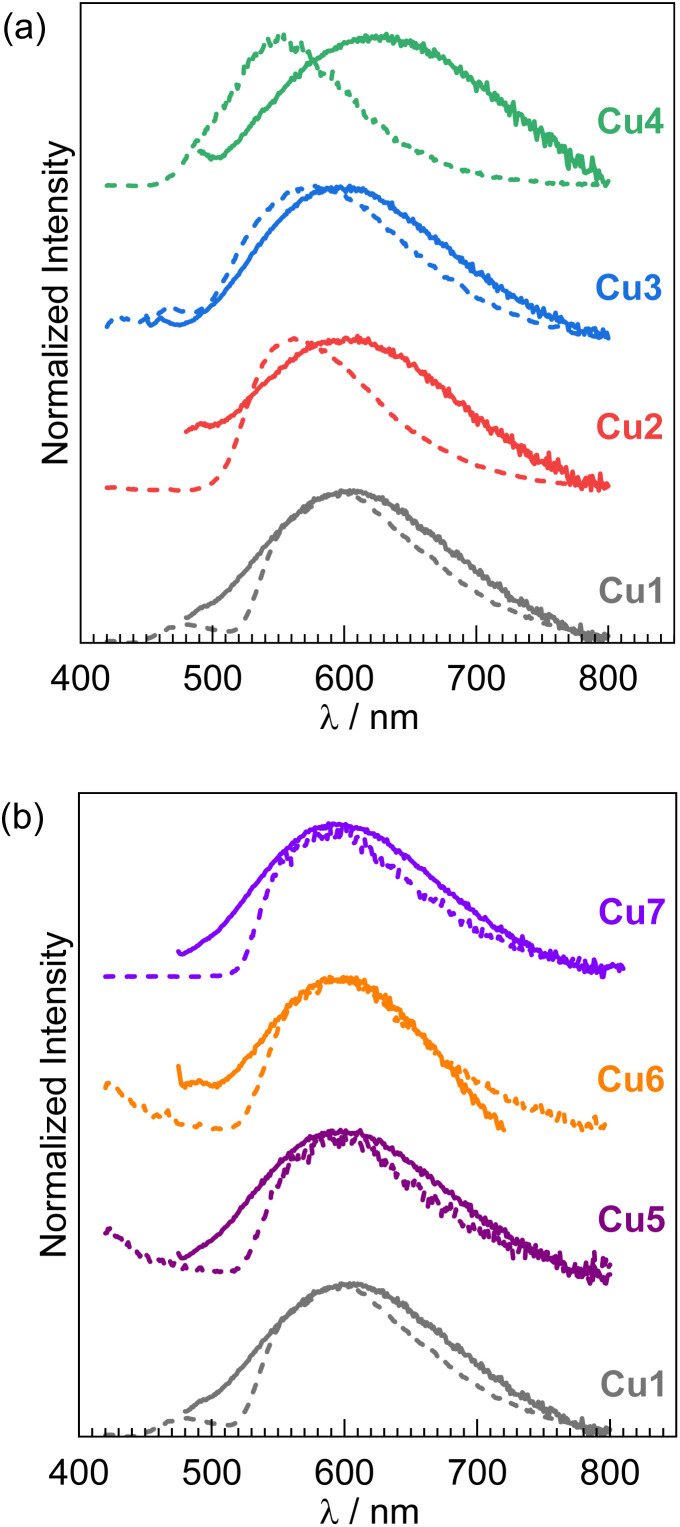
Overlaid photoluminescence spectra (at 298 K and 77 K) of (a) Cu1–Cu4 and (b) Cu1 and Cu5–Cu7, recorded in toluene. Solid lines represent PL spectra at 298 K and dashed lines represent PL spectra at 77 K.

UV-vis absorption and steady-state photoluminescence (PL) spectra of Cu1–Cu7 were recorded in toluene at room temperature (298 K), with PL recorded at low temperature (77 K) as well. The absorption and PL spectra are shown in Fig. 3 and 4, respectively, with complete photophysical data summarized in Table 1. Excitation spectra, overlaid with their corresponding absorption spectra, are provided in the SI (Fig. S3–S9). All complexes display broad absorption bands with maxima at the edge of the visible region (388–408 nm), characteristic of charge-transfer transitions. The position of this band is mildly sensitive to substituents on the salicylaldimine but depends minimally on the isocyanide. As shown in Fig. 3a, Cu1 (4-tolyl substituent) exhibits a broad visible absorption centered at 408 nm. Substitution with CF_3_ groups at the 2- and 4-positions of the *N*-phenyl (Cu2) or the addition of two more methyl groups (Cu3) results in hypsochromic shifts of the absorption maxima to 397 nm and 388 nm, respectively. Cu4, which has an *m*-terphenyl substituent on the salicylaldimine, displays an absorption maximum at 405 nm, comparable to Cu1. Increasing the steric bulk of the isocyanide ligands (Cu1 and Cu5–Cu7) leaves the absorption maximum largely unchanged, with all four complexes exhibiting similar bands centered at 405–408 nm (Fig. 3b).

**Table 1:** Summary of UV-vis absorption and photoluminescence data

	UV-vis absorption, *λ*/nm (*ε* ×10^−3^/M^−1^ cm^−1^)	PL, 298 K, *λ*/nm	PL, 77 K, *λ*/nm	*τ*/ns	*Φ*_PL_ a	*k*_r_/s^−1^	*k*_nr_/s^−1^	*E*_0,0_ b/eV
Cu1	408 (3.4)	604	597	109	1.2 × 10^−3^	1.1 × 10^4^	9.2 × 10^6^	2.1
Cu2	401 (1.9), 356 (2.1, sh)	601	562	70	8 × 10^−4^	1 × 10^4^	1 × 10^7^	2.2
Cu3	388 (2.7)	604	575	28	6 × 10^−4^	2 × 10^4^	4 × 10^7^	2.2
Cu4	405 (2.2)	632	551	22	7 × 10^−4^	3 × 10^4^	5 × 10^7^	2.3
Cu5	408 (8.9)	597	597	147	1.2 × 10^−3^	8.2 × 10^3^	6.8 × 10^6^	2.1
Cu6	405 (2.4)	589	591	375	9.0 × 10^−3^	2.4 × 10^4^	2.6 × 10^6^	2.1
Cu7	406 (12.2)	588	589	329	8.0 × 10^−3^	2.4 × 10^4^	3.0 × 10^6^	2.1

a Determined in toluene, relative to tetraphenylporphyrin (*Φ* = 0.11). The reported values are the averages of three independent experiments conducted on each compound.

b *E*_0,0_ values were calculated from the PL maxima in spectra recorded at 77 K.

To gain deeper insight into the nature of the observed electronic transitions, time-dependent density functional theory (TD-DFT) calculations were performed at the B3LYP/6-31+G(d) level of theory. The key transitions for all complexes are summarized in Tables S7–S13. Given the similar electronic structures and orbital characteristics across the Cu1–Cu7 series, Cu1 was selected as a representative example, and the molecular orbitals involved in its low-energy transitions are depicted in Fig. 2. The low-energy absorption band involves substantial configuration interaction, with four major one-electron transitions as contributors: HOMO−1 → LUMO, HOMO → LUMO, HOMO−1 → LUMO+1, and HOMO → LUMO+1. The LUMO is primarily localized on the aryl isocyanide ligand, indicating that the HOMO−1 → LUMO and HOMO → LUMO transitions are best described to ligand-to-ligand charge transfer (LL′CT) from the electron-rich salicylaldimine to the π-accepting isocyanide. In contrast, the LUMO+1 is localized on the salicylaldimine moiety, such that the transitions that populate this orbital from HOMO or HOMO−1 are assigned as intraligand charge transfer (ILCT) within the salicylaldimine. These characteristics were consistent across all complexes studied. Consequently, the broad absorption bands observed in the visible region arise from a combination of LL′CT and ILCT transitions, and all computed low-energy transitions are near 400 nm, consistent with the experimental spectra. Moreover, the computations confirm experimental observations that indicate that altering the isocyanide ligand has minimal effect on the low-energy absorption transition. Cu1 and Cu4–Cu7 have very similar computed HOMO–LUMO gaps (3.18–3.26 eV), and the computed TD-DFT transitions with the highest oscillator strength in Cu1 (Table S7) and Cu4–Cu7 (Tables S10–S13) occur at nearly identical wavelengths.

Further confirmation of the above assignment comes from natural transition orbital (NTO) analysis of Cu1, performed for the S_0_ → S_3_ excitation (*f* = 0.2689) that is the most intense transition in the calculated spectrum (Fig. S48). The excitation is governed by a single dominant hole–particle pair (*λ*_1_ = 0.93), with only a minor contribution from the second pair (*λ*_2_ = 0.06). In the dominant hole–particle pair, the hole orbital is predominantly localized on the salicylaldimine fragment, whereas the particle orbital is centred on the isocyanide ligand, clearly suggesting ligand-to-ligand charge-transfer (LL′CT) character (Fig. S48a). On the other hand, the minor pair displays the hole orbital on the salicylaldimine moiety and particle orbital is delocalized over both the salicylaldimine and isocyanide ligands, suggesting a minor contribution of intraligand charge transfer (ILCT) (Fig. S48b). Similar results were obtained from the NTO analysis of Cu6, which features a bulky aryl isocyanide ligand, where the dominant pair again indicates substantial LL′CT character (Fig. S54). The consistency of these results further confirms that the low-energy absorption band is primarily attributed to a ligand-to-ligand charge-transfer transition with minor ILCT contribution.

All seven Cu(i) complexes exhibit photoluminescence at both room temperature and 77 K, with large shifts from the absorption maxima consistent with phosphorescence (Fig. 4). Again, Fig. 4 overlays the spectra from the four complexes with variable salicylaldimine ligands in panel (a), with the four complexes with varying isocyanides displayed in panel (b). In toluene at 298 K, Cu1 displays a broad, featureless PL band centered at 604 nm, characteristic of phosphorescence arising from a triplet charge-transfer state (^3^LL′CT/^3^ILCT). Similar profiles are observed for Cu2 and Cu3, with maxima at 601 nm and 604 nm, respectively (Fig. 4a). Notably, Cu4—bearing the most sterically hindered salicylaldimine ligand—exhibits a pronounced red shift, with a PL maximum at 632 nm (Fig. 4a, green trace). Upon cooling to 77 K, all complexes show maxima that are blue-shifted relative to room temperature (Cu1: 597 nm; Cu2: 562 nm; Cu3: 575 nm; Cu4: 551 nm), indicative of inhibited structural relaxation in the frozen matrix and supporting the charge-transfer nature of the emissive state (Fig. 4a). Upon examining the photoluminescence properties of Cu5–Cu7, which incorporate increasingly bulky aryl isocyanide ligands, a progressive blue shift in the emission maximum was observed relative to Cu1 (Fig. 4b). At room temperature, these complexes exhibit broad, featureless phosphorescence bands centered at 597 nm (Cu5), 589 nm (Cu6), and 588 nm (Cu7). The trend indicates that greater steric bulk on the isocyanide ligand leads to a higher-energy emissive state. Notably, low-temperature (77 K) emission spectra for Cu5–Cu7 show minimal spectral shifts compared to those at room temperature, suggesting reduced geometric distortion in the triplet charge-transfer (^3^CT) excited state—likely a result of the structural rigidity imposed by the bulky isocyanides (Fig. 4b).

A particularly significant outcome of this study is that steric modulation of the ligands exerts a pronounced influence on the lifetime of the ^3^CT excited state, as determined by time-correlated single photon counting (TCSPC) in toluene at room temperature. Time-resolved photoluminescence decay profiles for Cu1–Cu7 are shown in Fig. S11–S17. Cu1 exhibits a PL decay lifetime of 109 ns. The additional substituents on the salicylaldimine ligand (Cu2–Cu4), which increase its steric bulk, result in a marked decrease in lifetimes, measured as 70 ns (Cu2), 28 ns (Cu3), and 22 ns (Cu4), respectively. Identifying the 4-tolyl-substituted salicylaldimine as giving the longest excited-state lifetime, Cu5–Cu7 include this same chelating ligand with increasing steric bulk on the isocyanide, which in this case begets a progressive increase in ^3^CT lifetime. Replacing 2,6-dimethylphenyl isocyanide in Cu1 (*τ* = 109 ns) with the bulkier 2,6-diisopropyl isocyanide in Cu5 gives a modestly increased excited-state lifetime of 147 ns. The *m*-terphenyl isocyanides used in Cu6 and Cu7 are significantly more effective, with the lifetime reaching 375 ns in Cu6 and a similar value of 329 ns in Cu7. The *ca.* 3.5-fold increase in lifetime brought on by the *m*-terphenyl isocyanides is smaller than the nearly 30-fold increase observed in previously reported [Cu(NacNac)(CN-Ar)] complexes,^37^ although in absolute terms the maximum lifetime in the present set of complexes (375 ns in Cu6) exceeds that of the previously described NacNac complexes (276 ns).

The photoluminescence quantum yields (*Φ*_PL_), while low across the series (6 × 10^−4^ to 9.0 × 10^−3^), exhibit a clear correlation with the steric profiles of the ligands, mirroring the trends observed in excited-state lifetimes. The PL quantum yield in Cu1 is 1.2 × 10^−3^, which decreases when the substituents are altered on the salicylaldimine ligand: *Φ*_PL_ = 8 × 10^−4^ in Cu2, 6 × 10^−4^ in Cu3, and 7 × 10^−4^ in Cu4. This trend is consistent with the observed reduction in ^3^CT lifetimes across the series and is driven by larger nonradiative rate constants (*k*_nr_) in Cu2–Cu4. On the other hand, when varying the isocyanide, Cu5 displays a photoluminescence quantum yield (*Φ*_PL_ = 1.2 × 10^−3^) identical to Cu1, which increases significantly in *m*-terphenyl isocyanide complexes Cu6 (*Φ*_PL_ = 9.0 × 10^−3^) and Cu7 (*Φ*_PL_ = 8.0 × 10^−3^). The calculated radiative rate constant (*k*_r_) for Cu5 is 8.2 × 10^3^ s^−1^, slightly lower than that of Cu1 (1.1 × 10^4^ s^−1^). The differences in quantum yield and lifetime across Cu1 and Cu5–Cu7 are brought on primarily by substantial decreases in *k*_nr_ in the analogues with the bulkier isocyanides. Taken together, these findings underscore that steric augmentation of the acceptor ligand (isocyanide) in this class of complexes is effective at enhancing excited-state lifetime and quantum yield, whereas similar steric modifications to the donor ligand (salicylaldimine) are detrimental. We conducted PL measurements on Cu1 in poly(methyl methacrylate) (PMMA) film at 2 wt%. As shown in Fig. S10, luminescence is observed in the film, but it is too weak to determine an accurate absolute quantum yield using an integrating sphere.

### Electrochemical properties

**Fig. 5:**
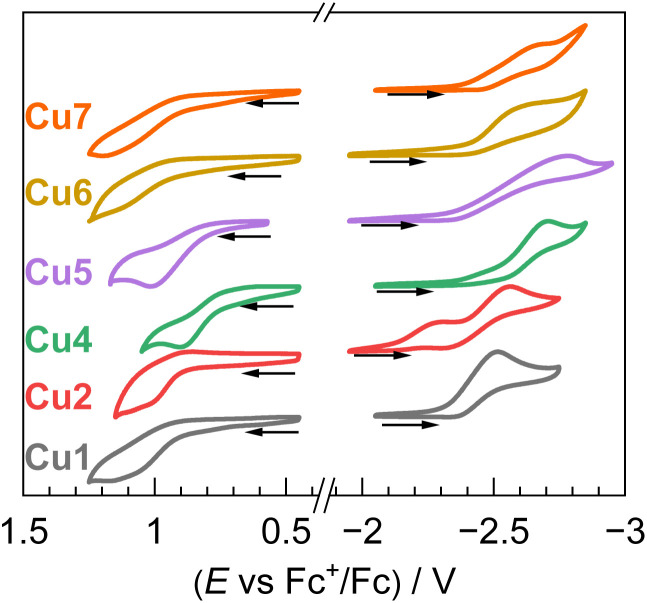
Overlaid cyclic voltammograms of Cu1–Cu7 (recorded in THF with 0.1 M NBu_4_PF_6_ electrolyte, glassy carbon working electrode, platinum wire counter electrode, silver wire pseudo-reference electrode, scan rate 0.1 V s; reported with internal standard reference of ferrocenium/ferrocene redox couple).

The electrochemical behavior of Cu1–Cu7 was investigated by cyclic voltammetry (CV), and the corresponding voltammograms are shown in Fig. 5. Measurements were carried out in THF using 0.1 M [*n*Bu_4_N]PF_6_ as the supporting electrolyte, with a glassy carbon working electrode, a platinum wire counter electrode, and a silver wire pseudo-reference electrode. Redox potentials are referenced against the ferrocenium/ferrocene (Fc^+^/Fc^0^) couple, used as an internal reference. A summary of the electrochemical data is provided in Table 2. Cyclic voltammetry of Cu3 could not be recorded due to its poor solubility. Across the series of complexes, irreversible oxidation waves were observed, with half-peak potentials (*E*^ox^) ranging from 0.82 to 1.03 V. The observed oxidation waves of these Cu(i) complexes can be formally assigned to Cu(ii)/Cu(i) redox couples, but the DFT calculations described above reveal substantial HOMO electron density localized on the salicylaldimine ligand framework, indicating significant redox activity of the chelating ligand. A gradual decrease in oxidation potential from Cu1 to Cu2 and Cu4 was noted, indicating that steric augmentation of the salicylaldimine ligand incurs a slight destabilization of the HOMO. In contrast, Cu5–Cu7, which share the identical 4-tolyl substituted salicylaldimine ligand with Cu1, exhibit nearly identical *E*^ox^ values, despite varying isocyanide substituents. Irreversible reduction waves are also observed, with half-peak potentials spanning from −2.22 to −2.63 V. The LUMO is primarily a π* orbital comprised of the C≡N triple bond and the central arene of the isocyanide, and as such *E*^red^ values are nearly identical across most of the series. The one exception is Cu2, where the low-lying virtual orbitals involve significant delocalization onto the bis(trifluoromethyl)phenyl ring, leading to a substantial anodic shift of the first reduction potential (*E*^red^ = −2.22 V). From these data, the excited-state potentials (*E^ox^, formally a [Cu]^+^/*[Cu]^0^ couple) were estimated to assess the photoredox capabilities of each complex. These values, ranging from −1.1 to −1.5 V, reflect moderate excited-state reducing power. Notably, Cu4 displayed the most negative *E^ox^ value, implying it is the strongest photo-reductant in the present series of Cu(i) photosensitizers. Although Cu1 and Cu5–Cu7 exhibit significantly longer excited-state lifetimes, their excited-state redox potentials *E^ox^ remain relatively modest, all estimated at −1.1 V.

**Table 2:** Summary of electrochemical data of Cu1–Cu7

	*E* ^ox^ a/V ([Cu]^+^/[Cu]^0^)	*E* ^red^ a/V ([Cu]^0^/[Cu]^−^)	*E^ox^^b^/V ([Cu]^+^/*[Cu]^0^)
Cu1	1.01	−2.43	−1.1
Cu2	0.96	−2.22	−1.2
Cu4	0.82	−2.63	−1.5
Cu5	1.00	−2.56	−1.1
Cu6	1.01	−2.55	−1.1
Cu7	1.03	−2.57	−1.1

a CV waves are irreversible, half-peak potentials are given.^38^

b Estimated as *E*^ox^ − *E*_0,0_ (see Table 1 for *E*_0,0_ values).

### Photocatalytic activity

Considering the relatively mild excited-state reducing power of Cu1–Cu7 (*E^ox^ = −1.1 to −1.5 V), it appears that these complexes may have limited efficacy as photoreductants for activating challenging substrates that require more negative reduction potentials. Therefore, to evaluate the photocatalytic utility of these newly developed Cu(i)-based photosensitizers, we explored their performance in a prototypical triplet–triplet energy transfer (TTET) photocatalytic transformation, the isomerization of (*E*)-stilbene. This *E*/*Z* photoisomerization of stilbene is a well-established benchmark for evaluating TTET photosensitizers, as the mechanism is well understood and the product distribution provides a clear readout of energy-transfer efficiency.^39,40^ In addition, *E*/*Z* isomerization of stilbene represents an example of an energy-storing photoreaction, with the *Z* isomer *ca.* 4 kcal mol^−1^ less stable than the *E* isomer.^41^ In this process, the Cu(i) photosensitizer in its ^3^CT state transfers energy to (*E*)-stilbene, generating a triplet stilbene diradical that undergoes conformational twisting and intersystem crossing to yield the (*Z*)-isomer.^40,42^ A longer excited-state lifetime enhances this process by increasing the probability of productive TTET before nonradiative decay occurs, *i.e.*, by improving the quantum yield of TTET, which allows the isomerization to progress more quickly.^40^ Given the variable excited-state lifetimes of Cu1–Cu7 (ranging from 22 to 375 ns), this reaction served as a suitable benchmark to correlate photocatalytic efficiency with excited-state decay dynamics.

**Scheme 2:**
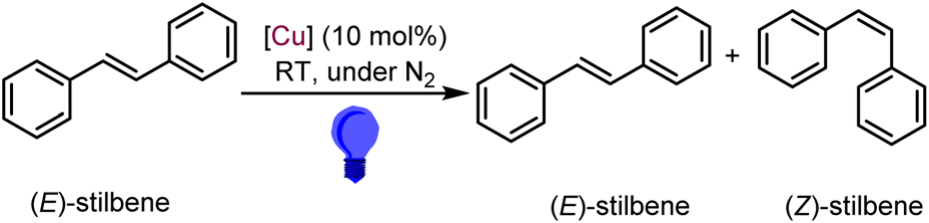
Photocatalytic *E/Z* isomerization of (*E*)-stilbene using Cu catalysts Cu1–Cu7.

As shown in Scheme 2, the isomerization reactions were conducted under blue-light irradiation (430–500 nm range, 460 nm maximum) in C_6_D_6_, with (*E*)-stilbene as the substrate, 1,3,5-trimethoxybenzene as the internal integration standard, and 10 mol% of Cu complex as catalyst. The reaction progress was monitored by ^1^H NMR spectroscopy at regular intervals, and the corresponding spectra are provided in Fig. S19–S25. The detailed catalytic outcome is presented in Table S5 and briefly summarized in Table 3. The complexes in this study all promote photoisomerization of (*E*)-stilbene and reach similar photostationary *E* : *Z* ratios, but the rate at which they approach that photostationary state varies. For all seven complexes, reactions were initially monitored over the course of 24 h.

**Table 3:** Summary of results for photocatalytic *E/Z* isomerization of (*E*)-stilbene

Entry	Catalyst	Time/h	*E* : *Z*
1	Cu1	1	11 : 89
2	Cu1	24	10 : 90
3	Cu2	1	75 : 25
4	Cu2	24	11 : 89
5	Cu3	1	47 : 53
6	Cu3	24	17 : 83
7	Cu4	1	36 : 64
8	Cu4	24	17 : 83
9	Cu5	1	14 : 86
10	Cu5	24	14 : 86
11	Cu6	1	12 : 88
12	Cu6	24	11 : 89
13	Cu7	1	21 : 79
14	Cu7	24	11 : 89
15^a^	—	24	100 : 0
16^b^	Cu1 (in dark)	24	100 : 0

a Catalytic reactions performed in the absence of any copper photosensitizers for 24 h.

b Catalytic reactions performed in dark (without blue LED irradiation) for 24 h.

When Cu1 was used as catalyst, the *E* : *Z* photostationary state (10 : 90) was achieved within 1 hour, with minimal progression of the reaction thereafter until 24 hours (Table 3, entries 1 and 2). In contrast, catalysts Cu2–Cu4, which exhibit shorter excited-state lifetimes, displayed slower kinetics; *E* : *Z* ratios after 1 hour were recorded as 88 : 12 (Cu2), 74 : 26 (Cu3), and 63 : 37 (Cu4), although all eventually reached nearly similar photostationary states after 24 hours (Table 3, entries 3–8). These results highlight the critical role of excited-state lifetime in governing TTET reactivity. Further, catalysts Cu5 and Cu6, bearing more sterically hindered isocyanide ligands and longer excited-state lifetimes, facilitate rapid isomerization, achieving the photostationary state within 1 hour (Table 3, entries 9–12). Cu7, while still effective, showed some progression between 1 h (Table 3, entry 13) and 2 h (Table S5) before reaching a comparable endpoint (Table 3, entry 14). As the ^3^CT *E*_0,0_ energies of all seven complexes are quite similar (Table 1), resulting in all experiments reaching a similar photostationary state, the observed variations in catalytic rate are best attributed to differences in ^3^CT excited-state lifetimes. Control experiments confirmed that no isomerization occurred in the absence of light or Cu(i) photosensitizers (Table 3, entries 15 and 16), reinforcing the photosensitized TTET mechanism.

For the four complexes that give the fastest rates of photoisomerization (Cu1, Cu5, Cu6, and Cu7), additional insight into their TTET reactivity was gained from experiments monitored on a shorter 1 hour timescale. Although all four complexes reach or nearly reach the final photostationary state within 1 h, monitoring the reactions at short time intervals reveals distinctly different kinetics (Table S6 and Fig. S26–S29). Cu1 (*τ* = 109 ns) and Cu5 (*τ* = 147 ns) showed comparable initial rates of photoisomerization, with Cu1 gradually approaching its photostationary state over 1 h, and Cu5 reaching it within 35 minutes. The reaction with Cu6 (*τ* = 375 ns) progresses even faster over the first 20 minutes, highlighting the role an extended lifetime can play in accelerating TTET reactivity, with the photostationary state (*E* : *Z* = 11 : 89) again being reached within 35 min. However, despite its long lifetime (*τ* = 329 ns), Cu7 promoted comparatively slower stilbene isomerization than Cu1, Cu5, and Cu6, indicating there isn't a universal relationship between excited-state lifetime and photocatalytic activity in this set of complexes.

## Conclusions

In summary, we have developed and systematically investigated a new class of Cu(i) photosensitizers featuring salicylaldimine and aryl isocyanide ligands, where steric modulation at both donor and acceptor sites modestly shifts excited-state energies and redox potentials but has substantial effects on excited-state decay dynamics. Detailed spectroscopic and computational analyses reveal that the present series of Cu(i) complexes exhibit prominent charge-transfer transitions comprising both ligand-to-ligand charge transfer (LL′CT) from salicylaldimine to isocyanide and intra-ligand charge transfer (ILCT) transitions localized within the salicylaldimine framework. Whereas increased steric encumbrance on the salicylaldimine ligands increases the nonradiative decay rate and results in diminished photoluminescence quantum yields and shortened excited-state lifetimes, increasing the steric bulk on the isocyanide ligands effectively suppresses nonradiative deactivation and prolongs the excited-state lifetimes. These findings are corroborated by photocatalytic TTET reactivity, where excited-state lifetimes directly correlate with catalytic performance. The ability of these Cu(i)-complexes to mediate efficient triplet–triplet energy transfer in stilbene isomerization reactions suggests they may hold promise in other more challenging triplet-sensitized reactions that could be involved triplet–triplet annihilation and upconversion processes or solar energy conversion.

Steric protection prolongs the excited-state lifetimes, but the photoluminescence quantum yields are modest across the series, due to a *ca.* 2–3 order of magnitude difference between the radiative (*k*_r_) and nonradiative (*k*_nr_) rate constants. Although the present work does not reveal any clear path to address this limitation, we do note that the *k*_r_ values observed here (Table 1) are nearly an order of magnitude smaller than those observed in copper(i) β-diketiminate isocyanide complexes, suggesting that slow radiative rates may be a shortcoming of mixed-donor *N*,*O* chelates.

This work furthers our efforts with an emerging class of heteroleptic copper(i) chromophores that partner electron-rich anionic chelating ligands with neutral π-acceptors, spatially separating the frontier orbitals. The key outcome outlined here is that electronically neutral but sterically differentiating substituents can play large roles in determining the excited-state dynamics and photocatalytic reactivity. An important conceptual advance over our previous work is demonstrating that asymmetric, mixed-donor *N*,*O* chelating ligands, function effectively in this emerging class of heteroleptic photosensitizers, motivating pursuit of other classes of mixed-donor chelating ligands as a means of controlling excited-state energies, lifetimes, and redox potentials.

## Author contributions

Soumi Chakraborty: formal analysis, investigation, validation, visualization, writing – original draft, writing – review & editing. Kianna Agyekum: formal analysis. Dooyoung Kim: formal analysis. Thomas S. Teets: conceptualization, funding acquisition, project administration, visualization, writing–review & editing.

## Conflicts of interest

There are no conflicts to declare.

## Supplementary Material

SC-OLF-D5SC07286G-s001

SC-OLF-D5SC07286G-s002

SC-OLF-D5SC07286G-s003

SC-OLF-D5SC07286G-s004

SC-OLF-D5SC07286G-s005

SC-OLF-D5SC07286G-s006

SC-OLF-D5SC07286G-s007

SC-OLF-D5SC07286G-s008

SC-OLF-D5SC07286G-s009

## Data Availability

CCDC 2453584 (Cu1), 2453585 (Cu2), 2453586 (Cu3), 2453587 (Cu4), 2453588 (Cu5) and 2453589 (Cu7) contain the supplementary crystallographic data for this paper.^43^ The data supporting this article have been included as part of the supplementary information (SI). Supplementary information: Experimental details, X-ray crystallography summary tables, additional X-ray crystal structure figures, additional photophysical data, NMR spectra, and details of DFT calculations. See DOI: https://doi.org/10.1039/d5sc07286g.
