# Supplementary material for: Ligand substituents modulate excited-state lifetime and energy-transfer reactivity in Cu(i) photosensitizers supported by salicylaldimine and isocyanide ligands
Source: Chem Sci. 2025 Oct 27;16(47):22527–35. doi: 10.1039/d5sc07286g (PMC12557405; doi:10.1039/d5sc07286g)
Supplement: SC-016-D5SC07286G-s001 [file SC-016-D5SC07286G-s001.pdf]

Supplementary Information (SI) for Chemical Science.
This journal is © The Royal Society of Chemistry 2025

Supporting Information for

Ligand Substituents Modulate Excited-State Lifetime and Energy-Transfer Reactivity in Cu(I) Photosensitizers Supported by Salicylaldimine and Isocyanide Ligands

Soumi Chakraborty, Kianna Agyekum, Dooyoung Kim, and Thomas S. Teets*

Department of Chemistry, University of Houston, Houston, Texas 77204, United States

*E-mail: tteets@uh.edu

**Contents**	**Pages**
Materials and Methods	S2
Synthesis	S3–S6
X-ray Crystallographic Details and Additional Figures	S7–S11
Additional Photophysical Data	S12–S20
Photostability test of **Cu2**	S21
Photocatalysis Experimental Details and Results	S22–S23
^{1}H NMR Spectra Monitoring Photocatalytic Reactions	S24–S29
^{1}H and ^{13}C{^{1}H} NMR Spectra of Cu Complexes	S30–S37
DFT Calculations	S38–S48
References	S49

Materials and methods.

All reactions were performed in a nitrogen-filled glovebox (at <1 ppm of oxygen and <1 ppm of H_2O). Solvents for synthesis, photophysical, and electrochemical measurements were either dried and deoxygenated in a solvent purification system and stored over 3Å molecular sieves or were obtained commercially at anhydrous grade and used directly. NMR solvents were stored inside the glovebox over 3Å molecular sieves. ^{1}H, ^{13}C{^{1}H} and ^{19}F NMR spectra were recorded on a JEOL ECA-500 NMR spectrometer at room temperature. Stock solutions for photophysical measurements were prepared inside a nitrogen-filled glovebox using anhydrous solvents. UV–vis absorption spectra were recorded in anhydrous solvent in a screw-capped quartz cuvettes using an Agilent Cary 8454 UV–vis spectrometer. Steady-state photoluminescence and excitation spectra were recorded using a Horiba FluoroMax-4 spectrofluorometer. The data are corrected for detector response and excitation intensity. Low-temperature photoluminescence measurements were performed in a custom quartz EPR tube with high-vacuum valve, which was immersed in a sample holder consisting of a finger Dewar filled with liquid nitrogen to attain a temperature of 77 K. Solution-state photoluminescence measurements were performed in quartz cuvettes with 1.0 cm path length, using solutions with concentrations adjusted to give absorbance values of 0.05–0.2 at the excitation wavelength.

Photoluminescence quantum yields were determined relative to a standard of tetraphenylporphyrin (TPP) in toluene which has a reported fluorescence quantum yield of 0.11.[1,2] For the standard and each sample, five optically dilute samples of varying were measured, prepared by sequential addition of a stock solution to toluene in a screw-capped cuvette. The absorbance at the excitation wavelength was <0.1 for each measurement. UV–vis absorption spectra and a PL spectrum was recorded at each concentration, ensuring identical slit widths for each measurement. The PL spectrum was integrated using OriginPro software, and for the copper complex and the standard the integrated PL was plotted vs. the absorbance at the excitation wavelength, which for optically dilute samples yields a straight line. The slope of each line was determined, and by using Equation S1 below the quantum yield was determined: Φ_x is the PL quantum yield of the copper complex and Φ_{st} is the quantum yield of the standard. A correction for solvent index of refraction was not needed, since toluene was used as the solvent for both the standard and the copper complex.

$$\Phi_{\mathrm{X}} = \Phi_{\mathrm{ST}} \frac{slope_{\mathrm{X}}}{slope_{\mathrm{ST}}} \tag{S1}$$

Photoluminescence lifetimes of the copper complexes were measured on a Horiba DeltaFlex Lifetime system using a 390 nm pulsed diode excitation source. Cyclic voltammetry (CV) measurements were performed with a CH instrument 602E potentiostat interfaced with a nitrogen-filled glovebox via wire feedthroughs. Tetrabutylammonium hexafluorophosphate (NBu_4PF_6) was used as the supporting electrolyte (0.1 M solution in THF), glassy carbon (3 mm diameter) was used as the working electrode, Pt wire was the counter electrode, and a silver wire immersed in 0.1 M NBu_4PF_6 THF solution was used as a pseudo-reference electrode. All measurements were referenced to an internal standard ferrocene. Starting materials and reagents were obtained from commercial sources unless otherwise stated. Aryl isocyanides are synthesized following literature procedures.[3]

Synthesis

General Procedure for preparation of imine ligands (L1-4).

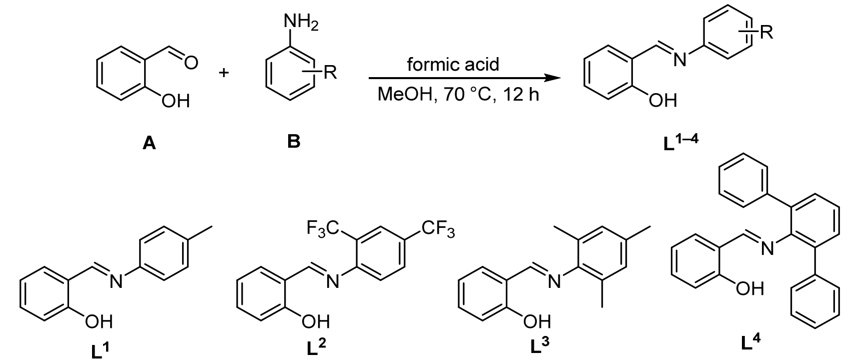

Imine ligands **L**$^{1–4}$ were prepared following a modified literature procedure.[4] In an oven dried 100 mL round-bottom flask equipped with a stir bar, substituted aniline (**B**, 5 mmol) was added followed by methanol as the solvent. The mixture was stirred vigorously for 5 minutes. In a separate flask, salicylaldehyde (**A**, 5 mmol) was dissolved in methanol, and this methanolic solution was added dropwise to the reaction flask. Thereafter a few drops of formic acid were added to it and the reaction mixture was allowed to stir under reflux at 70 °C for 12 hours. After completion, the reaction mixture was concentrated to dryness using a rotary evaporator. For **L**^{1}, the crude product was purified by recrystallization from hot methanolic solution (yellow needle-shaped crystals obtained in 70% yield). For **L**$^{2–4}$, the crude product was purified by column chromatography over silica column with 5–8% ethyl acetate-hexane mixture (yellow solid obtained in 60–65% yield).

General procedure for preparation of L^n-Na salts.

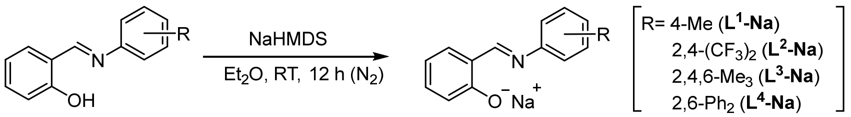

In a nitrogen filled glovebox, the neutral **L**^{n} proligand (1 mmol) was added to a 20 mL vial equipped with a stir bar. NaHMDS (1 mmol) was added to the reaction pot followed by the addition of diethyl ether (5 mL). The reaction mixture was stirred at room temperature for 12 hours. After completion, a yellowish precipitate was observed. The supernatant was decanted and the precipitate was washed repeatedly with diethyl ether and finally dried under vacuum to obtain solid yellow product.

General procedure for preparation of Cu complexes (Cu1–7).

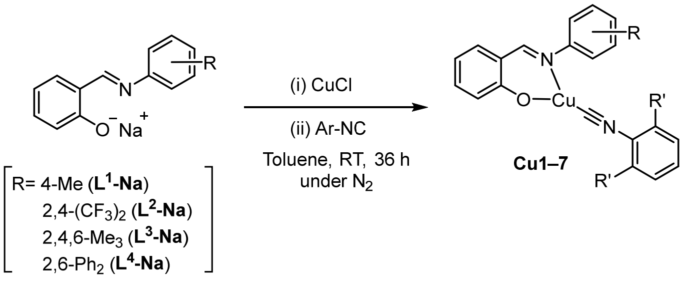

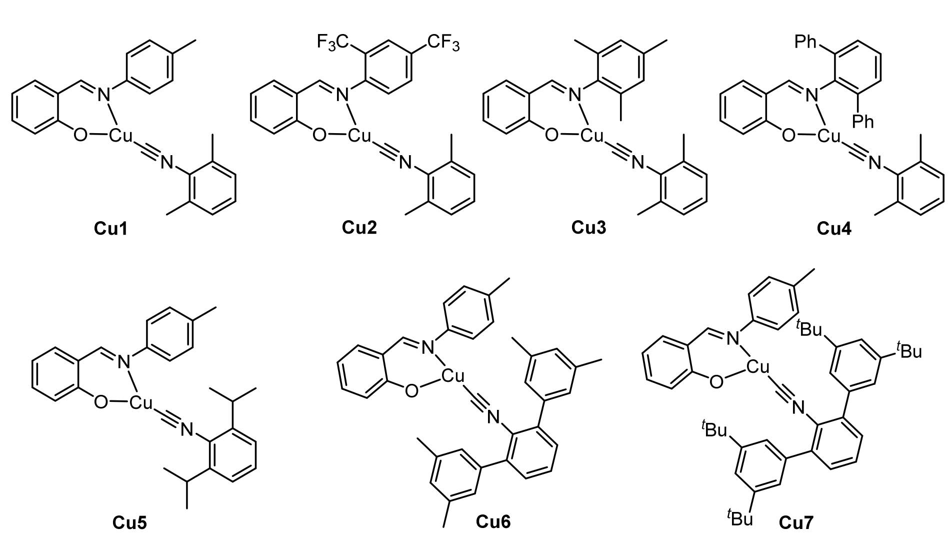

In a nitrogen-filled glovebox, **L**^{n}-Na (0.1 mmol) was added to a 20 mL vial equipped with a stir bar. CuCl (0.1 mmol) was added to it followed by anhydrous toluene (10 mL). The reaction mixture was stirred for 3 hours at room temperature. In a separate vial, aryl isocyanide (0.7 equiv., 0.07 mmol) was dissolved in toluene, and this solution was added dropwise to the reaction vial containing the **L**^{n} ligand and CuCl. The reaction mixture was allowed to stir for 36 hours at room temperature. After completion, the solvent was evaporated under vacuum, followed by a two-step extraction of the compound into pentane. The crude product was purified by recrystallization or reprecipitation from concentrated pentane solution kept inside the glovebox freezer for 48 hours. The Cu complexes were characterized by ^{1}H, ^{13}C{^{1}H}, and ^{19}F (**Cu2**) NMR spectroscopy and X-ray crystallography.

Synthesis of Cu1: Cu1 was prepared by using **L^{1}-Na** (0.20 mmol, 47 mg), CuCl (0.20 mmol, 20 mg), and 2,6-dimethylphenyl isocyanide (0.14 mmol, 18 mg) following the general synthetic procedure. The crude compound was purified by recrystallization from pentane. Yield: 50 mg, 62%. Yellow needle-shaped single crystals of this compound were grown from a pentane solution inside the glovebox freezer. ^{1}H NMR (500 MHz, C_6D_6) δ (ppm): 8.22 (s, 1H), 7.50 (d, J = 8.6 Hz, 1H), 7.36 (ddd, J_1 = 8.6 Hz, J_2 = 6.8 Hz, J_3 = 1.9 Hz, 1H), 7.14–7.09 (m, 3H), 6.96 (d, J = 8.0 Hz, 2H), 6.69 (t, J = 7.6 HZ, 1H), 6.63 (t, J = 7.3 Hz, 1H), 6.50 (d, J = 7.6 Hz, 2H), 2.14 (s, 3H), 1.89 (s, 6H). ^{13}C{^{1}H} NMR (126 MHz, C_6D_6) δ (ppm): 173.0, 166.4, 152.0, 136.5, 135.3, 134.9, 134.8, 129.8, 128.8, 128.3, 127.7, 124.8, 122.6, 119.7, 113.3, 20.8, 18.4. The isocyanide (<u>C</u>≡N) ^{13}C resonance was not located.

Synthesis of Cu2: Cu2 was prepared by using **L^{2}-Na** (0.2 mmol, 71 mg), CuCl (0.2 mmol, 20 mg), and 2,6-dimethylphenyl isocyanide (0.14 mmol, 18 mg) following the general synthetic procedure. The crude compound was purified by recrystallization from pentane. Yield: 50 mg, 47%. Yellow plate-shaped single crystals of this compound were grown from a pentane solution inside the glovebox freezer. ^{1}H NMR (500 MHz, C_6D_6) δ (ppm): 7.83 (s, 1H), 7.78 (s, 1H), 7.43 (d, J = 8.7 Hz, 1H), 7.33 (ddd, J_1 = 8.7 Hz, J_2 = 6.6 Hz, J_3 = 1.9 Hz, 1H), 7.21 (dd, J_1 = 8.3 Hz, J_2 = 2.1 Hz, 1H), 7.07 (dd, J_1 = 7.9 Hz, J_2 = 1.9 Hz, 1H), 6.62 (t, J = 7.6 Hz, 1H), 6.59 (t, J = 7.3 Hz, 1H), 6.44–6.40 (m, 3H), 1.69 (s, 6H). ^{13}C{^{1}H} NMR (126 MHz, C_6D_6) δ (ppm): 173.6, 168.3, 155.9, 136.5, 136.4, 136.2, 135.1, 129.9,

129.1, 128.3, 125.8, 125.2, 124.7, 124.1, 123.5, 123.0, 122.5, 118.4, 113.9, 17.9. The isocyanide ($\underline{C}$≡N) ^{13}C resonance was not located and the CF_3 quartets were not clearly resolved. ^{19}F NMR (565 MHz, C_6D_6) δ (ppm): −57.95 (s, 3F), −61.97 (s, 3F).

Synthesis of Cu3: Cu3 was prepared by using **L^3-Na** (0.2 mmol, 52 mg), CuCl (0.2 mmol, 20 mg), and 2,6-dimethylphenyl isocyanide (0.14 mmol, 18 mg) following the general synthetic procedure. The crude compound was purified by reprecipitation from pentane. Yield: 35 mg, 40%. Yellow plate-shaped single crystals of this compound were grown from a pentane solution inside the glovebox freezer. ^{1}H NMR (500 MHz, C_6D_6) δ (ppm): 7.85 (s, 1H), 7.54 (d, *J* = 8.6 Hz, 1H), 7.39–7.34 (m, 1H), 7.03 (d, *J* = 7.6 Hz, 1H), 6.77 (s, 2H), 6.62 (q, *J* = 7.3 Hz, 2H), 6.42 (d, *J* = 7.6 Hz, 2H), 2.16 (s, 6H), 2.14 (s, 3H), 1.68 (s, 6H). ^{13}C{^{1}H} NMR (126 MHz, C_6D_6) δ (ppm): 173.3, 169.1, 150.4, 136.3, 135.1, 134.8, 133.4, 129.21, 129.16, 128.6, 127.7, 124.9, 119.5, 112.6, 20.8, 18.9, 18.4. The isocyanide ($\underline{C}$≡N) ^{13}C resonance was not located, and one missing aromatic peak was presumably obscured by the C_6D_6 solvent peak.

Synthesis of Cu4: Cu4 was prepared by using **L^4-Na** (0.15 mmol, 56 mg), CuCl (0.15 mmol, 15 mg), and 2,6-dimethylphenyl isocyanide (0.11 mmol, 15 mg) following the general synthetic procedure. The crude compound was purified by recrystallization from pentane. Yield: 31 mg, 38%. Yellow rock-shaped single crystals of this compound were grown from a pentane solution inside the glovebox freezer. ^{1}H NMR (500 MHz, C_6D_6) δ (ppm): 7.83 (s, 1H), 7.49–7.44 (m, 4H), 7.35 (d, *J* = 7.7 Hz, 2H), 7.29 (d, *J* = 7.4 Hz, 1H), 7.10 (t, *J* = 7.7 Hz, 1H), 7.03 (t, *J* = 7.5 Hz, 4H), 6.98–6.92 (m, 2H), 6.72–6.65 (m, 2H), 6.50 (d, *J* = 7.6 Hz, 2H), 6.38 (ddd, J_1 = 7.9 Hz, J_2 = 6.7 Hz, J_3 = 1.2 Hz, 1H), 1.78 (s, 6H). ^{13}C{^{1}H} NMR (126 MHz, C_6D_6) δ (ppm): 172.8, 171.8, 149.6, 140.1, 136.0, 135.7, 135.1, 135.0, 130.7, 130.6, 129.3, 128.7, 128.6, 127.7, 127.1, 125.6, 125.4, 124.6, 119.0, 113.0, 18.4.

Synthesis of Cu5: Cu5 was prepared by using **L^1-Na** (0.24 mmol, 50 mg), CuCl (0.24 mmol, 20 mg), and 2,6-diisopropylphenyl isocyanide (0.17 mmol, 30 mg) following the general synthetic procedure. The crude compound was purified by reprecipitation from pentane. Yield: 38 mg, 34%. Yellow rock-shaped single crystals of this compound were grown from a pentane solution at room temperature. ^{1}H NMR (500 MHz, C_6D_6) δ (ppm): 8.19 (s, 1H), 7.51 (d, *J* = 8.6 Hz, 1H), 7.35 (t, *J* = 6.6 Hz, 1H), 7.13–7.06 (m, 3H), 6.96–6.91 (m, 3H), 6.76 (d, *J* = 7.7 Hz, 2H), 6.62 (t, *J* = 7.2 Hz, 1H), 3.06 (sept, *J* = 6.9 Hz, 2H), 2.14 (s, 3H), 0.96 (d, *J* = 6.7 Hz, 12H). ^{13}C{^{1}H} NMR (126 MHz, C_6D_6) δ (ppm): 172.9, 166.5, 152.2, 145.5, 136.5, 134.9, 134.7, 129.8, 129.7, 128.3, 127.5, 124.8, 123.5, 122.5, 119.6, 113.3, 29.9, 22.3, 20.8.

Synthesis of Cu6: Cu6 was prepared by using **L^1-Na** (0.12 mmol, 25 mg), CuCl (0.12 mmol, 10 mg), and 2,6(3,5-dimethylphenyl)phenyl isocyanide (0.080 mmol, 25 mg) following the general synthetic procedure. The crude compound was purified by reprecipitation from pentane. Yield: 18 mg, 26%. ^{1}H NMR (500 MHz, C_6D_6) δ (ppm): 7.86 (s, 1H), 7.25 (d, *J* = 8.6 Hz, 1H), 7.20 (ddd, J_1 = 8.6 Hz, J_2 = 6.5 Hz, J_3 = 1.8 Hz, 1H), 6.93–6.88 (m, 6H), 6.87–6.81 (m, 2H), 6.68–6.65 (m, 4H), 6.59 (d, *J* = 8.3 Hz, 2H), 6.43 (ddd, J_1 = 7.9 Hz, J_2 = 6.6 Hz, J_3 = 1.3 Hz, 1H), 2.07 (s, 12H), 1.98 (s, 3H). ^{13}C{^{1}H} NMR (126 MHz, C_6D_6) δ (ppm): 172.8, 165.9, 151.7, 140.6, 138.4, 137.4, 136.4, 134.6, 134.4, 130.5, 129.6, 129.3, 129.2, 127.1, 126.9, 124.6, 122.3, 119.5, 113.1, 21.3, 20.8. The isocyanide ($\underline{C}$≡N) ^{13}C resonance was not located.

Synthesis of Cu7: Cu7 was prepared by using **L^1-Na** (0.12 mmol, 25 mg), CuCl (0.12 mmol, 10 mg) and 2,6(3,5-di-*tert*-butylphenyl)phenyl isocyanide (0.079 mmol, 38 mg) following the general synthetic procedure. The crude compound was purified by recrystallization from pentane. Yield: 51 mg, 56%. Yellow needle-shaped single crystals of this compound were grown from a pentane solution inside the glovebox freezer. ^{1}H NMR (500 MHz, C_6D_6) δ (ppm): 8.00 (s, 1H), 7.61 (t, *J* = 1.8 Hz, 2H), 7.52 (d, *J* = 1.9 Hz, 4H), 7.37–7.30 (m, 2H), 7.20 (d, *J* = 7.6 Hz, 2H), 7.03 (t, *J* = 7.7 Hz, 1H), 6.97 (d, *J* = 7.4 Hz, 1H), 6.77 (d, *J* = 8.3 Hz, 2H), 6.70 (d, *J* = 8.3 Hz, 2H), 6.55 (ddd, J_1 = 7.9 Hz, J_2 = 5.6 Hz, J_3 = 2.2 Hz, 1H), 2.12 (s, 3H), 1.36 (s, 36H). ^{13}C{^{1}H} NMR (126 MHz, C_6D_6) δ (ppm): 172.8, 165.8, 151.5, 151.4, 141.6, 137.0, 136.3, 134.5, 134.3, 129.65, 129.61, 129.55, 124.5, 123.9, 123.7, 122.9, 122.3, 119.4, 113.0, 35.2, 31.6, 20.7. The isocyanide ($\underline{C}$≡N) ^{13}C resonance was not located.

X-ray Crystallography

Single crystals were mounted on a Bruker Apex II three-circle diffractometer using MoKα radiation (λ=0.71073 Å, **Cu1**–**Cu4**) or a Bruker D8 Venture diffractometer using CuKα radiation (λ = 1.54184, **Cu5** and **Cu7**). The data were

collected at 123(2) K and processed and refined within the APEXII software. Structures were solved by intrinsic phasing in SHELXT and refined by standard difference Fourier techniques in the program SHELXL.[5] Hydrogen atoms were placed in calculated positions using the standard riding model and refined isotopically; all non-hydrogen atoms were refined anisotropically.

Table S1. Crystallographic Details of **Cu1** and **Cu2**.

	**Cu1**	**Cu2**
CCDC	2453584	2453585
Empirical formula	$C_{23}H_{21}N_2OCu$	$C_{24}H_{17}N_2OF_6Cu$
Formula weight	404.96	526.93
Temperature/K	123(2)	123(2)
Crystal system	monoclinic	triclinic
Space group	$P2_1/c$	$P\bar{1}$
a / Å	10.261(4)	9.201(5)
b / Å	26.202(9)	14.371(8)
c / Å	7.141(2)	17.738(10)
α / °	90	84.919(6)
β / °	90.436(5)	89.323(6)
γ / °	90	73.694(6)
Volume / Å^3	1919.9(11)	2242(2)
Z	4	4
ρ_{calc} / gcm^{-3}	1.401	1.561
μ / mm^{-1}	1.152	1.043
F(000)	840.0	1064.0
Crystal size / mm^3	0.42 × 0.1 × 0.06	0.26 × 0.21 × 0.05
Radiation	MoKα (λ = 0.71073)	MoKα (λ = 0.71073)
2Θ range for data collection / °	3.108 to 54.204	2.306 to 54.206
Index ranges	$-12 \le h \le 13$, $-30 \le k \le 33$, $-9 \le l \le 8$	$-11 \le h \le 11$, $-18 \le k \le 18$, $-22 \le l \le 22$
Reflections collected	11176	29846
Independent reflections	4215 [R_{int} = 0.0260, R_{sigma} = 0.0296]	9881 [R_{int} = 0.0331, R_{sigma} = 0.0404]
Data/restraints/parameters	4215/0/247	9881/116/618
Goodness-of-fit on F^2	1.033	1.041
Final R indexes [$I \ge 2\sigma(I)$]	R_1 = 0.0277, wR_2 = 0.0692	R_1 = 0.0398, wR_2 = 0.1038
Final R indexes [all data]	R_1 = 0.0350, wR_2 = 0.0725	R_1 = 0.0578, wR_2 = 0.1151
Largest diff. peak/hole / eÅ^{-3}	0.32/−0.27	1.30/−0.51

Table S2. Crystallographic Details of **Cu3** and **Cu4**.

	**Cu3**	**Cu4**
CCDC	2453586	2453587
Empirical formula	$C_{25}H_{25}N_2OCu$	$C_{34}H_{27}N_2OCu$
Formula weight	433.01	543.11
Temperature/K	123(2)	123(2)
Crystal system	triclinic	triclinic
Space group	$P\bar{1}$	$P\bar{1}$
a / Å	10.390(9)	9.466(3)
b / Å	10.781(9)	11.606(3)
c / Å	12.163(11)	13.062(4)
α / °	64.965(9)	93.187(3)
β / °	87.971(10)	92.645(3)
γ / °	66.385(9)	110.455(2)
Volume / Å^3	1116.2(17)	1339.2(7)
Z	2	2
ρ_{calc} / gcm^{-3}	1.288	1.347
μ / mm^{-1}	0.995	0.845
F(000)	452.0	564.0
Crystal size / mm^3	0.08 × 0.08 × 0.03	0.35 × 0.29 × 0.11
Radiation	MoKα (λ = 0.71073)	MoKα (λ = 0.71073)
2Θ range for data collection / °	3.744 to 53.464	3.756 to 54.204
Index ranges	$-13 \le h \le 13$, $-13 \le k \le 13$, $-15 \le l \le 15$	$-12 \le h \le 12$, $-14 \le k \le 14$, $-16 \le l \le 16$
Reflections collected	14113	18467
Independent reflections	4715 [R_{int} = 0.0445, R_{sigma} = 0.0552]	5874 [R_{int} = 0.0298, R_{sigma} = 0.0310]
Data/restraints/parameters	4715/0/267	5874/430/411
Goodness-of-fit on F^2	1.022	1.082
Final R indexes [$I \geq 2\sigma(I)$]	R_1 = 0.0384, wR_2 = 0.0787	R_1 = 0.0406, wR_2 = 0.0977
Final R indexes [all data]	R_1 = 0.0576, wR_2 = 0.0857	R_1 = 0.0464, wR_2 = 0.1007
Largest diff. peak/hole / eÅ^{-3}	0.37/−0.64	0.58/−0.69

Table S3. Crystallographic Details of **Cu5** and **Cu7**.

	**Cu5**	**Cu7**
CCDC	2453588	2453589
Empirical formula	$C_{27}H_{29}CuN_2O$	$C_{49}CuN_2OH_{57}$
Formula weight	461.06	753.50
Temperature/K	149.99	150.00
Crystal system	orthorhombic	monoclinic
Space group	*Pbca*	$P2_1/n$
a / Å	12.6043(9)	11.7921(9)
b / Å	18.9057(12)	13.6940(11)
c / Å	19.2930(13)	26.008(2)
α / °	90	90
β / °	90	100.426(4)
γ / °	90	90
Volume / Å^3	4597.4(5)	4130.4(6)
Z	8	4
ρ_{calc} / gcm^{-3}	1.332	1.212
μ / mm^{-1}	1.491	1.023
F(000)	1936.0	1608.0
Crystal size / mm^3	0.35 × 0.32 × 0.26	0.37 × 0.14 × 0.07
Radiation	CuKα (λ = 1.54184)	CuKα (λ = 1.54184)
2Θ range for data collection / °	9.168 to 136.764	6.912 to 136.736
Index ranges	$-15 \leq h \leq 15$, $-22 \leq k \leq 22$, −15 ≤ ` ≤ 23	$-14 \leq h \leq 11$, $-16 \leq k \leq 16$, $-31 \leq l \leq 31$
Reflections collected	18232	38978
Independent reflections	4147 [R_{int} = 0.0284, R_{sigma} = 0.0194]	7525 [R_{int} = 0.0303, R_{sigma} = 0.0203]
Data/restraints/parameters	4147/0/285	7525/0/491
Goodness-of-fit on F^2	1.051	1.034
Final *R* indexes [$I \geq 2\sigma(I)$]	R_1 = 0.0302, wR_2 = 0.0828	R_1 = 0.0331, wR_2 = 0.0917
Final *R* indexes [all data]	R_1 = 0.0309, wR_2 = 0.0832	R_1 = 0.0349, wR_2 = 0.0934
Largest diff. peak/hole / eÅ^{-3}	0.22/−0.45	0.34/−0.38

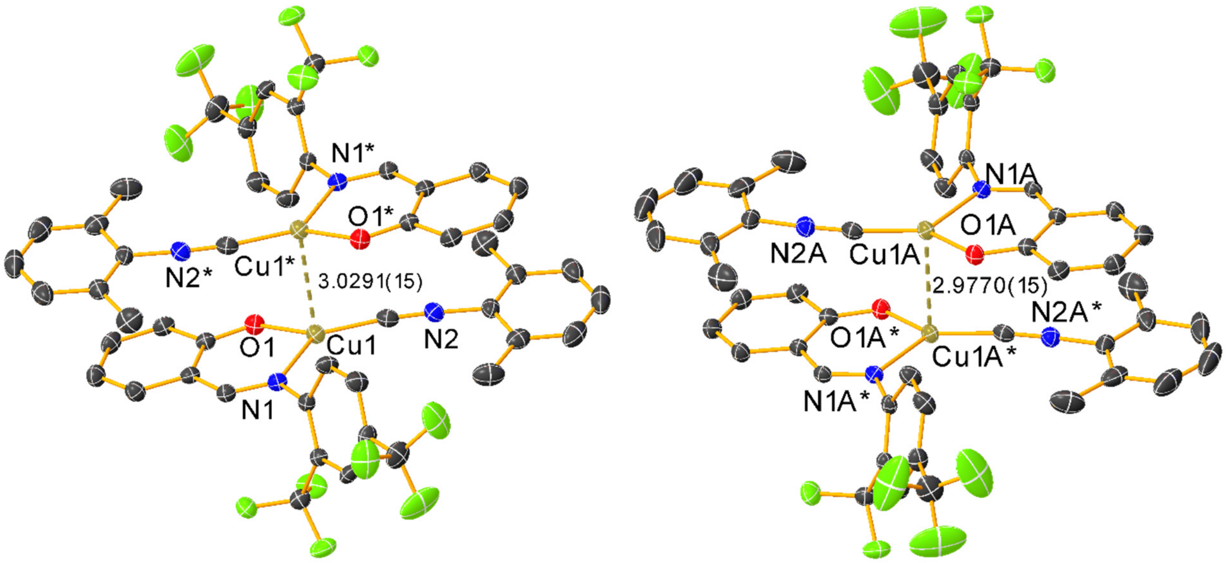

Fig. S1. Molecular structures of **Cu2**, determined by single-crystal X-ray diffraction, showing the dimeric structure that exists in the solid-state. Both crystallographically independent molecules are shown, each of which dimerizes with a symmetry-generated molecule via Cu···Cu interactions, with the internuclear distance shown. Atoms labeled with asterisks (*) are symmetry-generated. Fluorine atoms, shown in green, are unlabeled.

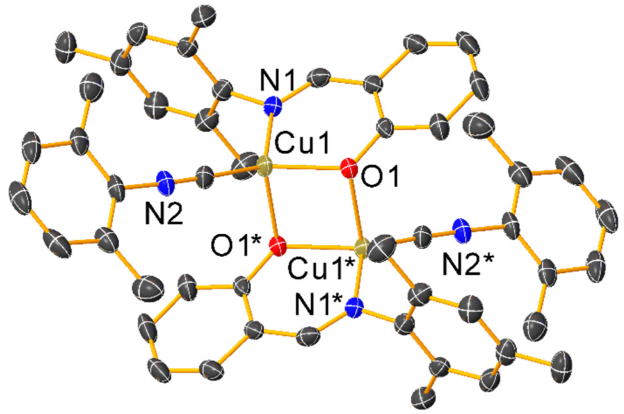

Fig. S2. Molecular structures of **Cu3**, determined by single-crystal X-ray diffraction, showing the dimeric structure that exists in the solid-state. The molecule dimerizes via Cu–O bonds involving the salicylaldimine oxygen atoms, forming a Cu_2O_2 diamond core. Atoms labeled with an asterisk (*) are generated by symmetry.

Table S4. Selected bond lengths and bond angles of **Cu1–Cu5** and **Cu7**.

	Bond Length (Å)	Bond Angles (º)
**Cu1**	Cu1–O1: 1.9347(13)	O1–Cu1–N1: 96.07(5)
	Cu1–N1: 1.9827(15)	O1–Cu1–C30: 132.76(6)
	Cu1–C30: 1.8190(17)	N1–Cu1–C30: 131.17(6)
**Cu2**[a]	Cu1–O1: 1.932(2), 1.926(2)	O1–Cu1–N1: 95.39(8), 96.00(8)
	Cu1–N1: 1.992(2), 1.986(2)	O1–Cu1–C30: 136.39(10), 135.67(9)
	Cu1–C30: 1.822(3), 1.817(3)	N1–Cu1–C30: 127.50(11), 127.50(10)
**Cu3**	Cu1–O1: 2.015(2)	O1–Cu1–N1: 91.74(8)
	Cu1–N1: 2.013(2)	O1–Cu1–C30: 131.47(9)
	Cu1–C30: 1.824(3)	N1–Cu1–C30: 128.33(10)
	Cu1–O1 (intermolecular): 2.242(2)	
**Cu4**	Cu1–O1: 1.9347(18)	O1–Cu1–N1: 95.08(7)
	Cu1–N1: 1.9886(17)	O1–Cu1–C50: 131.06(8)
	Cu1–C50: 1.816(2)	N1–Cu1–C50: 132.35(9)
**Cu5**	Cu1–O1: 1.9288(11)	O1–Cu1–N1: 96.96(5)
	Cu1–N1: 1.9824(11)	O1–Cu1–C15: 126.28(6)
	Cu1–C15: 1.8160(15)	N1–Cu1–C15: 136.35(6)
**Cu7**	Cu1–O1: 1.9323(10)	O1–Cu1–N1: 96.33(5)
	Cu1–N1: 1.9859(12)	O1–Cu1–C40: 125.76(5)
	Cu1–C40: 1.8164(15)	N1–Cu1–C40: 137.24(6)

[a]In **Cu2** there are two crystallographically independent molecules, so both sets of values are provided.

Photophysical Studies: Overlaid UV–vis and excitation spectra of Cu1-Cu7.

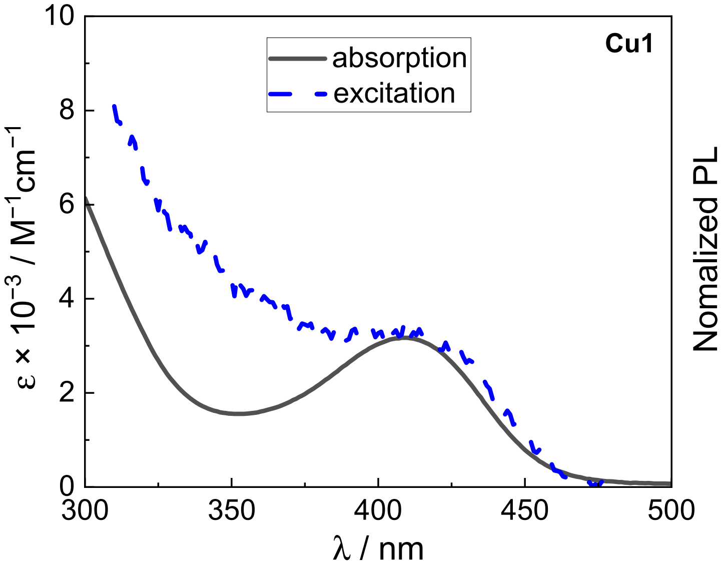

Fig. S3. Overlaid UV–vis absorption spectrum (solid black line) and excitation spectrum (dashed blue line) of **Cu1**, recorded in toluene at room temperature. The excitation spectrum is intensity-matched to the absorption spectrum at the long wavelength maximum.

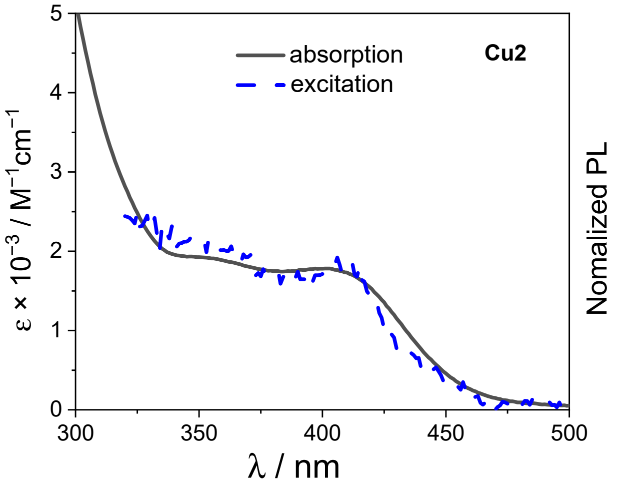

Fig. S4. Overlaid UV–vis absorption spectrum (solid black line) and excitation spectrum (dashed blue line) of **Cu2**, recorded in toluene at room temperature. The excitation spectrum is intensity-matched to the absorption spectrum at the long wavelength maximum.

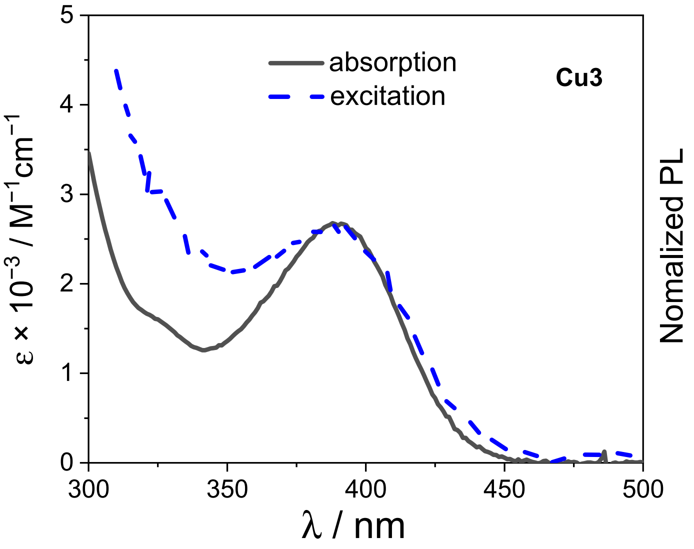

Fig. S5. Overlaid UV–vis absorption spectrum (solid black line) and excitation spectrum (dashed blue line) of **Cu3**, recorded in toluene at room temperature. The excitation spectrum is intensity-matched to the absorption spectrum at the long wavelength maximum.

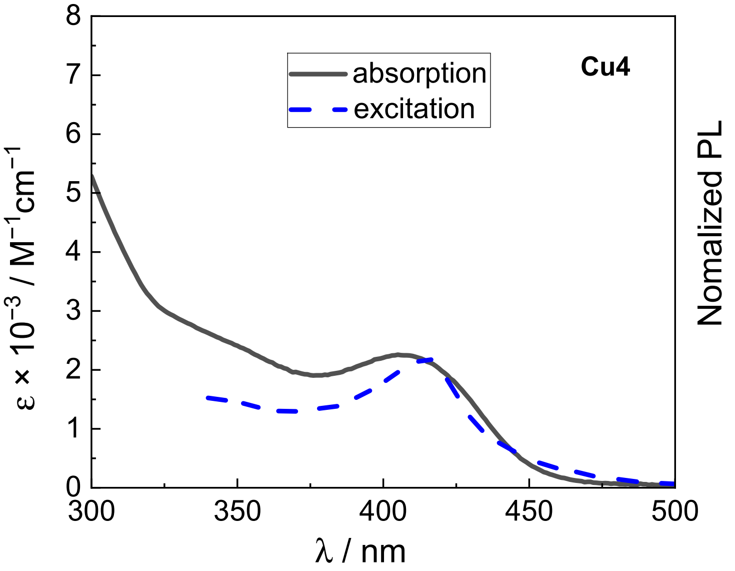

Fig. S6. Overlaid UV–vis absorption spectrum (solid black line) and excitation spectrum (dashed blue line) of **Cu4**, recorded in toluene at room temperature. The excitation spectrum is intensity-matched to the absorption spectrum at the long wavelength maximum.

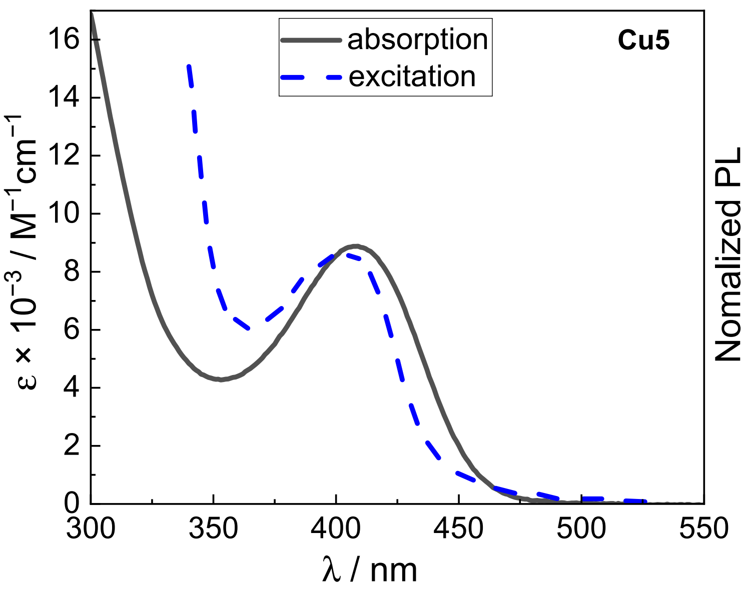

Fig. S7. Overlaid UV–vis absorption spectrum (solid black line) and excitation spectrum (dashed blue line) of **Cu5**, recorded in toluene at room temperature. The excitation spectrum is intensity-matched to the absorption spectrum at the long wavelength maximum.

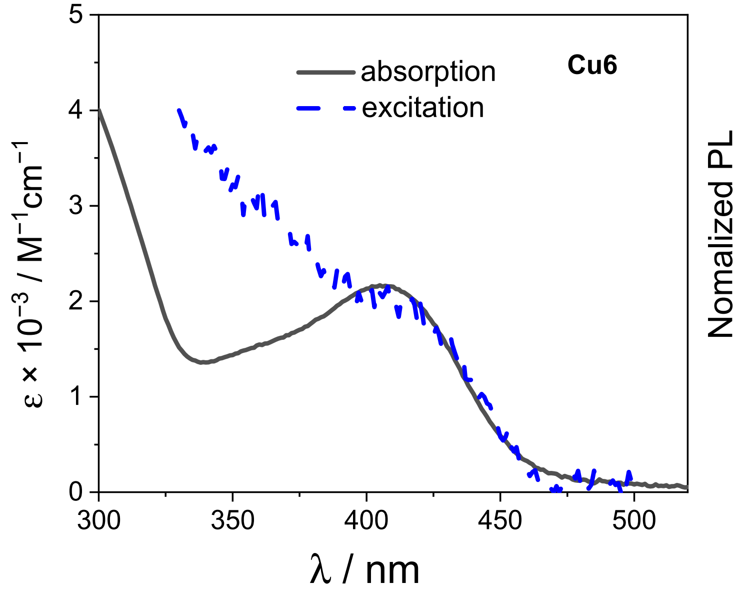

Fig. S8. Overlaid UV–vis absorption spectrum (solid black line) and excitation spectrum (dashed blue line) of **Cu6**, recorded in toluene at room temperature. The excitation spectrum is intensity-matched to the absorption spectrum at the long wavelength maximum.

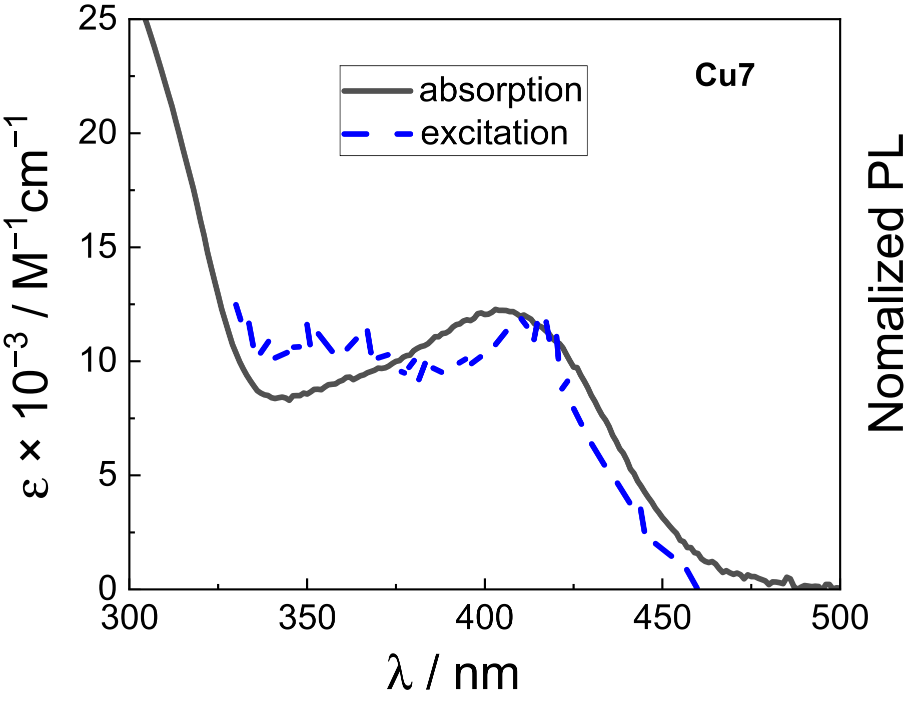

Fig. S9. Overlaid UV–vis absorption spectrum (solid black line) and excitation spectrum (dashed blue line) of **Cu7**, recorded in toluene at room temperature. The excitation spectrum is intensity-matched to the absorption spectrum at the long wavelength maximum.

Photoluminescence measurement for Cu1 in 2% PMMA film

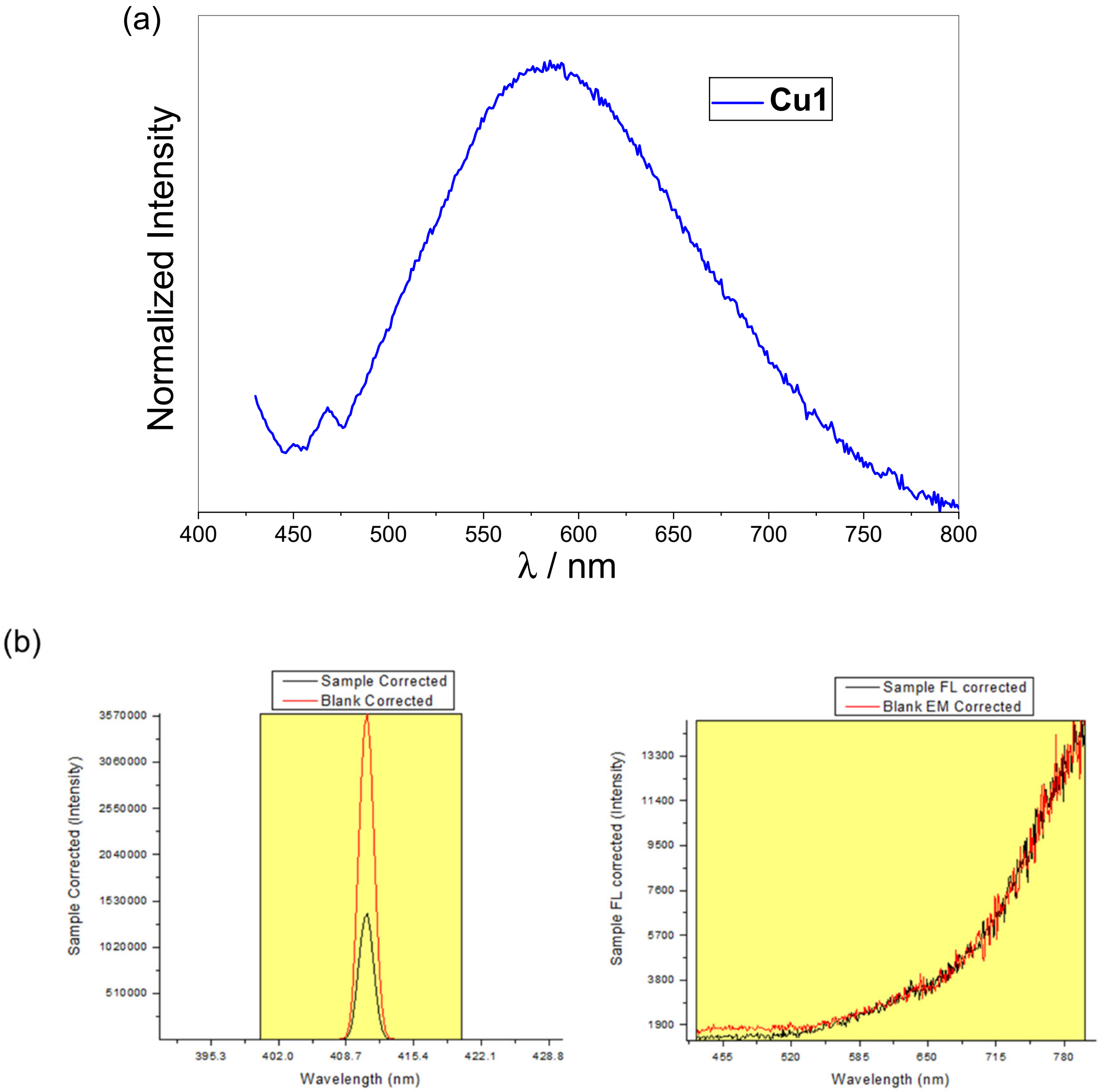

Fig. S10. (a) Photoluminescence spectrum of **Cu1**, recorded in 2% PMMA film at room temperature. (b) Data from an attempted quantum yield measurement on the same sample. The first panels overlay excitation (left) and emission (right) spectra of the **Cu1** film (2 wt% in PMMA and blank (pure PMMA film) , recorded in an integrating sphere. Recorded in this way, the PL signal of the sample is imperceptibly higher than that of the blank, obviating reliable quantum yield measurement.

Time-resolved photoluminescence decay traces

In most cases, the TCSPC decay curves were best fit with a biexponential function (Equation S2):

$$I(t) = A_1 e^{-t/\tau 1} + A_2 e^{-t/\tau 2} \qquad \text{S2}$$

where A_1 and A_2 are pre-exponential factors and τ_1 and τ_2 are lifetimes. Average lifetimes (τ_{avg}) were calculated as an amplitude-weighted mean (Equation S3):

$$\tau_{avg} = \frac{A_1\tau_1^2 + A_2\tau_2^2}{A_1\tau_1 + A_2\tau_2} \qquad \text{S3}$$

The exception is **Cu4**, where the decay trace could be satisfactorily fit by a monoexponential equation (first term of Equation S2).

For the decays best described by a biexponential model, monoexponential fits gave higher χ^2 values and there was periodicity in the residuals at early time points. The fitting range was selected by taking the instrument response function (IRF) into account, which presents some early time background signal that is especially pronounced in weakly emitting samples like these. The fitting range started just after the IRF decay until the PL signal reached the baseline. In a few cases, the early part of the decay contained additional artifacts, so the fits were started at later times (≈100 ns) to get reliable results. In all cases, the fitting range is at least $5\times\tau_{avg}$. Plots showing experimental decay (points), the biexponential fit (solid line), and the residuals (Exp − Fit) are presented below for each compound. The residuals are randomly distributed around zero and remain within ±3σ of the baseline noise. The reduced chi-square values for all fits are in the range of 1.09–1.37.

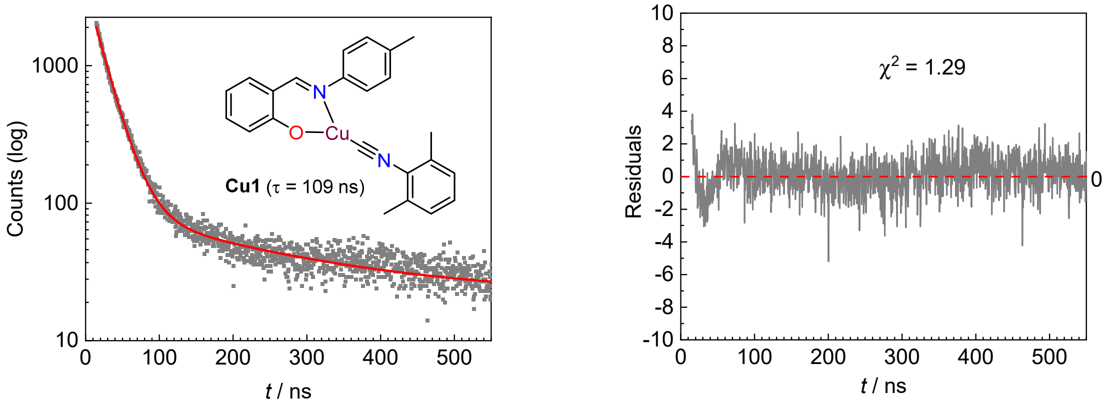

Fig. S11. Time-resolved photoluminescence decay of the **Cu1** obtained by time-correlated single photon counting (TCSPC). Left: Experimental data (points) with the biexponential fit (solid line); Right: The corresponding residuals (Exp − Fit). The decay trace was recorded in toluene with 390 nm excitation wavelength and was best fit by a bi-exponential equation.

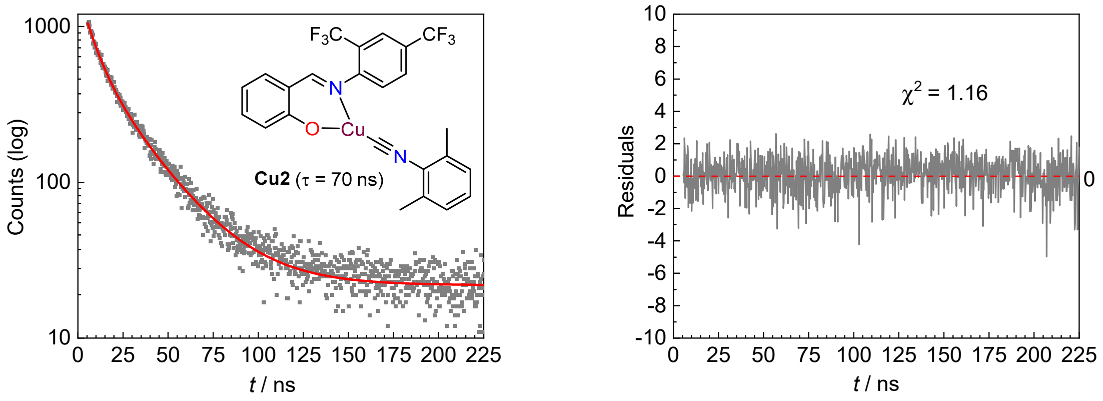

Fig. S12. Time-resolved photoluminescence decay of the **Cu2** obtained by time-correlated single photon counting (TCSPC). Left: Experimental data (points) with the biexponential fit (solid line); Right: The corresponding residuals (Exp − Fit). The decay trace was recorded in toluene with 390 nm excitation wavelength and was best fit by a bi-exponential equation.

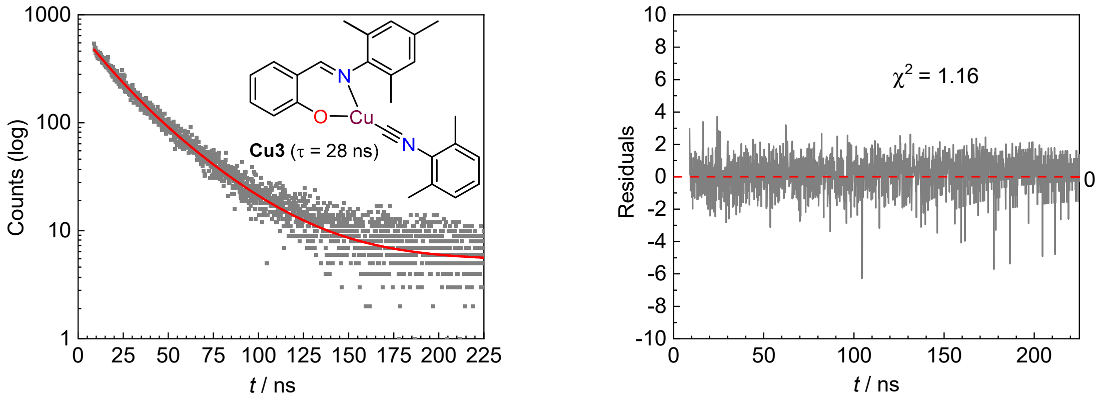

Fig. S13. Time-resolved photoluminescence decay of the **Cu3** obtained by time-correlated single photon counting (TCSPC). Left: Experimental data (points) with the biexponential fit (solid line); Right: The corresponding residuals (Exp − Fit). The decay trace was recorded in toluene with 390 nm excitation wavelength and was best fit by a bi-exponential equation.

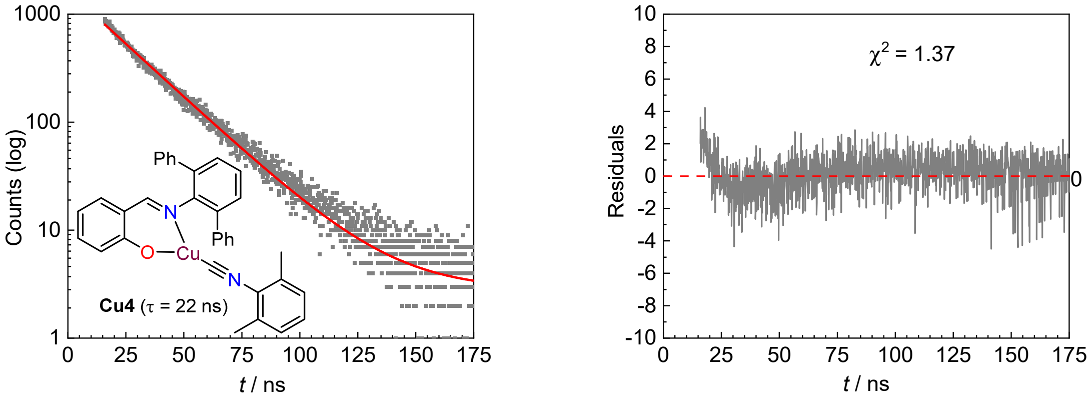

Fig. S14. Time-resolved photoluminescence decay of the **Cu4** obtained by time-correlated single photon counting (TCSPC). Left: Experimental data (points) with the biexponential fit (solid line); Right: The corresponding residuals (Exp − Fit). The decay trace was recorded in toluene with 390 nm excitation wavelength and was best fit by a mono-exponential equation.

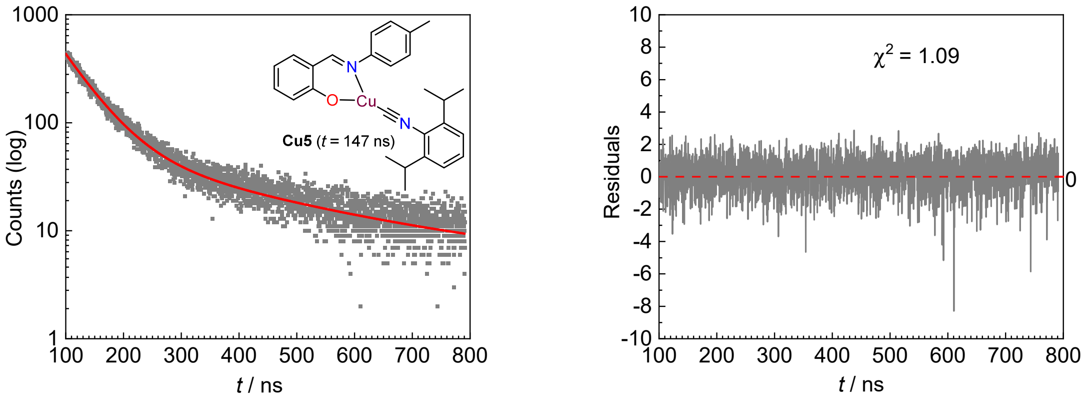

Fig. S15. Time-resolved photoluminescence decay of the **Cu5** obtained by time-correlated single photon counting (TCSPC). Left: Experimental data (points) with the biexponential fit (solid line); Right: The corresponding residuals (Exp − Fit). The decay trace was recorded in toluene with 390 nm excitation wavelength and was best fit by a bi-exponential equation.

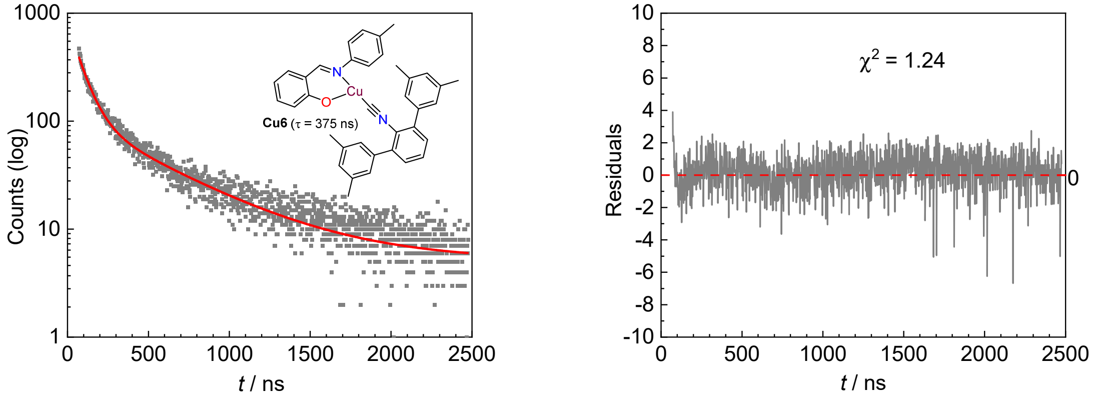

Fig. S16. Time-resolved photoluminescence decay of the **Cu6** obtained by time-correlated single photon counting (TCSPC). Left: Experimental data (points) with the biexponential fit (solid line); Right: The corresponding residuals (Exp − Fit). The decay trace was recorded in toluene with 390 nm excitation wavelength and was best fit by a bi-exponential equation.

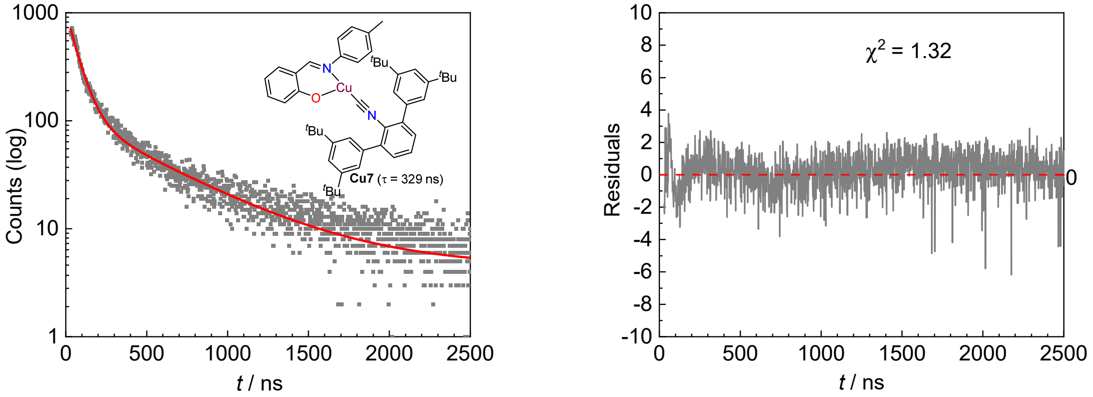

Fig. S17. Time-resolved photoluminescence decay of the **Cu7** obtained by time-correlated single photon counting (TCSPC). Left: Experimental data (points) with the biexponential fit (solid line); Right: The corresponding residuals (Exp − Fit). The decay trace was recorded in toluene with 390 nm excitation wavelength and was best fit by a bi-exponential equation.

Photostability test of Cu2

To check the photostability of **Cu2**, it was dissolved in C_6D_6 and transferred to an NMR tube in a nitrogen-filled glovebox. The NMR tube was sealed with parafilm and exposed to light (430–500 nm range). ^{1}H NMR spectra were recorded before light irradiation (t = 0 min) and at different time intervals up to 2 hours.

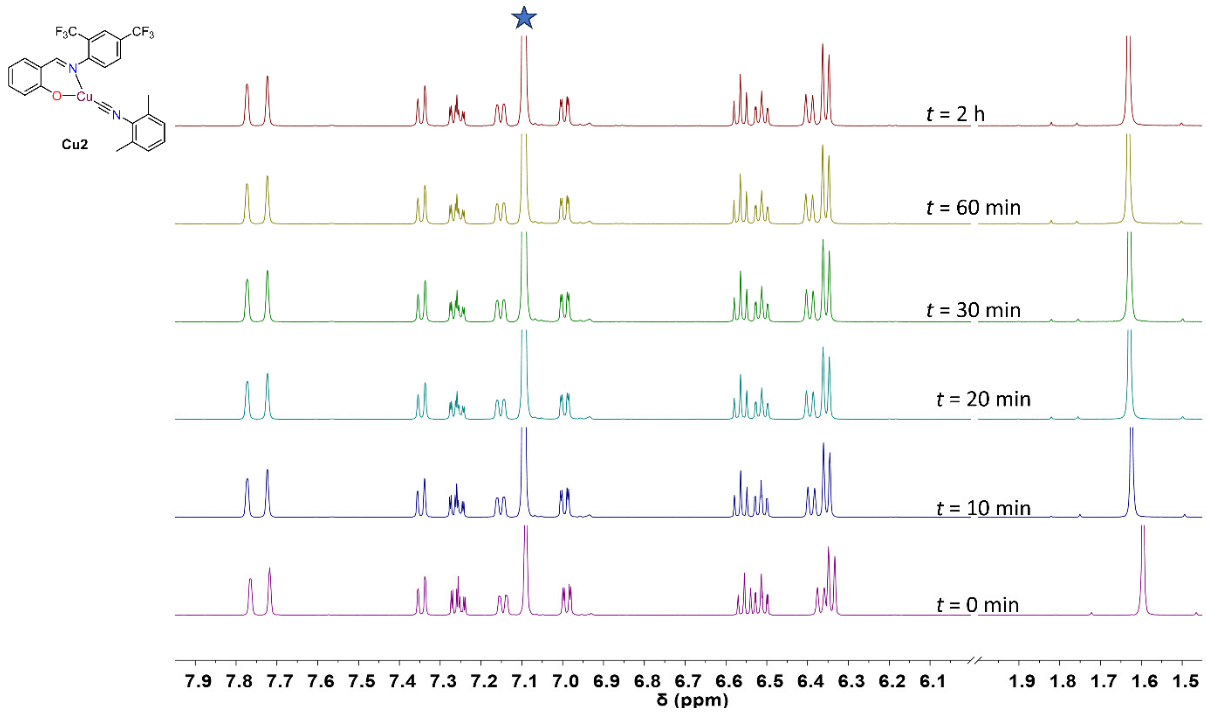

Fig. S18. Photostability test of **Cu2**: stacked ^{1}H NMR spectra recorded at different time interval of light irradiation. Solvent peaks and C_6D_6 residual are marked.

Photocatalysis

Photocatalytic reactions were carried out using a home-built photoreactor that consists of a glass dish wrapped with blue LED strips (purchased from Creative Lighting Solutions, Model: Blue Flexible LED Strips, 12 vdc. Emission profile: 430–500 nm) on the inside wall with the outside wall wrapped with aluminum foil.

***E/Z* isomerization of (*E*)-stilbene**

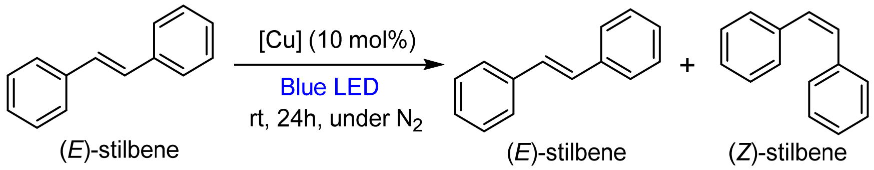

In a nitrogen-filled glovebox, (*E*)-stilbene (6.3 mg, 0.035 mmol), the copper photosensitizer (10 mol%) and 1,3,5-trimethoxybenzene (6 mg, 0.035 mmol) as an internal standard were taken in a small vial and dissolved in 0.6 mL of C_6D_6. The reaction mixture was transferred to an NMR tube which was further sealed with parafilm and taken out of glovebox. The NMR tube was placed inside the photoreactor and irradiated with blue LED light. For control reactions, a similar set up was made either (i) without using the catalyst; or (ii) the NMR tube was placed in dark for 24 h. All the reactions were monitored by ^{1}H NMR spectroscopy. The *E*/*Z* ratio was determined by ^{1}H NMR spectroscopy, integrating relative to the internal standard.

Table S5. *E/Z* isomerization of (*E*)-stilbene with variable Cu catalysts.

Catalyst	Time	*E*:*Z*	Catalyst	Time	*E*:*Z*
**Cu1**	0 h	100:0	**Cu2**	0 h	100:0
	30 min	26:74		30 min	88:12
	1 h	11:89		1 h	75:25
	2 h	11:89		2 h	50:50
	4 h	11:89		4 h	33:67
	24 h	11:89		24 h	11:89
**Cu3**	0 h	100:0	**Cu4**	0 h	100:0
	30 min	74:26		30 min	63:37
	1 h	47:53		1 h	36:64
	2 h	22:78		2 h	19:81
	4 h	19:81		4 h	17:83
	24 h	17:83		24 h	17:83
**Cu5**	0 h	100:0	**Cu6**	0 h	100:0
	30 min	14:86		30 min	17:83
	1 h	14:86		1 h	12:88
	2 h	14:86		2 h	12:88
	4 h	14:86		4 h	12:88
	24 h	14:86		24 h	11:89
**Cu7**	0 h	100:0			
	30 min	49:51			
	1 h	21:79			
	2 h	11:89			
	4 h	11:89			
	24 h	11:89			

Table S6. Kinetic monitoring of *E/Z* isomerization of (*E*)-stilbene with variable Cu catalysts.

Catalyst	Time	E:Z	Catalyst	Time	E:Z
**Cu1**	0 min	100:0	**Cu5**	0 h	100:0
	10 min	59:41		10 min	63:37
	20 min	32:68		20 min	34:66
	35 min	21:79		35 min	14:86
	60 min	11:89		60 min	14:86
**Cu6**	0 h	100:0	**Cu7**	0 h	100:0
	10 min	58:42		10 min	70:30
	20 min	23:77		20 min	45:55
	35 min	11:89		35 min	25:75
	60 min	11:89		60 min	19:81

^{1}H NMR spectra monitoring photocatalytic reactions

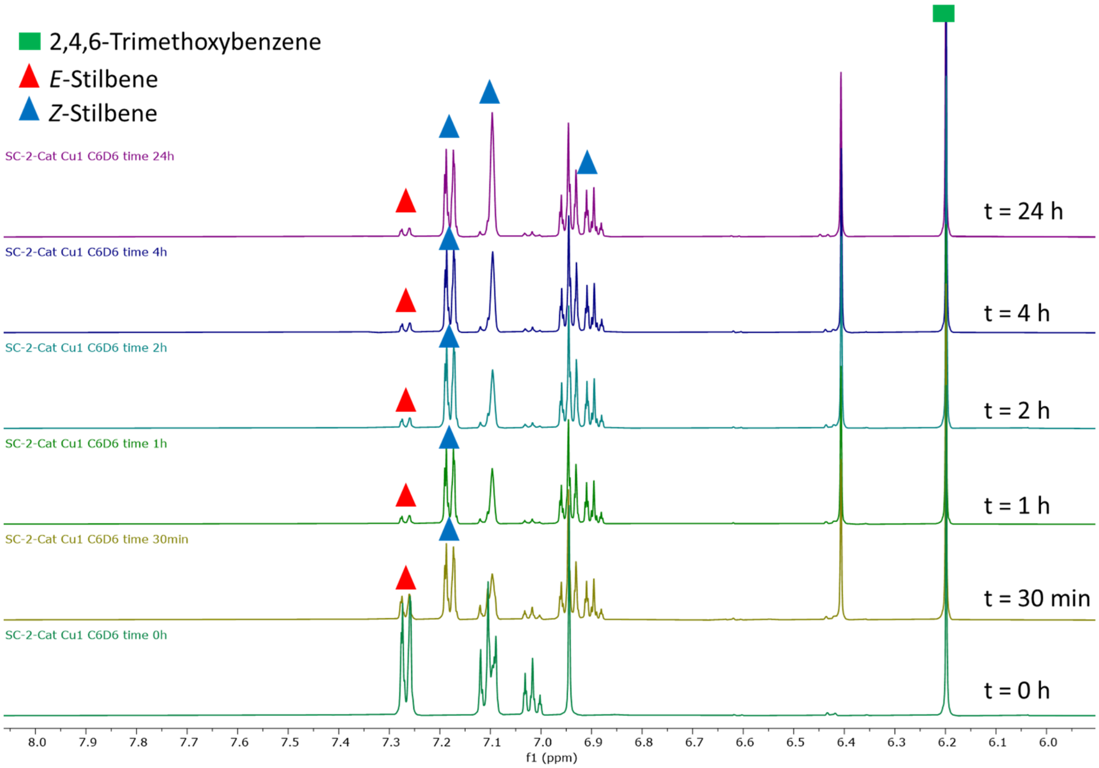

Fig. S19. Stacked ^{1}H NMR spectra in C_6D_6 representing the photocatalytic *E/Z* isomerization of *(E)*-stilbene using **Cu1** as photocatalyst.

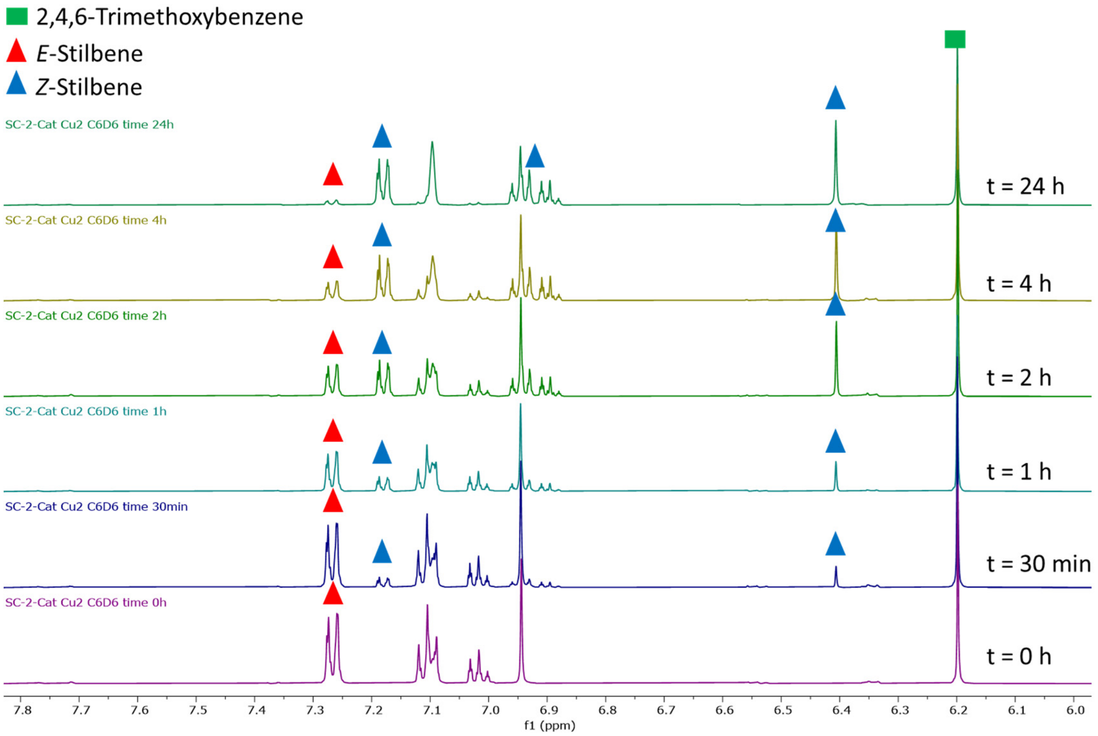

Fig. S20. Stacked ^{1}H NMR spectra in C_6D_6 representing the photocatalytic *E/Z* isomerization of *(E)*-stilbene using **Cu2** as photocatalyst.

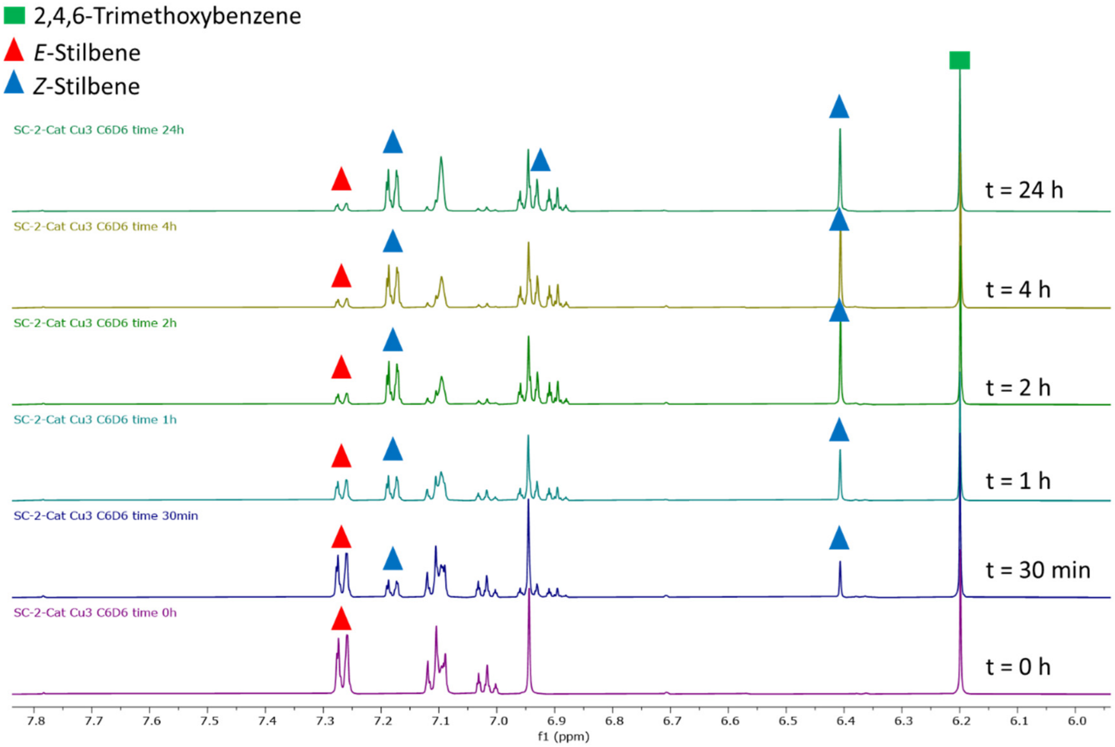

Fig. S21. Stacked ^{1}H NMR spectra in C_6D_6 representing the photocatalytic *E/Z* isomerization of *(E)*-stilbene using **Cu3** as photocatalyst.

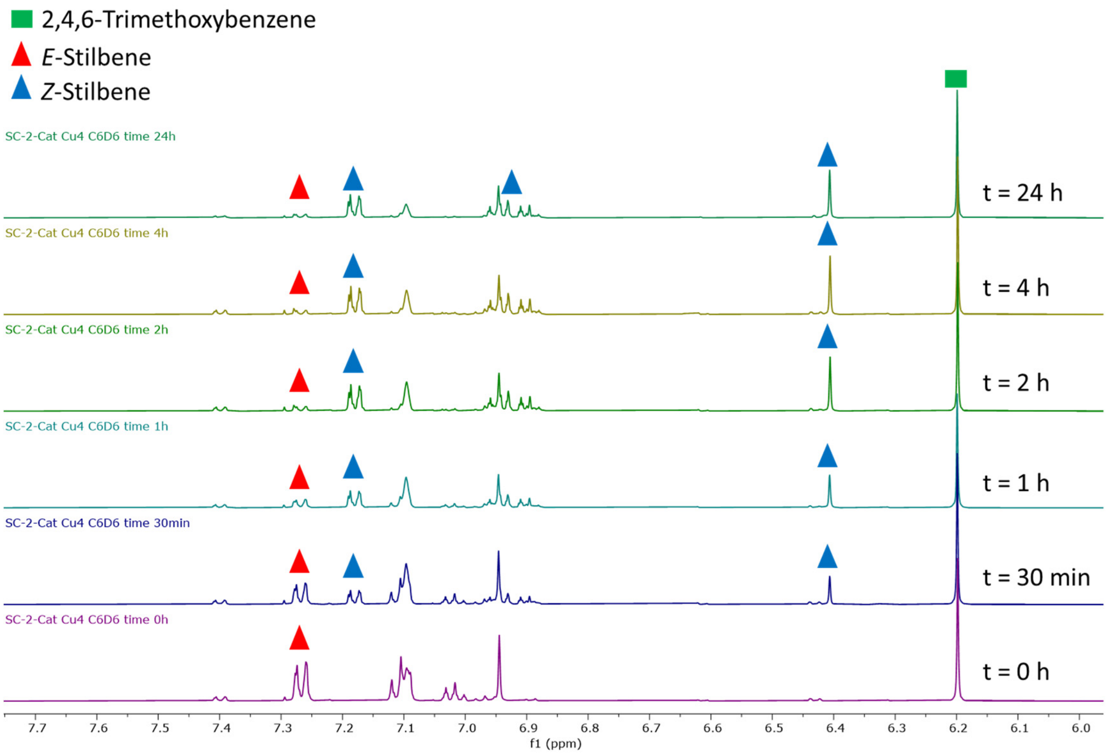

Fig. S22. Stacked ^{1}H NMR spectra in C_6D_6 representing the photocatalytic *E/Z* isomerization of *(E)*-stilbene using **Cu4** as photocatalyst.

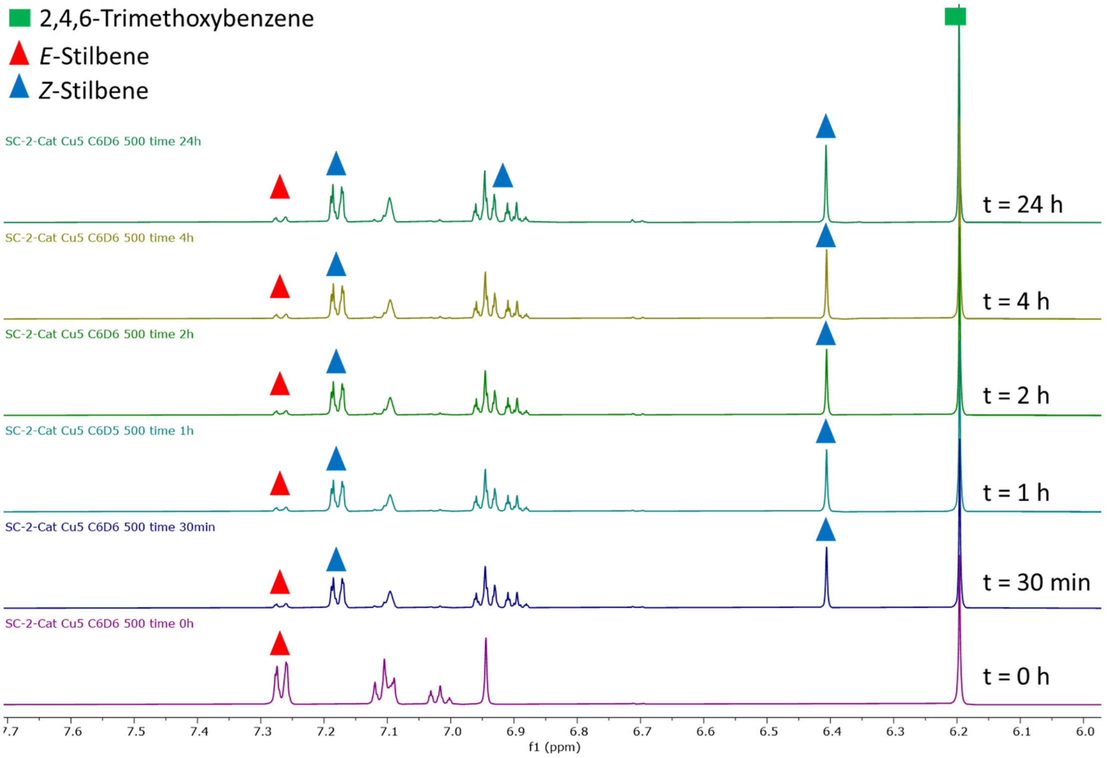

Fig. S23. Stacked ^{1}H NMR spectra in C_6D_6 representing the photocatalytic *E/Z* isomerization of *(E)*-stilbene using **Cu5** as photocatalyst.

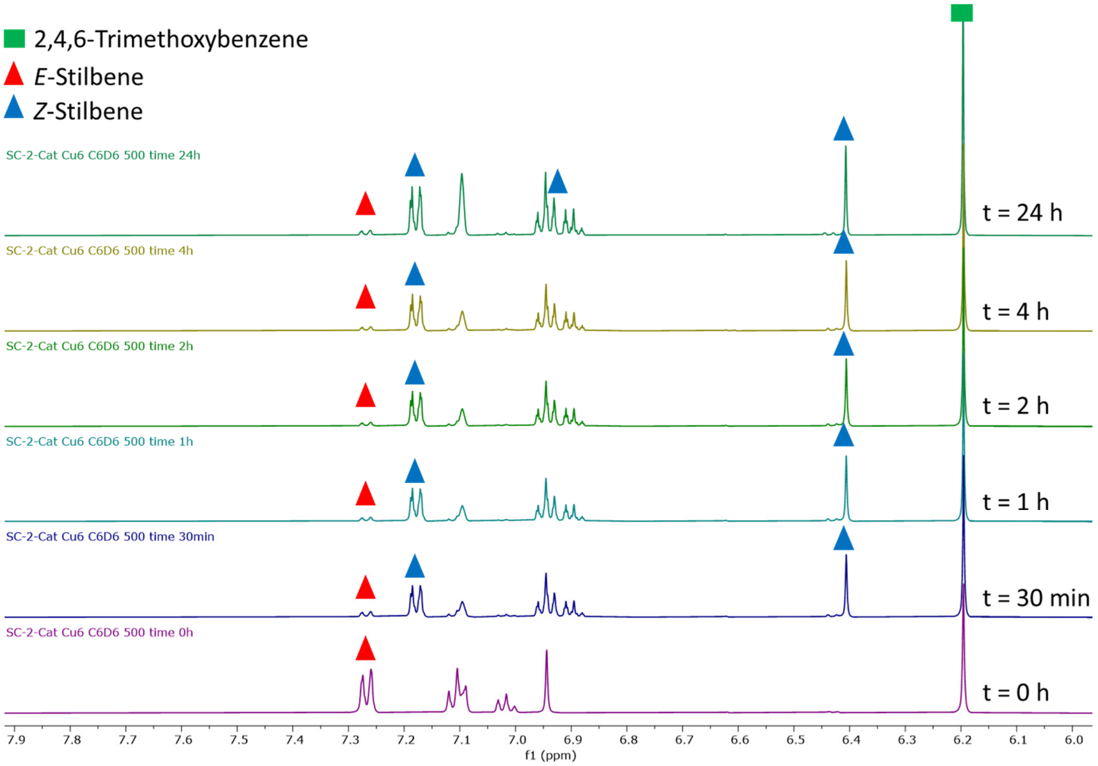

Fig. S24. Stacked ^{1}H NMR spectra in C_6D_6 representing the photocatalytic *E/Z* isomerization of *(E)*-stilbene using **Cu6** as photocatalyst.

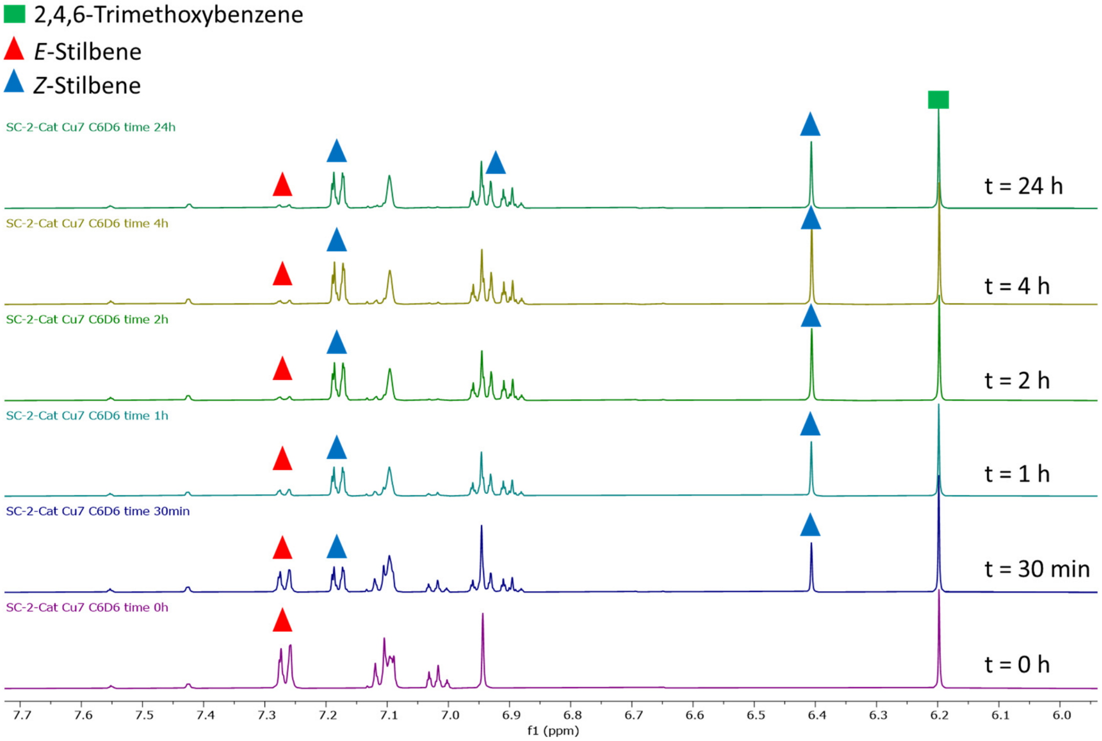

Fig. S25. Stacked ^{1}H NMR spectra in C_6D_6 representing the photocatalytic *E/Z* isomerization of *(E)*-stilbene using **Cu7** as photocatalyst.

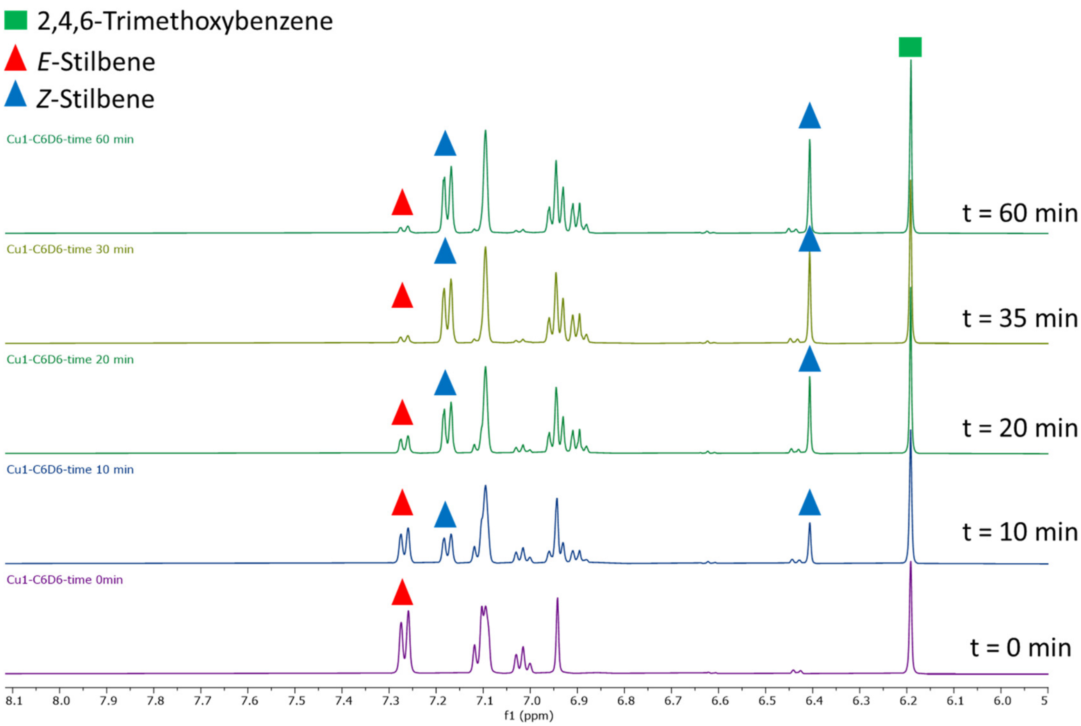

Fig. S26. Stacked ^{1}H NMR spectra in C_6D_6 representing the photocatalytic *E/Z* isomerization of *(E)*-stilbene using **Cu1** as photocatalyst, monitored over a 1 hour period.

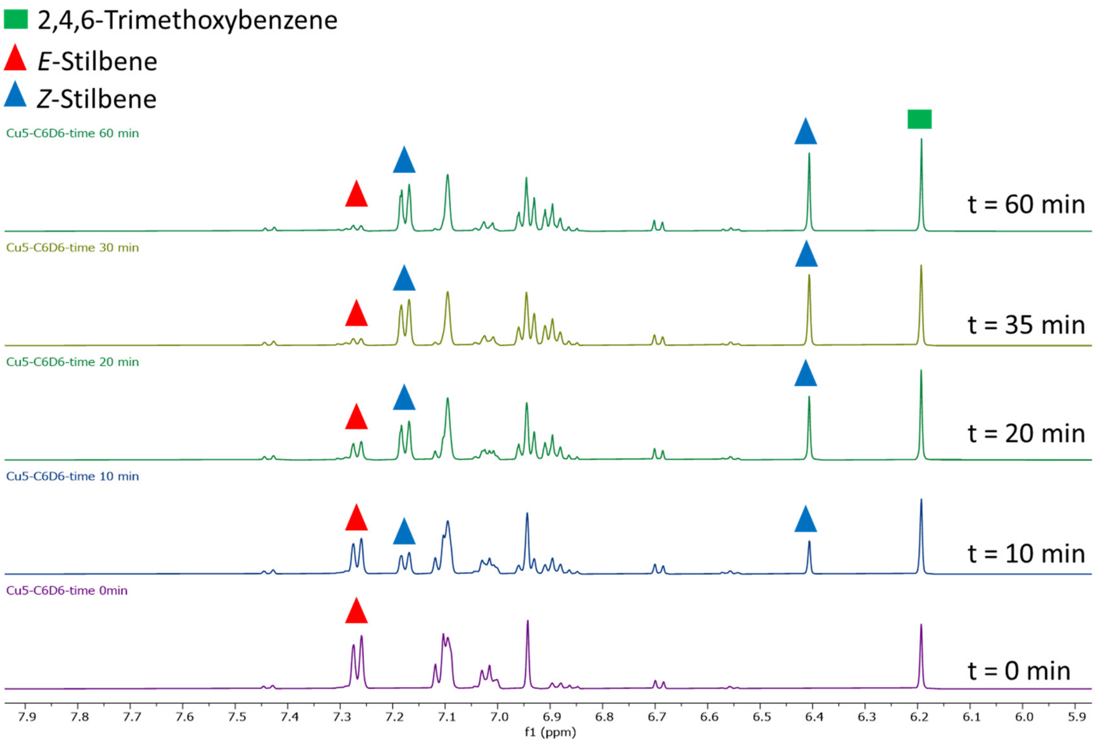

Fig. S27. Stacked ^{1}H NMR spectra in C_6D_6 representing the photocatalytic *E/Z* isomerization of *(E)*-stilbene using **Cu5** as photocatalyst, monitored over a 1 hour period.

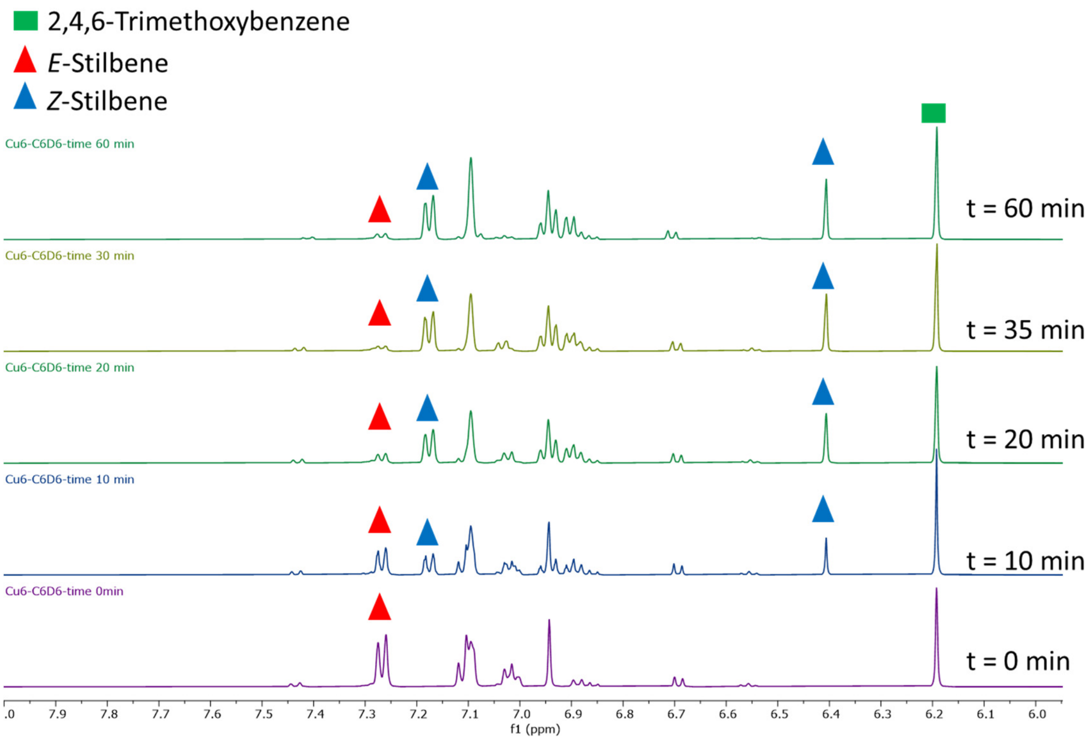

Fig. S28. Stacked ^{1}H NMR spectra in C_6D_6 representing the photocatalytic *E/Z* isomerization of *(E)*-stilbene using **Cu6** as photocatalyst, monitored over a 1 hour period.

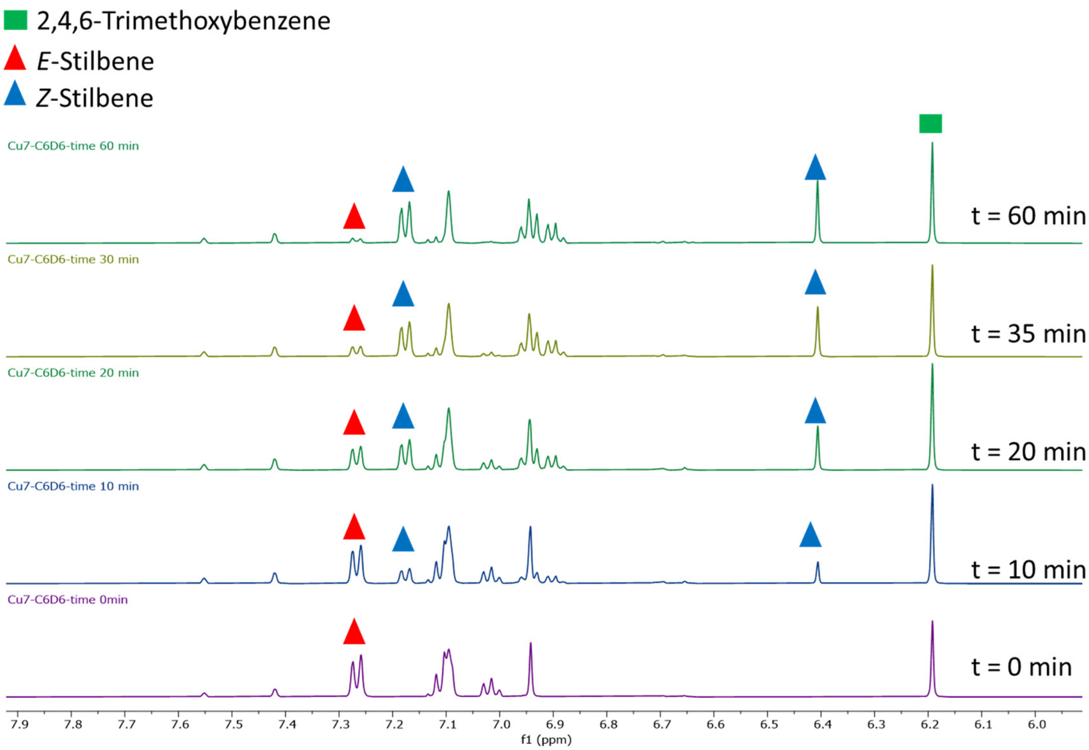

Fig. S29. Stacked ^{1}H NMR spectra in C_6D_6 representing the photocatalytic *E/Z* isomerization of *(E)*-stilbene using **Cu7** as photocatalyst, monitored over a 1 hour period.

NMR spectra of Cu1–Cu7

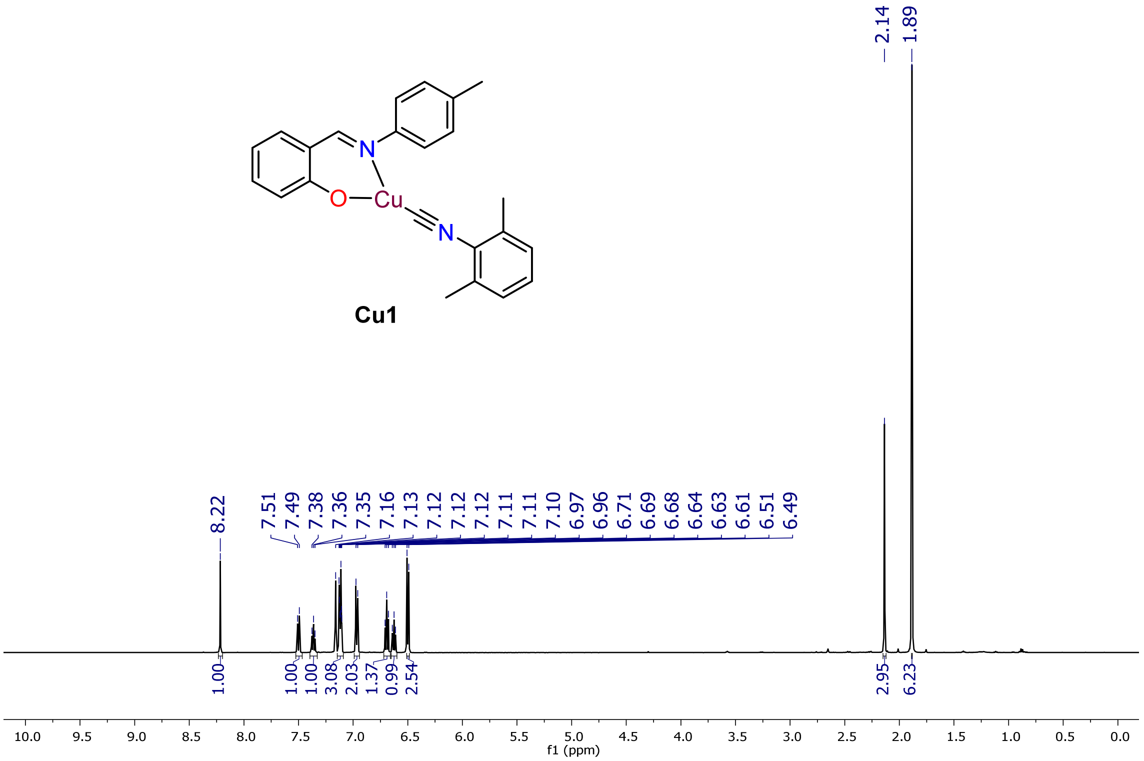

Fig. S30. ^{1}H NMR spectrum of **Cu1**, recorded at room temperature in C_6D_6 at 500 MHz.

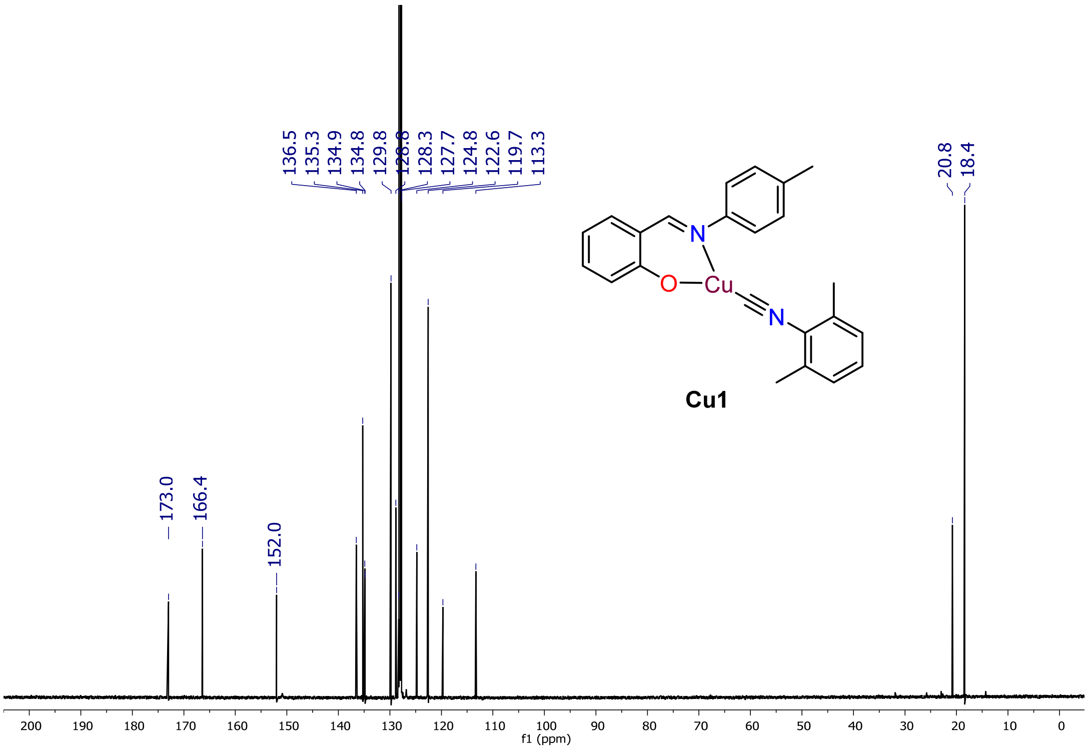

Fig. S31. $^{13}C\{^{1}H\}$ NMR spectrum of **Cu1**, recorded at room temperature in C_6D_6 at 126 MHz.

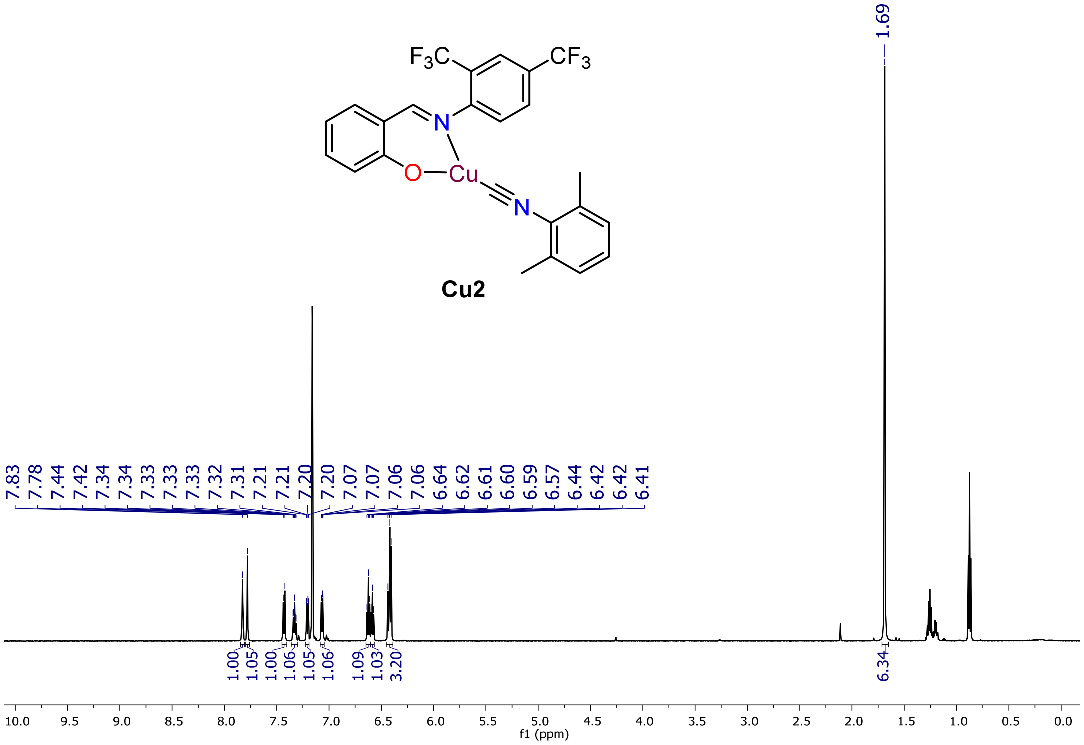

Fig. S32. ^{1}H NMR spectrum of **Cu2**, recorded at room temperature in C_6D_6 at 500 MHz.

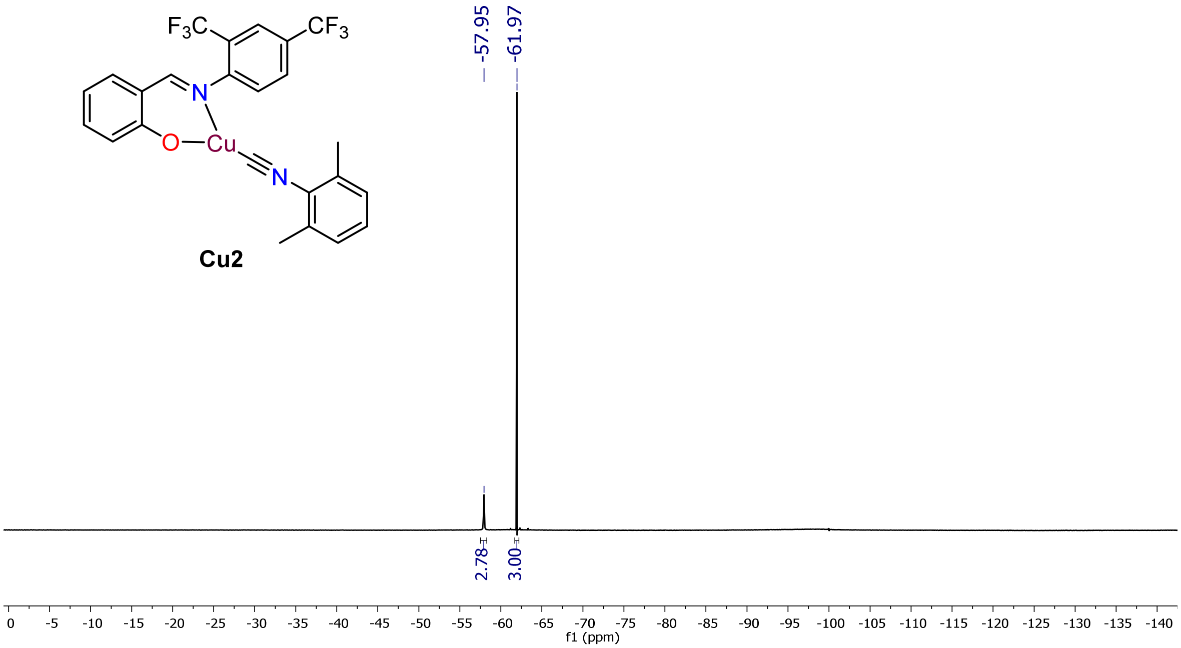

Fig. S33. ^{19}F NMR spectrum of **Cu2**, recorded at room temperature in C_6D_6 at 565 MHz.

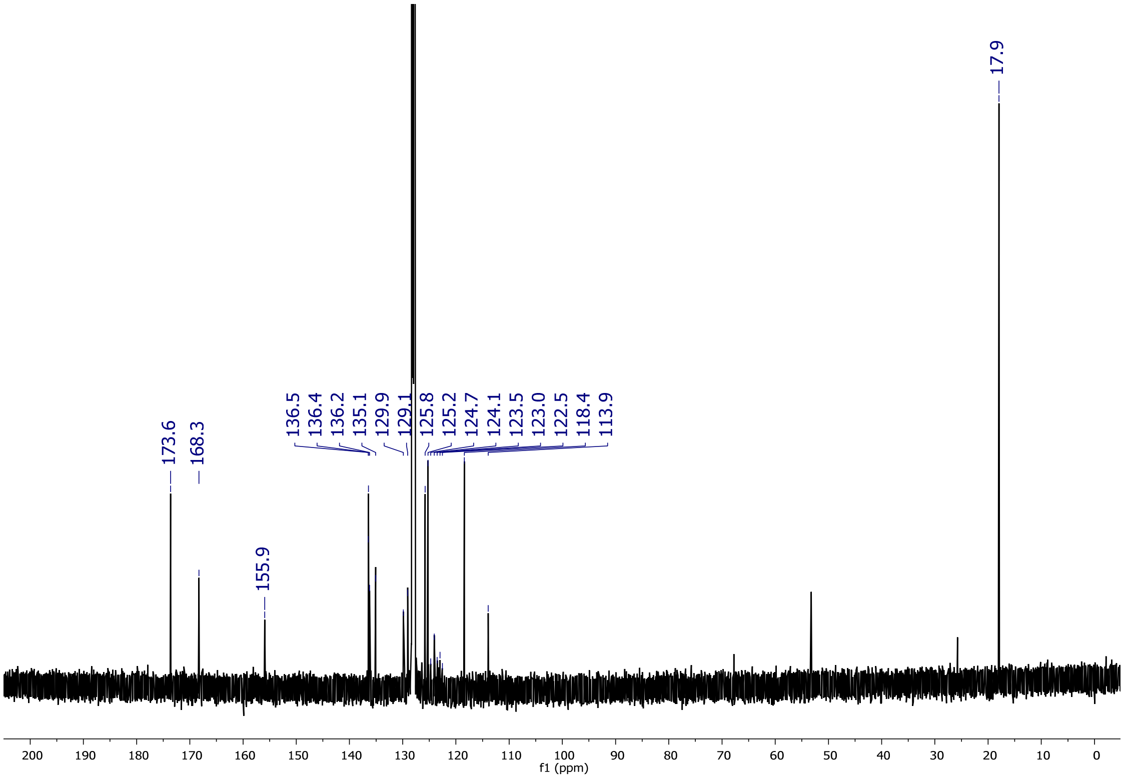

Fig. S34. $^{13}C\{^{1}H\}$ NMR spectrum of **Cu2**, recorded at room temperature in C_6D_6 at 126 MHz.

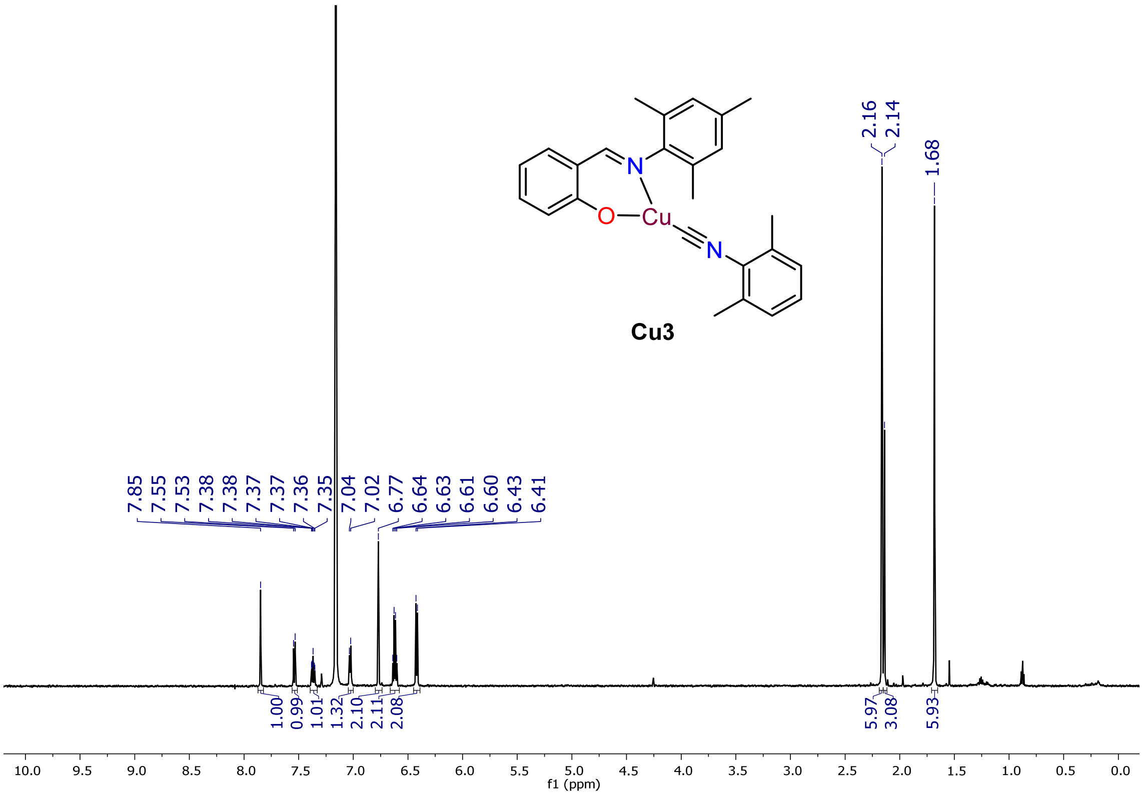

Fig. S35. ^{1}H NMR spectrum of **Cu3**, recorded at room temperature in C_6D_6 at 500 MHz.

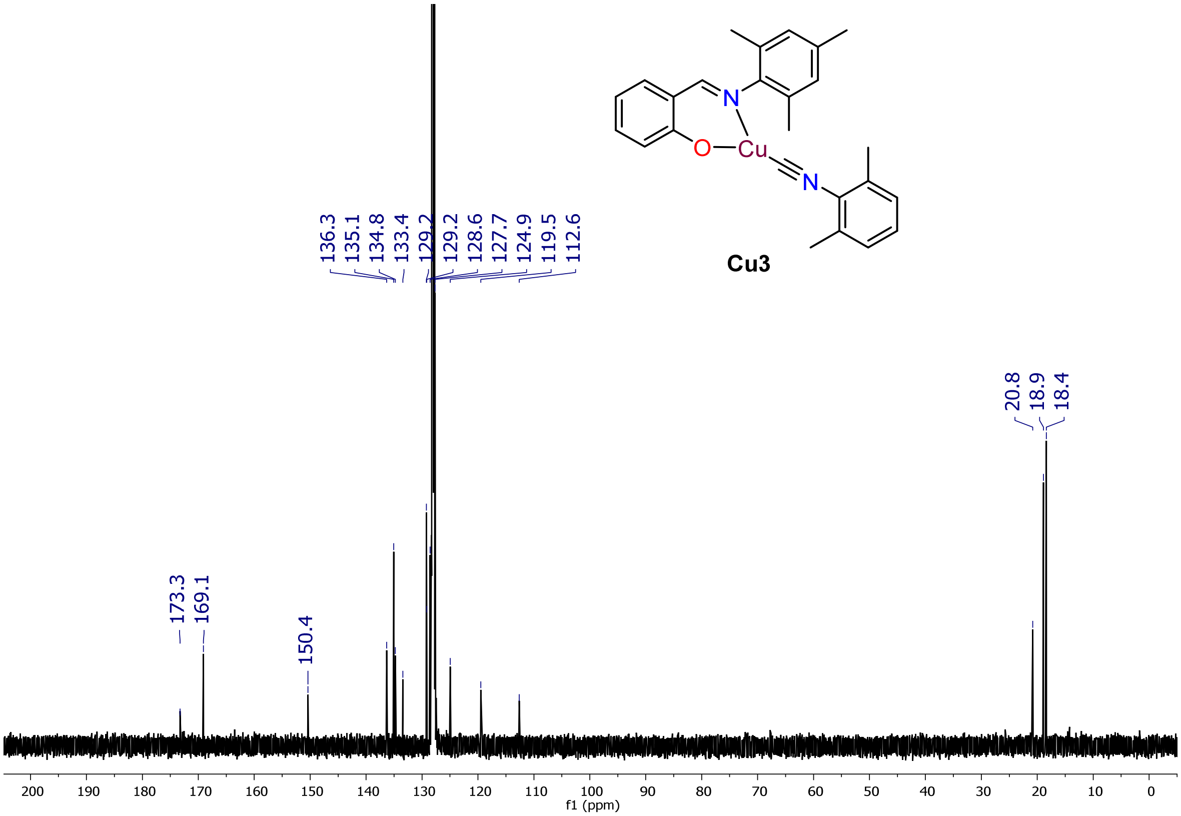

Fig. S36. $^{13}C\{^1H\}$ NMR spectrum of **Cu3**, recorded at room temperature in C_6D_6 at 126 MHz.

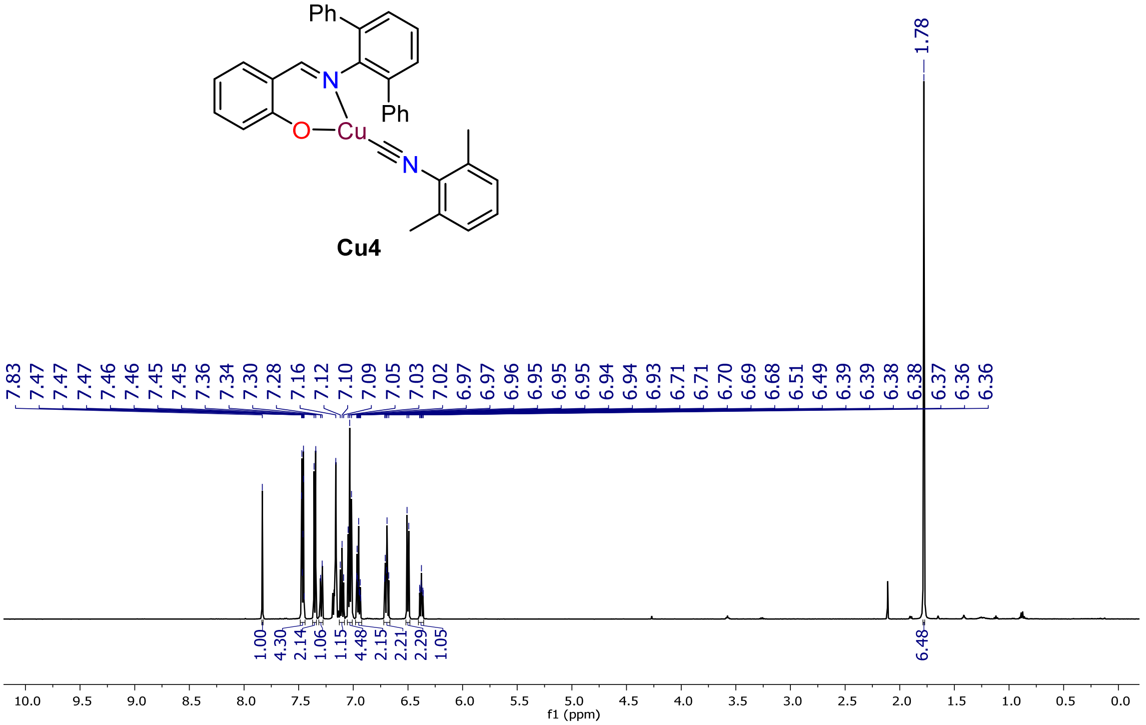

Fig. S37. ^1H NMR spectrum of **Cu4**, recorded at room temperature in C_6D_6 at 500 MHz.

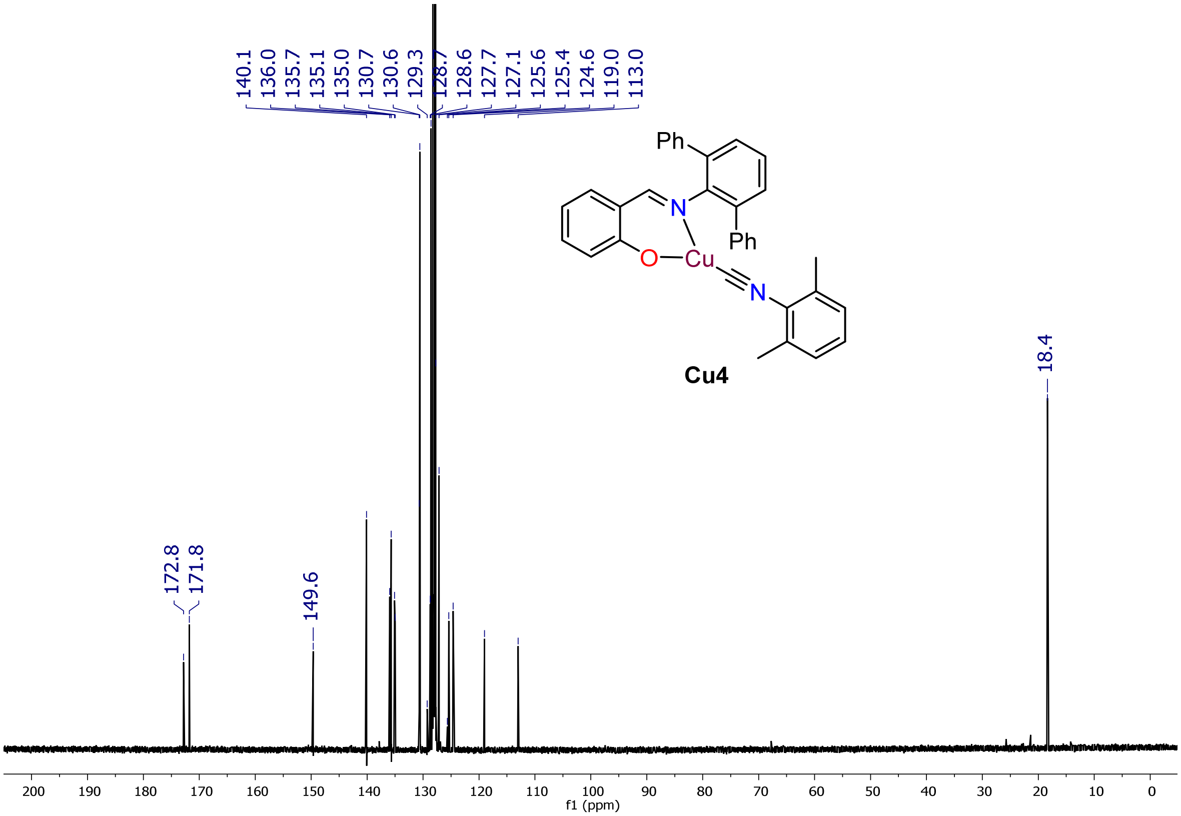

Fig. S38. $^{13}C\{^{1}H\}$ NMR spectrum of **Cu4**, recorded at room temperature in C_6D_6 at 126 MHz.

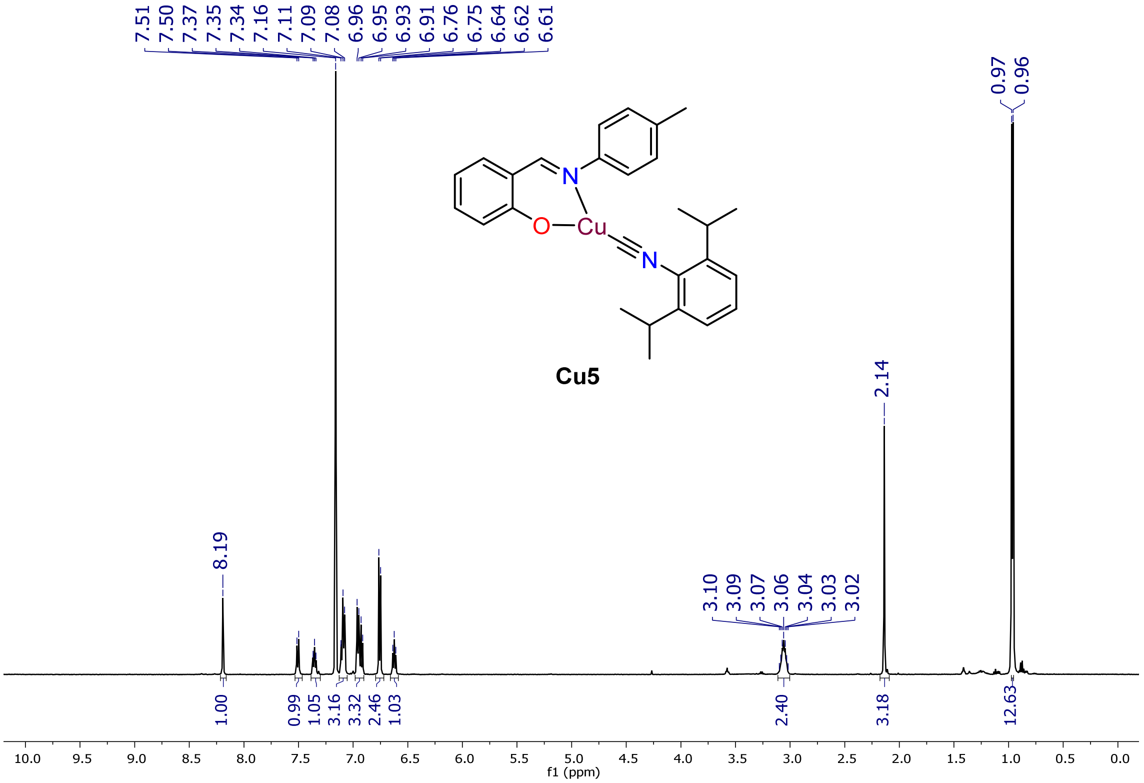

Fig. S39. ^{1}H NMR spectrum of **Cu5**, recorded at room temperature in C_6D_6 at 500 MHz.

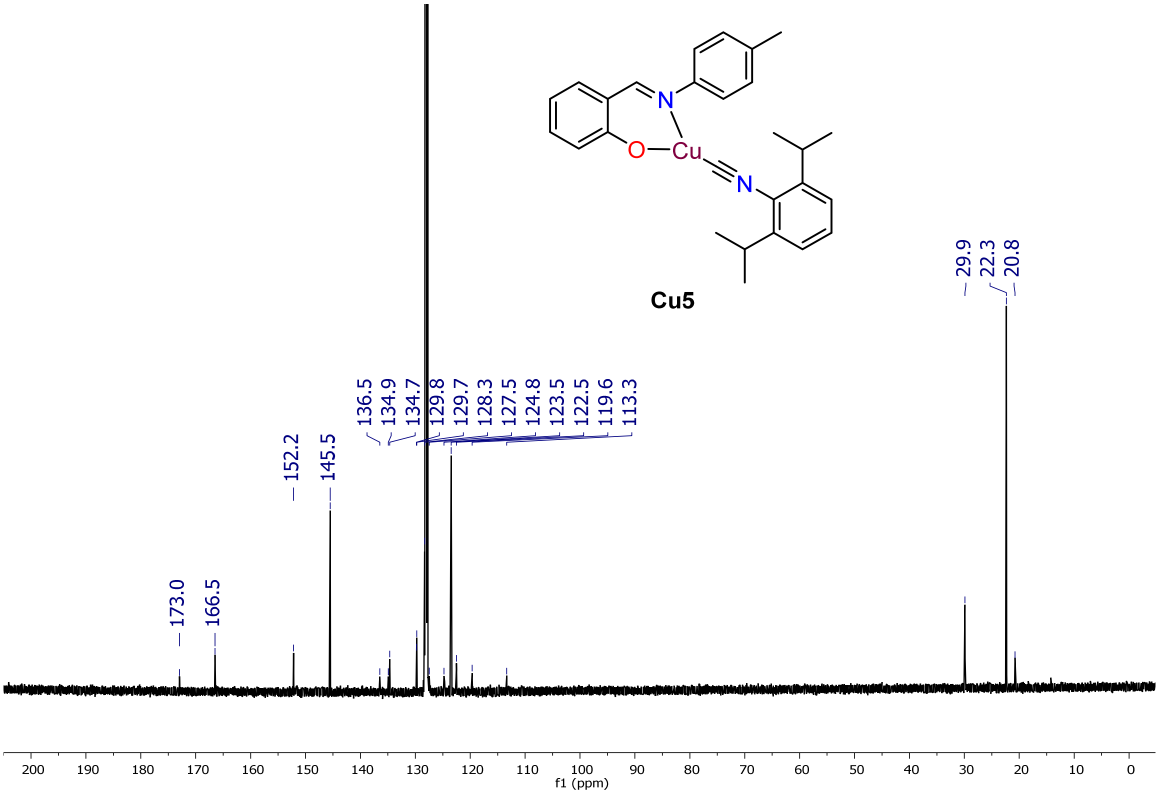

Fig. S40. $^{13}C\{^{1}H\}$ NMR spectrum of **Cu5**, recorded at room temperature in C_6D_6 at 126 MHz.

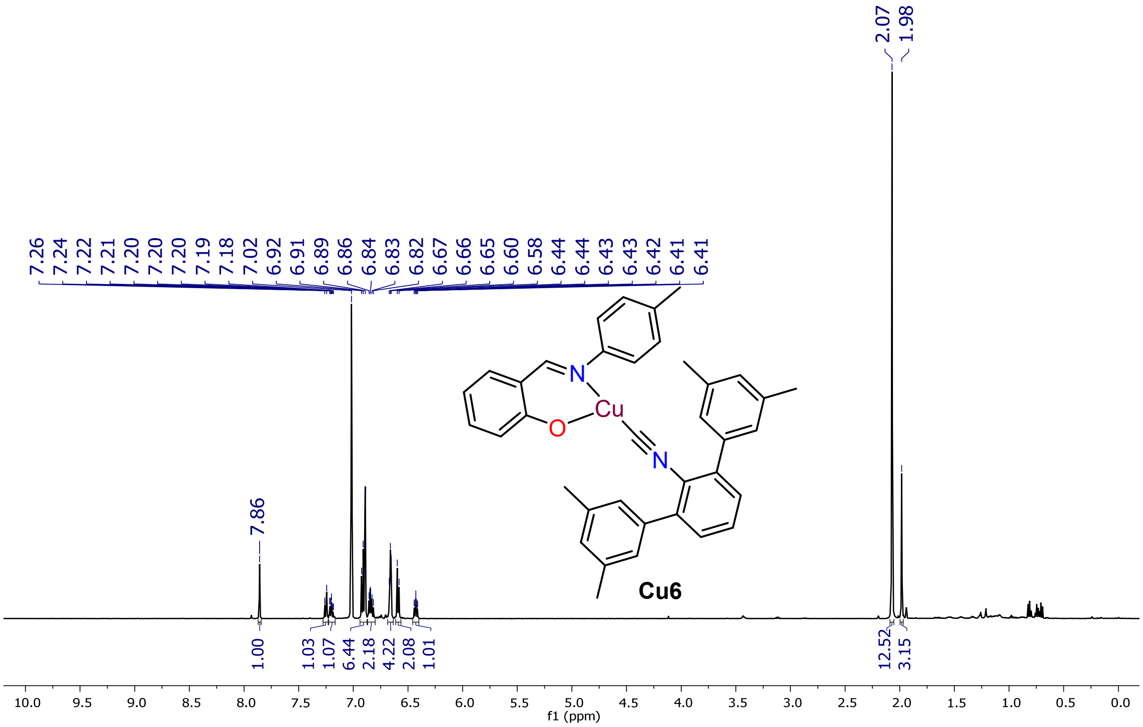

Fig. S41. ^{1}H NMR spectrum of **Cu6**, recorded at room temperature in C_6D_6 at 500 MHz.

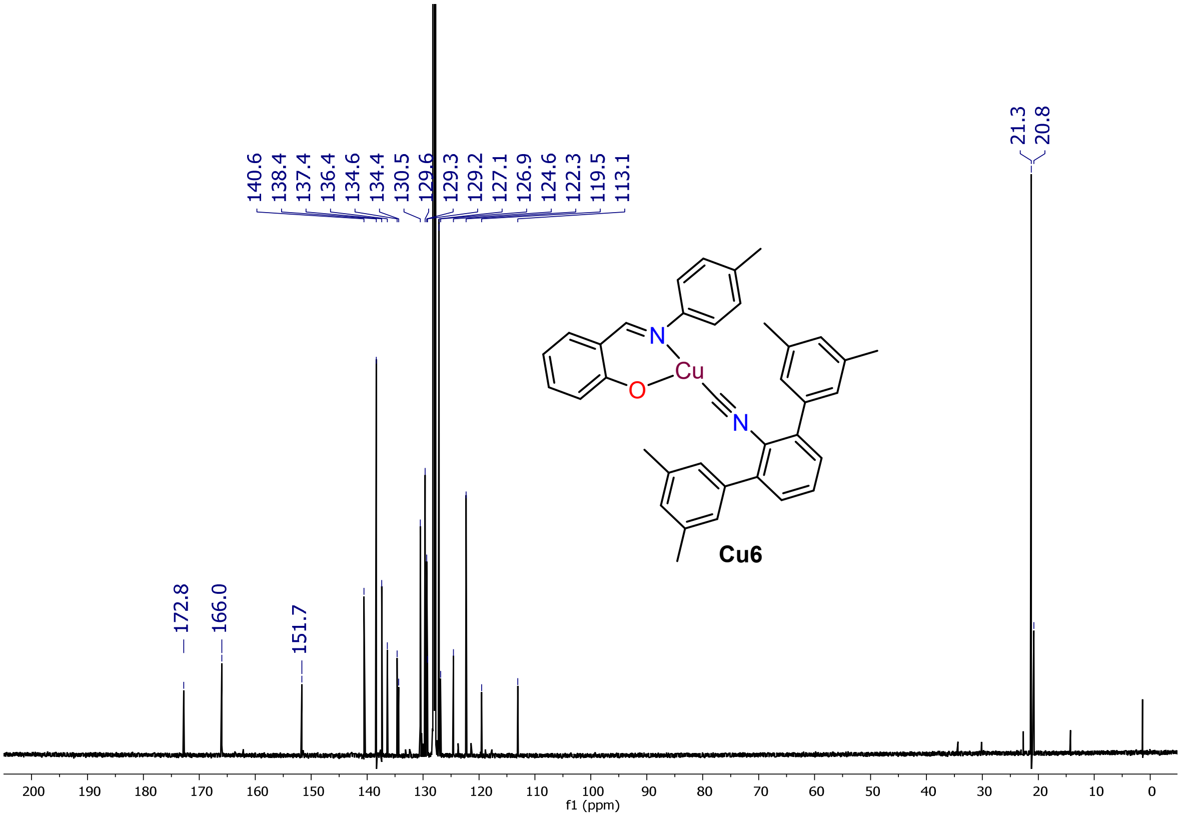

Fig. S42. $^{13}C\{^{1}H\}$ NMR spectrum of **Cu6**, recorded at room temperature in C_6D_6 at 126 MHz.

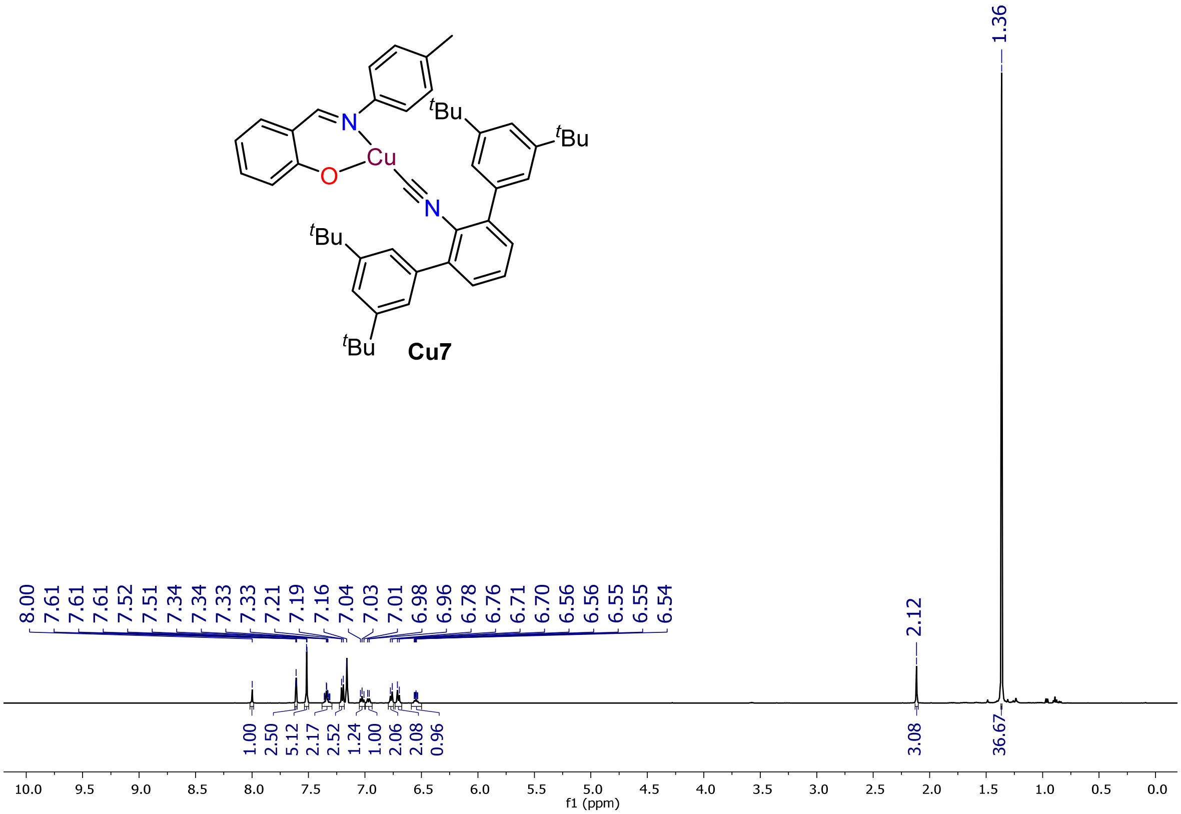

Fig. S43. ^{1}H NMR spectrum of **Cu7**, recorded at room temperature in C_6D_6 at 500 MHz.

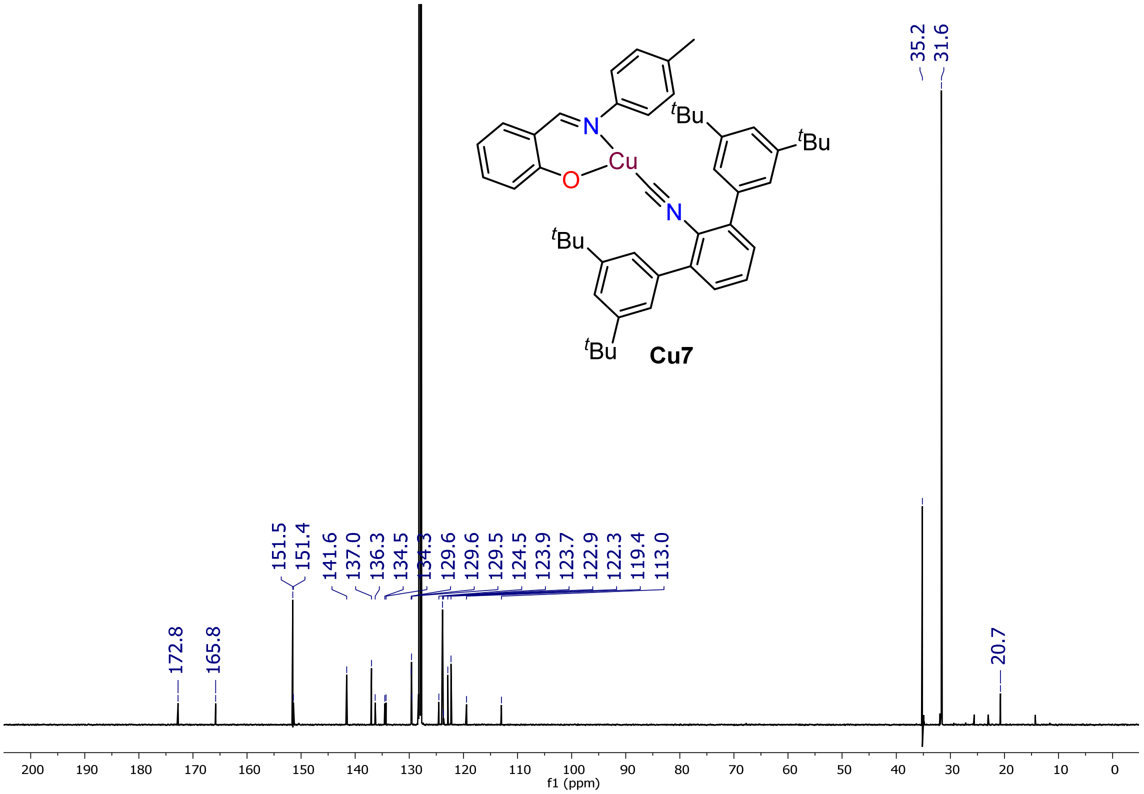

Fig. S44. $^{13}C\{^{1}H\}$ NMR spectrum of **Cu7**, recorded at room temperature in C_6D_6 at 126 MHz.

DFT Calculations

DFT calculations were performed using Gaussian16 quantum chemistry package[6] at B3LYP level of theory.[7] We used LANL2DZ[8] basis set with the relativistic effective core potential for Cu and 6-31+g(d) basis for other elements (H, C, N, O, and F). The geometries were optimized without any symmetry constraints. Geometry optimizations were performed starting from crystal structure of complexes. The molecular orbitals were visualized using IQmol.

Time-dependent density functional theory (TDDFT) and NTO analysis was employed to calculate electronic excitation energies and electron distributions as implemented in Gaussian 16. We used the B3LYP functional in combination with 6-31+g(d) basis set. The conductor-like polarizable continuum model, CPCM was used to model solvation using toluene as solvent.[9]

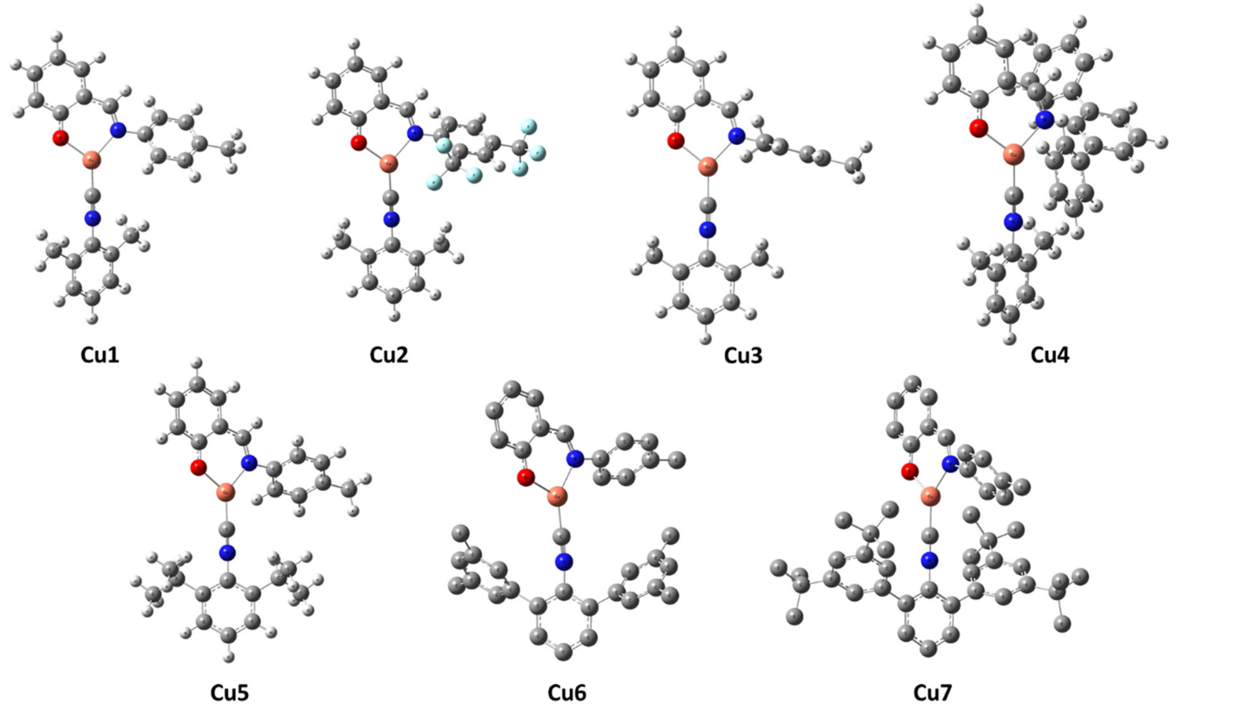

Fig. S45. DFT-optimized structures of **Cu1**–**Cu7**, computed at B3LYP/LANL2DZ(Cu)/6-31+g(d)[C,H,N,O,F] level of theory. Hydrogen atoms are omitted in **Cu6** and **Cu7** for visual clarity.

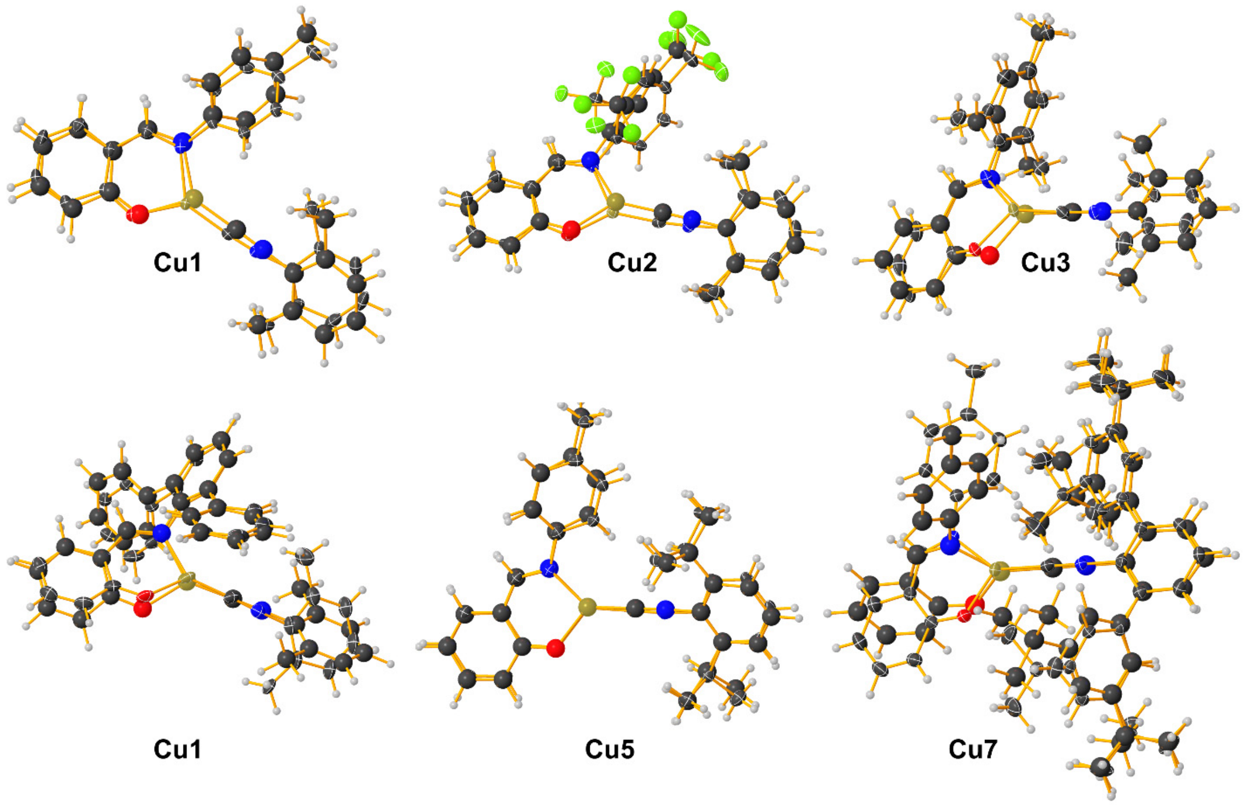

Fig. S46. Molecular structures of **Cu1**–**Cu5** and **Cu7**, determined by single-crystal X-ray diffraction and displayed as thermal ellipsoid plots, overlaid with their DFT-optimized structures, shown as ball-and-stick diagrams.

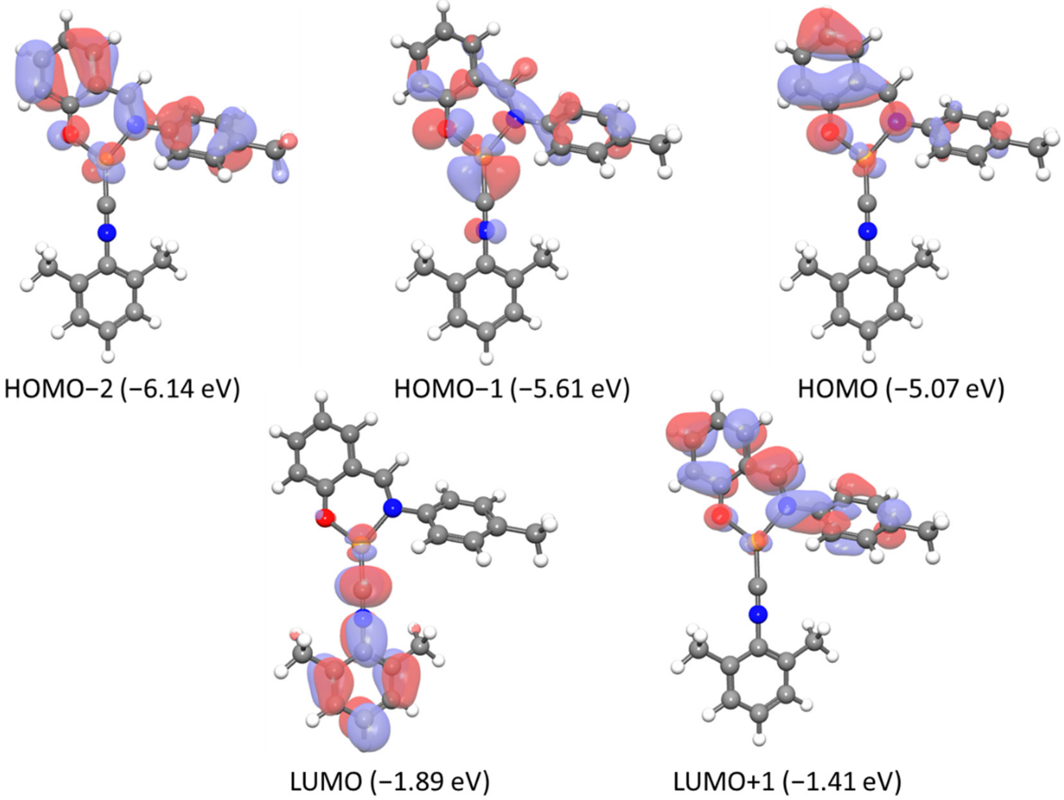

Fig. S47. Calculated frontier molecular orbitals of **Cu1** using B3LYP/6-31+g(d)/LANL2DZ level of theory (iso value = 0.08).

Table S7. Summary of electronic transitions in complex **Cu1**, determined by TD-DFT.

Excited States	Energy / eV	λ / nm	Oscillator Strength (*f*)	Description
1	3.01	411.64	0.0424	HOMO−1 → LUMO (0.48528)<br>HOMO−1 → LUMO+1 (−0.13740)<br>HOMO → LUMO (−0.46721)
2	3.13	396.19	0.0434	HOMO−1 → LUMO (−0.15779)<br>HOMO−1 → LUMO+1 (0.44364)<br>HOMO → LUMO (−0.36062)<br>HOMO → LUMO+1 (−0.37071)
3	3.21	386.56	0.2689	HOMO−1 → LUMO (0.41264)<br>HOMO−1 → LUMO+1 (0.10377)<br>HOMO → LUMO (0.36238)
4	3.27	378.94	0.1056	HOMO−1 → LUMO (0.16781)<br>HOMO−1 → LUMO+1 (0.50407)<br>HOMO → LUMO (0.11127)<br>HOMO → LUMO+1 (0.43244)
5	3.92	316.44	0.0192	HOMO−1 → LUMO (0.49032)<br>HOMO−2 → LUMO (−0.46846)
6	4.04	306.91	0.0400	HOMO−2 → LUMO (−0.28435)<br>HOMO−2 → LUMO+1 (0.20971)

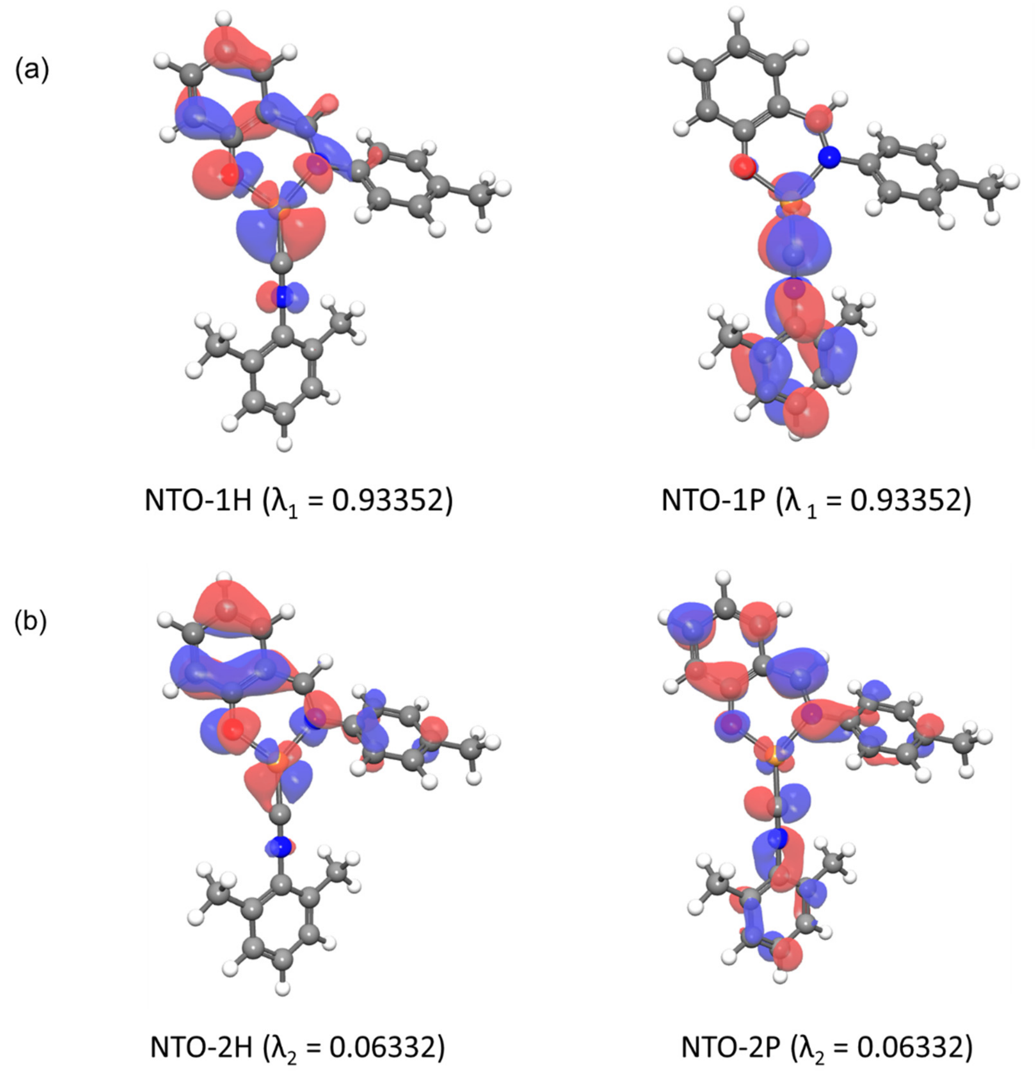

Fig. S48. Natural transition orbitals (NTOs) of **Cu1** calculated for the excited state associated with highest oscillator strength [$S_0 \rightarrow S_3$ excitation (*f* = 0.2689)], using B3LYP/6-31+g(d) level of theory (iso value = 0.08): (a) dominant NTO pair: hole (H) and particle (P) orbitals with singular value $\lambda_1 = 0.93$; (b) Second NTO pair: hole (H) and particle (P) orbitals with singular value $\lambda_2 = 0.06$.

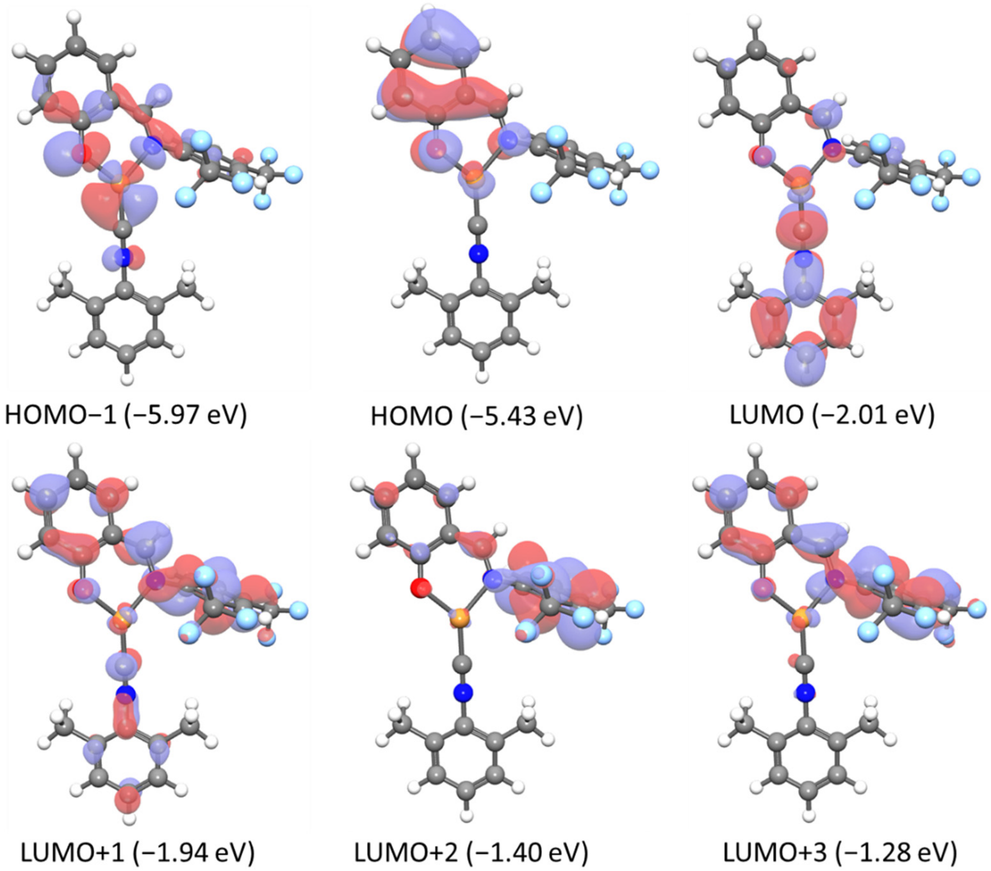

Fig. S49. Calculated frontier molecular orbitals of **Cu2** using B3LYP/6-31+g(d)/LANL2DZ level of theory (iso value = 0.08).

Table S8. Summary of electronic transitions in complex **Cu2**, determined by TD-DFT.

Excited States	Energy / eV	λ / nm	Oscillator Strength (*f*)	Description
1	3.01	411.55	0.1036	HOMO−1 → LUMO (0.55597)<br>HOMO → LUMO (0.40555)
2	3.11	398.41	0.1051	HOMO−1 → LUMO (−0.40368)<br>HOMO → LUMO (0.56553)
3	3.25	381.89	0.0637	HOMO−1 → LUMO+1 (0.56940)<br>HOMO → LUMO+1 (0.38305)
4	3.33	372.57	0.0391	HOMO−1 → LUMO+1 (−0.38315)<br>HOMO → LUMO+1 (0.57997)
5	3.70	335.32	0.0073	HOMO−1 → LUMO (0.10119)<br>HOMO → LUMO (0.67721)<br>HOMO−1 → LUMO+1 (−0.12938)
6	3.79	327.08	0.0069	HOMO → LUMO (0.14111)<br>HOMO → LUMO+1 (0.68574)

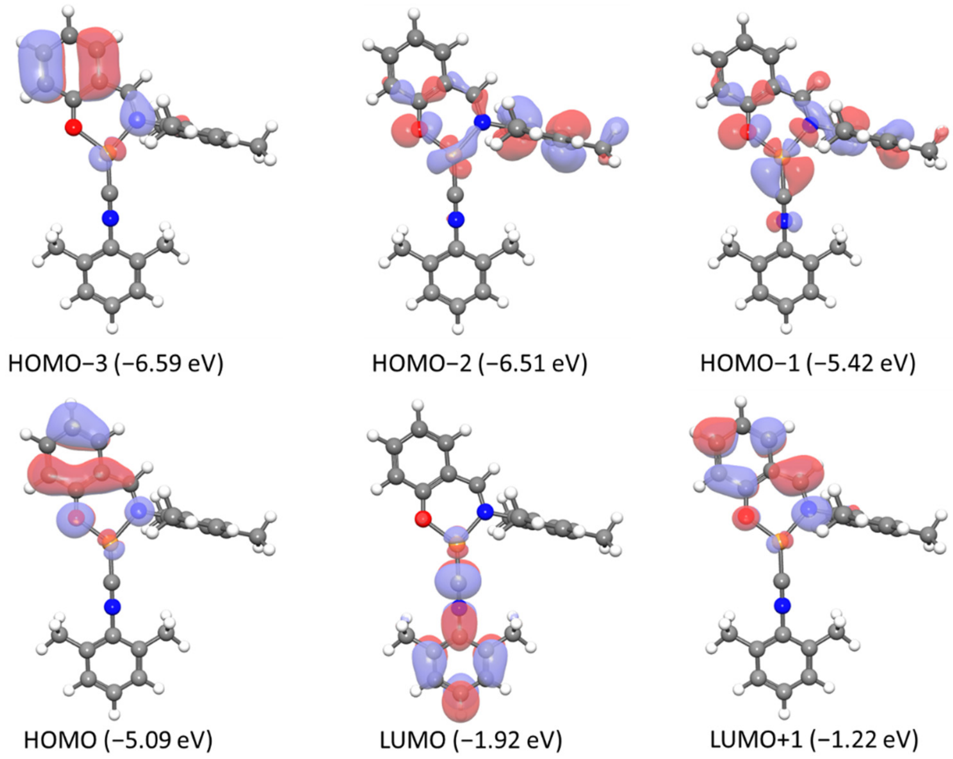

Fig. S50. Calculated frontier molecular orbitals of **Cu3** using B3LYP/6-31+g(d)/LANL2DZ level of theory (iso value = 0.08).

Table S9. Summary of electronic transitions in complex **Cu3**, determined by TD-DFT.

Excited States	Energy / eV	λ / nm	Oscillator Strength (*f*)	Description
2	3.14	395.04	0.1062	HOMO → LUMO (0.70050)
4	3.42	362.29	0.1830	HOMO → LUMO+1 (0.69686)
5	3.92	316.47	0.0076	HOMO−3 → LUMO (0.48373)<br>HOMO−2 → LUMO (0.49613)
6	4.16	297.73	0.0002	HOMO−3 → LUMO (0.40262)<br>HOMO−2 → LUMO (−0.36077)<br>HOMO−2 → LUMO+1 (0.41366)
7	4.19	295.61	0.0220	HOMO−3 → LUMO (−0.10439)<br>HOMO−2 → LUMO (0.55018)<br>HOMO−1 → LUMO (0.21103<br>HOMO → LUMO (−0.29602)

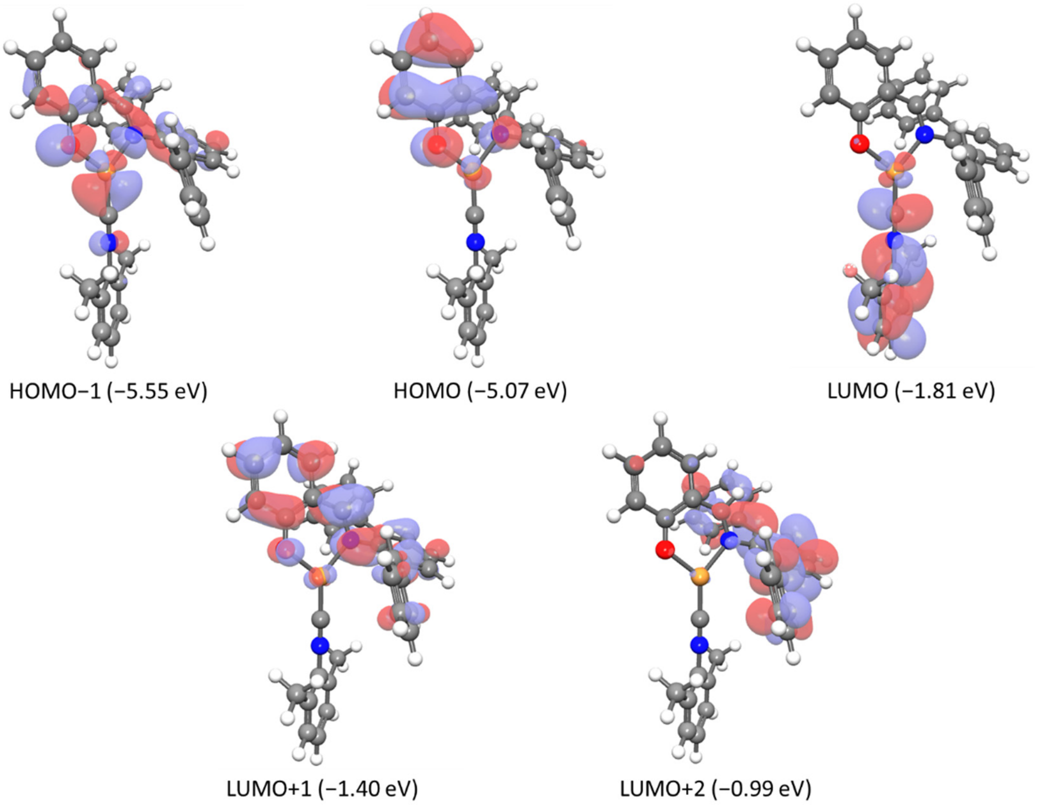

Fig. S51. Calculated frontier molecular orbitals of **Cu4** using B3LYP/6-31+g(d)/LANL2DZ level of theory (iso value = 0.08).

Table S10. Summary of electronic transitions in complex **Cu4**, determined by TD-DFT.

Excited States	Energy / eV	λ / nm	Oscillator Strength (*f*)	Description
1	3.06	405.46	0.0465	HOMO−1 → LUMO (0.44109)<br>HOMO−1 → LUMO+1 (−0.39357)<br>HOMO → LUMO (−0.29307)<br>HOMO → LUMO+1 (0.21406)
2	3.15	393.41	0.0033	HOMO−1 → LUMO (−0.14416)<br>HOMO−1 → LUMO+1 (−0.38457)<br>HOMO → LUMO (0.48504)<br>HOMO → LUMO+1 (0.27837)
3	3.24	382.56	0.1737	HOMO−1 → LUMO (−0.20452)<br>HOMO−1 → LUMO+1 (0.23502)<br>HOMO → LUMO (−0.21827)<br>HOMO → LUMO+1 (0.58369)
4	3.29	376.60	0.1255	HOMO−1 → LUMO (0.43518)<br>HOMO−1 → LUMO+1 (0.34625)
5	3.76	330.05	0.0391	HOMO−1 → LUMO+2 (−0.11777)<br>HOMO → LUMO+2 (0.68937)

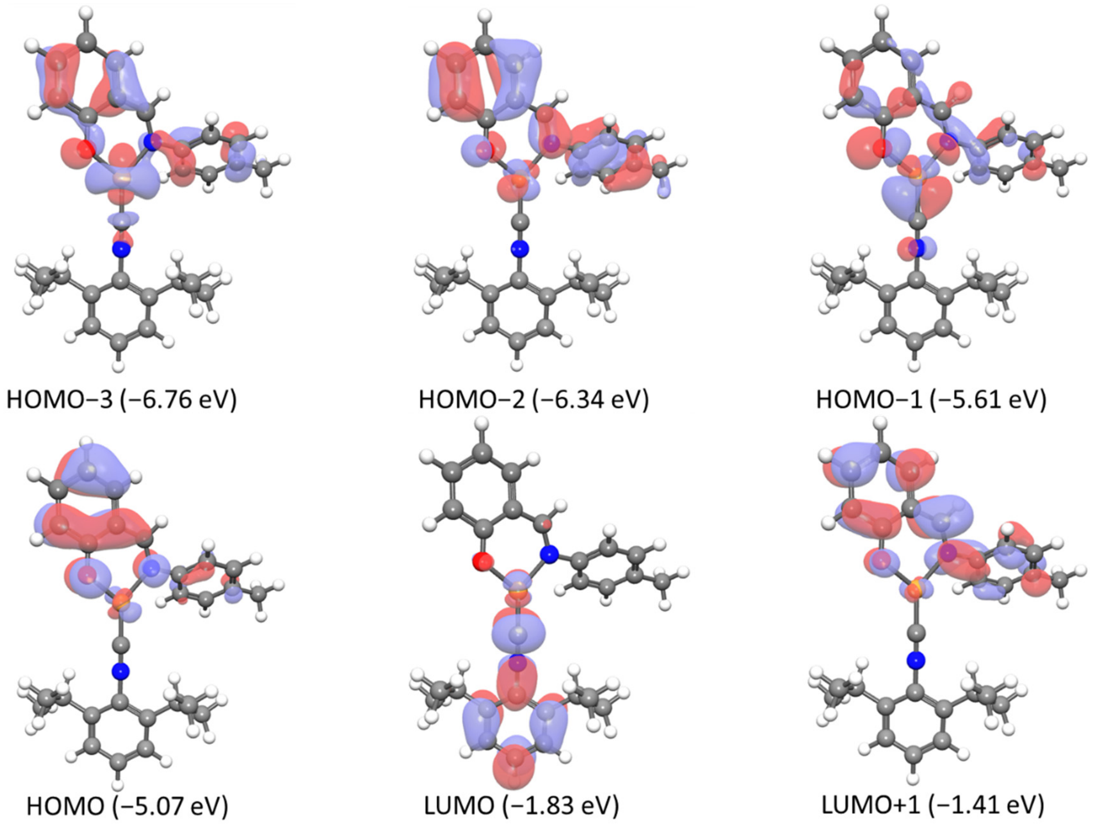

Fig. S52. Calculated frontier molecular orbitals of **Cu5** using B3LYP/6-31+g(d)/LANL2DZ level of theory (iso value = 0.08).

Table S11. Summary of electronic transitions in complex **Cu5**, determined by TD-DFT.

Excited States	Energy / eV	λ / nm	Oscillator Strength (*f*)	Description
4	3.02	410.48	0.0483	HOMO−1 → LUMO (0.51836)<br>HOMO−1 → LUMO+1 (0.12405)<br>HOMO → LUMO (−0.43587)
6	3.14	394.78	0.0413	HOMO−1 → LUMO (0.11678)<br>HOMO−1 → LUMO+1 (0.45147)<br>HOMO → LUMO (0.33473)<br>HOMO → LUMO+1 (−0.39908)
8	3.21	386.63	0.2565	HOMO−1 → LUMO (0.39911)<br>HOMO → LUMO (0.41823)<br>HOMO → LUMO+1 (0.36744)
9	3.28	378.34	0.0992	HOMO−1 → LUMO (−0.15003)<br>HOMO−1 → LUMO+1 (0.50411)<br>HOMO → LUMO (−0.12043)<br>HOMO → LUMO+1 (0.43679)
11	3.93	315.23	0.0237	HOMO−2 → LUMO (0.46574)<br>HOMO−3 → LUMO (0.49141)

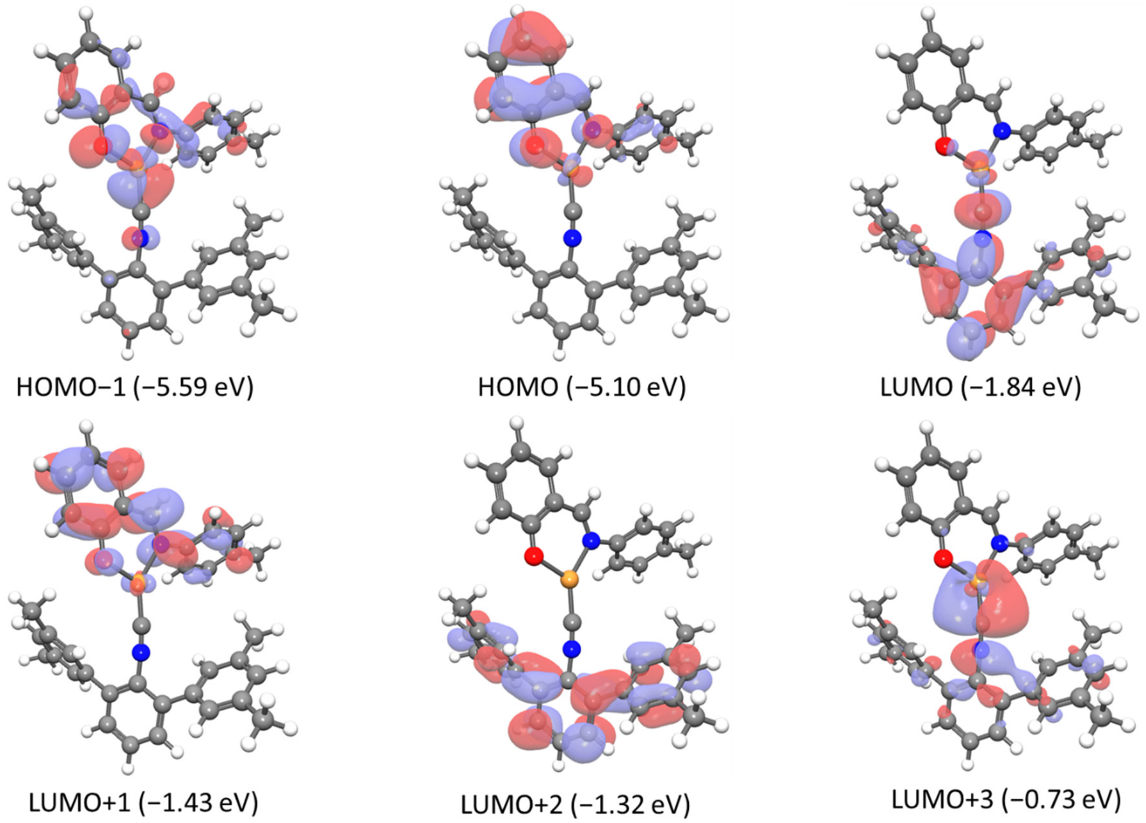

Fig. S53. Calculated frontier molecular orbitals of **Cu6** using B3LYP/6-31+g(d)/LANL2DZ level of theory (iso value = 0.08).

Table S12. Summary of electronic transitions in complex **Cu6**, determined by TD-DFT.

Excited States	Energy / eV	λ / nm	Oscillator Strength (*f*)	Description
1	3.02	410.55	0.0545	HOMO−1 → LUMO (0.34818)<br>HOMO → LUMO (−0.24983)<br>HOMO−1 → LUMO+1 (0.52883)<br>HOMO → LUMO+1 (−0.13673)
2	3.09	400.44	0.0254	HOMO−1 → LUMO (−0.19307)<br>HOMO → LUMO (0.45276)<br>HOMO−1 → LUMO+1 (0.41921)<br>HOMO → LUMO+1 (0.26679)
3	3.23	384.28	0.2606	HOMO−1 → LUMO (0.22416)<br>HOMO−1 → LUMO+1 (−0.18546)<br>HOMO → LUMO+1 (0.62462)
4	3.26	380.23	0.1342	HOMO−1 → LUMO (0.48830)<br>HOMO−1 → LUMO+1 (0.42501)<br>HOMO−1 → LUMO+3 (−0.14974)<br>HOMO → LUMO (−0.17363)

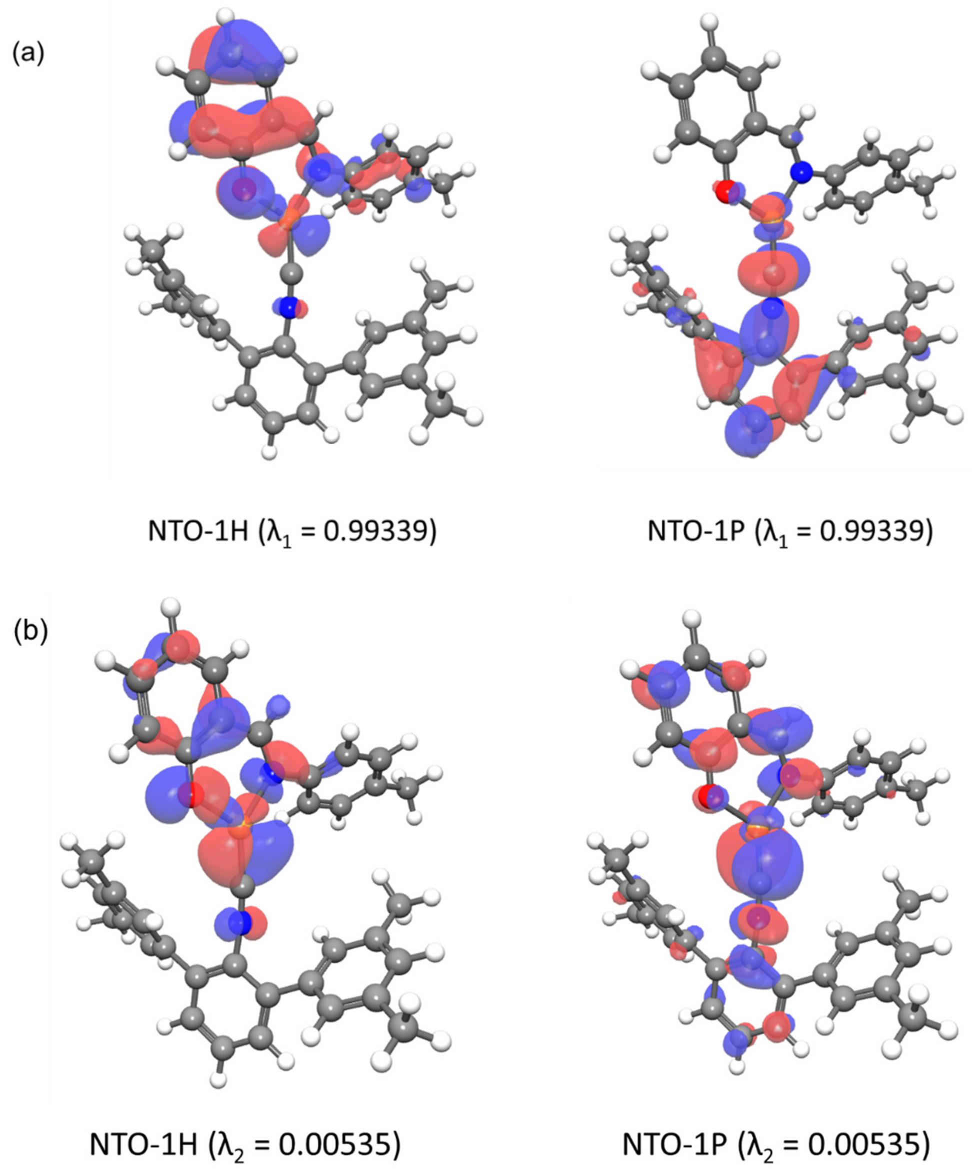

Fig. S54. Natural transition orbitals (NTOs) of **Cu6** calculated for the excited state associated with highest oscillator strength [$S_0 \rightarrow S_3$ excitation (f = 0.2606)], using B3LYP/6-31+g(d) level of theory (iso value = 0.08): (a) dominant NTO pair: hole (H) and particle (P) orbitals with singular value λ_1 = 0.99; (b) Second NTO pair: hole (H) and particle (P) orbitals with singular value λ_2 = 0.01.

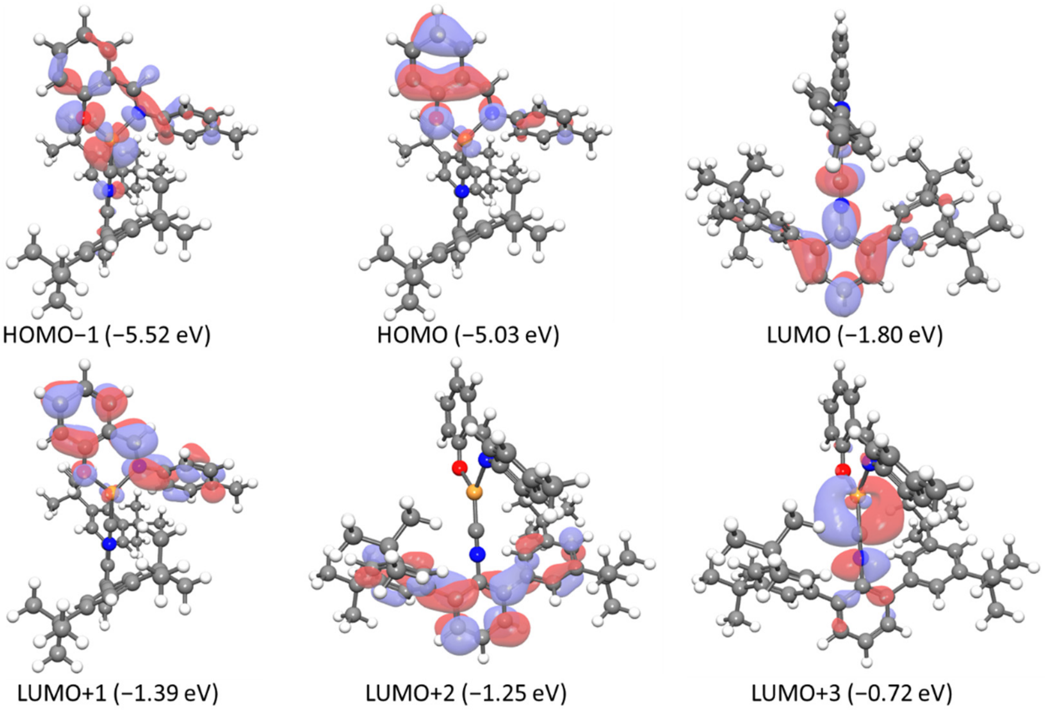

Fig. S55. Calculated frontier molecular orbitals of **Cu7** using B3LYP/6-31+g(d)/LANL2DZ level of theory (iso value = 0.08).

Table S13. Summary of electronic transitions in complex **Cu7**, determined by TD-DFT.

Excited States	Energy / eV	λ / nm	Oscillator Strength (*f*)	Description
1	3.06	405.72	0.1073	HOMO−1 → LUMO+1 (0.53115)<br>HOMO → LUMO (0.18327)<br>HOMO → LUMO+1 (−0.40574)
2	3.11	398.60	0.0249	HOMO−1 → LUMO+1 (−0.18417)<br>HOMO → LUMO (0.67493)
3	3.22	384.82	0.1905	HOMO−1 → LUMO (0.11644)<br>HOMO−1 → LUMO+1 (0.40768)<br>HOMO → LUMO+1 (0.55055)
4	3.32	373.64	0.1955	HOMO−1 → LUMO (0.65668)<br>HOMO−1 → LUMO+3 (0.16625)<br>HOMO → LUMO+1 (−0.13755)
5	3.71	333.89	0.0193	HOMO−1 → LUMO (−0.12564)<br>HOMO−1 → LUMO+3 (0.54259)<br>HOMO → LUMO+1 (−0.39091)

References

1 P. G. Seybold and M. Gouterman, *Journal of Molecular Spectroscopy*, 1969, **31**, 1–13.

2 K. Suzuki, A. Kobayashi, S. Kaneko, K. Takehira, T. Yoshihara, H. Ishida, Y. Shiina, S. Oishi and S. Tobita, *Phys. Chem. Chem. Phys.*, 2009, **11**, 9850.

3 D. Kim and T. S. Teets, *J. Am. Chem. Soc.*, 2024, **146**, 16848–16855.

4 X.-F. Yin, H. Lin, A.-Q. Jia, Q. Chen and Q.-F. Zhang, *Journal of Coordination Chemistry*, 2013, **66**, 3229–3240.

5 G. M. Sheldrick, *Acta Crystallogr A Found Crystallogr*, 2008, **64**, 112–122.

6 M. J. Frisch, G. W. Trucks, H. B. Schlegel, G. E. Scuseria, M. A. Robb, J. R. Cheeseman, G. Scalmani, V. Barone, G. A. Petersson, H. Nakatsuji, X. Li, M. Caricato, A. V. Marenich, J. Bloino, B. G. Janesko, R. Gomperts, B. Mennucci, H. P. Hratchian, J. V. Ortiz, A. F. S44 Izmaylov, J. L. Sonnenberg, D. Williams-Young, F. Ding, F. Lipparini, F. Egidi, J. Goings, B. Peng, A. Petrone, T. Henderson, D. Ranasinghe, V. G. Zakrzewski, J. Gao, N. Rega, G. Zheng, W. Liang, M. Hada, M. Ehara, K. Toyota, R. Fukuda, J. Hasegawa, M. Ishida, T. Nakajima, Y. Honda, O. Kitao, H. Nakai, T. Vreven, K. Throssell, J. A. Montgomery, Jr., J. E. Peralta, F. Ogliaro, M. J. Bearpark, J. J. Heyd, E. N. Brothers, K. N. Kudin, V. N. Staroverov, T. A. Keith, R. Kobayashi, J. Normand, K. Raghavachari, A. P. Rendell, J. C. Burant, S. S. Iyengar, J. Tomasi, M. Cossi, J. M. Millam, M. Klene, C. Adamo, R. Cammi, J. W. Ochterski, R. L. Martin, K. Morokuma, O. Farkas, J. B. Foresman, and D. J. Fox, *Gaussian 16 Rev. C.01, 2016*.

7 A. D. Becke, *The Journal of Chemical Physics*, 1993, **98**, 5648–5652.

8 K. E. Riley and K. M. Merz, *J. Phys. Chem. A*, 2007, **111**, 6044–6053.

9 A. Klamt and G. Schüürmann, *J. Chem. Soc., Perkin Trans. 2*, 1993, 799–805.
